# Synthesis and Reactivity of Triphosphaallyl Cation Stabilized by N‐Heterocyclic Carbenes

**DOI:** 10.1002/chem.202501311

**Published:** 2025-06-04

**Authors:** Julia Frötschel‐Rittmeyer, Felix Hennersdorf, Jannis Fidelius, Chris Sala, Christoph Ziegler, Michael Holthausen, Kai Schwedtmann, Robert Wolf, Jan J. Weigand

**Affiliations:** ^1^ Faculty of Chemistry and Food Chemistry Technische Universität Dresden 01062 Dresden Germany; ^2^ Institute of Inorganic Chemistry University of Regensburg 93040 Regensburg Germany

**Keywords:** cyclic phosphorus scaffolds, gallium‐phosphorus bonding, N‐heterocyclic carbene, P‐C bond activation, triphosphaallyl cation

## Abstract

Reduction of the triphosphaallyl species **6**[GaCl_4_] with Ga^I^[Ga_2_Cl_7_] affords the imidazoliumyl‐substituted (L) bicyclo[2.1.1]‐triphosphane **10**[Ga_2_Cl_7_], featuring an unprecedented Ga_2_Cl_5_‐bridged P_3_ scaffold. Reactions of **10**[Ga_2_Cl_7_] with nucleophiles (Cl^−^ or NHC) result in rare, selective P–C bond cleavages, affording Ga_2_Cl_y_‐substituted triphosphiranes (LP_3_Ga_2_Cl_y_, y =  5, 6) via an intramolecular ring closure mechanism. Protonation of **6**[GaCl_4_] gives rise to a similar ring closure, but without P–C bond cleavage, to afford the L_2_P_3_H^+^ salt **8**[OTfGaCl_3_]_2_. Additionally, the palladium complex **26**[GaCl_4_], formed through the reaction of **10**[Ga_2_Cl_7_] with [Pd(PPh_3_)_4_], presents a novel bicyclic P_3_Pd moiety (LP_3_Pd(PPh_3_)_2_[GaCl_4_]). Comprehensive DFT calculations have been conducted to elucidate the bonding situation in **26**[GaCl_4_], uncovering significant metal‐to‐ligand π‐back‐donation and a distinctive 3‐center‐4‐electron hyperbonding phenomenon in the P₃Pd framework. These findings offer valuable insights into chemistry of cyclic polyphosphorus compounds and, in particular, the reactivity, structural flexibility, and the coordination properties of cationic triphosphorus species.

## Graphical abstract

Imidazoliumyl‐substituted triphosphaallyl cations undergo selective P–C bond cleavage and form unprecedented bicyclic P₃‐Ga scaffolds with unique reactivity.

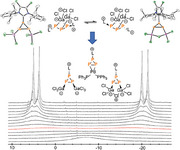

## 1 Introduction

**Figure 1:**
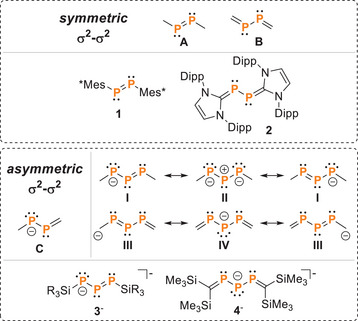
Selected examples of compounds containing a symmetric or asymmetric σ^2^‐σ^2^ bonded P‐P unit and resonance structures of triphosphaallyl (**I**, **II**) and triphosphapentadienyl (**III**, **IV**) compounds.

The development of novel acyclic and cyclic polyphosphanes has been a long‐standing subfield of phosphorus chemistry, which nowadays requires new, selective synthetic methodologies.^[^ ^1^ ^]^ Our group has long been interested in compounds featuring multiple P–P bonds, which display a great variety of bonding modes.^[^ ^1^ ^]^ Of particular interest to us are phosphorus compounds that exhibit a σ^2^‐σ^2^ bonding motif. These can exist, for example, in symmetric, neutral combination, as represented by species **A** and **B** (Figure 1), which are found in diphosphenes and 1,2‐di(ylidene)diphosphanes, respectively. Diphosphene **1**, first described by Yoshifuji in 1981,^[^ ^2^ ^]^ was the first isolated compound with a formal P ═ P double bond, where the steric bulk of Mes* (2,4,6‐tri‐tert‐bu‐tylphenyl) plays a crucial role in stabilization. Different from most alkenes, these compounds feature a smaller HOMO‐LUMO gap, resulting in electronic absorptions in the visible part of the electromagnetic spectrum. Carbene‐stabilized main group diatomic motifs (Si_2_,^[^ ^3^ ^]^ P_2_,^[^ ^4^ ^]^, and As_2_^[^ ^5^ ^]^) were later introduced by Robinson's group, with compound **2** (Dipp ═ 2,6‐diisopropylphenyl) serving as an example of a 1,2‐di(ylidene)diphosphane. Both classes of compounds have been subject of intense investigations in recent years,^[^ ^6^ ^]^ particularly in terms of oxidation, the isolation of cationic P_2_ species,^[^ ^7^ ^]^ and the first examples of cationic diphosphenes.^[^ ^8^, ^9^ ^]^ Phosphanido‐substituted diphosphenes, closely related to these species, exist in asymmetric charged combinations as depicted in the general representation **C** (Figure 1). Notably, in anionic derivatives **3** ^−^ and **4** ^−^,^[^ ^10^ ^]^ the negative charge on the phosphanido moiety undergoes resonance stabilization similar to that seen in allyl (resonance structures **I** and **II**) and pentadienyl (resonance structures **III** and **IV**) systems in carbon chemistry. The bonding situation in these compounds strongly depends on the substitution patterns. For example, anion **3** ^−^ is best described by resonance structures **I** and **II**, while anion **4** ^−^ aligns more closely with structures **III** and **IV**, resembling a 1,3‐dimethylenetriphosphane‐2‐ide with formal charges on the central phosphorus or carbon atoms (Figure 1).

**Scheme 1:**
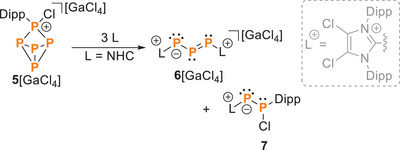
Reaction of **5**[GaCl_4_] with NHC L in a 1:3 ratio to **6**[GaCl_4_] and **7**. C_6_H_5_F, −40 °C, 5 minutes, −40 °C to rt, 3 hours; **6** (87 %), **7**[GaCl_4_] 89 %; L = 1,3‐Bis[2,6‐diisopropylphenyl]‐4,5‐dichloro‐1,3‐dihydro‐2H‐imidazol‐2‐ylidene.^[^ ^11^ ^].^

**Scheme 2:**
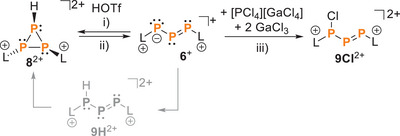
Reversible protonation (HOTf) and deprotonation (**L**) of **6** ^+^ and **8** ^2+^. i) +HOTf, CH_2_Cl_2_, rt, ii) +L, −[LH][OTf], CH_2_Cl_2_, RT;^[^ ^14^ ^]^ Reaction of **6** ^+^ with the Cl^+^‐source [PCl_4_][GaCl_4_] in the presence of GaCl_3_. iii) + [PCl_4_][GaCl_4_], + 2 GaCl_3_, – PCl_3_, CH_2_Cl_2_, rt.

We and others reported the first example triphosphaallyl cations, that is, the chloride salt **6**[Cl] and the tetrachlorogallate salt **6**[GaCl_4_].^[^ ^11^, ^12^ ^]^ **6**[GaCl_4_] was obtained together with the neutral P_2_ compound **7** from the [3 + 2] fragmentation of compound **5**[GaCl_4_] (Dipp ═ 2,6‐diisopropylphenyl) by the N‐heterocyclic carbene (NHC) **L** (3 equiv., Scheme 1). Here, we describe the protonation of [GaCl_4_] with HOTf to form the cyclic dication **8** ^2+^ (Scheme 2). This cyclization reaction of the triphosphaallyl cation **6** ^+^ and its three low‐coordinated phosphorus atoms presents several possibilities for subsequent reactions and coordination chemistry. Cation **6** ^+^, the first example of a triphosphaallyl derivative not stabilized in the coordination sphere of a metal atom, exhibits bidentate coordination abilities toward lewis acids such as Ga(I). The reaction of **6**[GaCl_4_] with the Ga(I) source Ga[Ga_2_Cl_7_]^[^ ^13^ ^]^ in CH_2_Cl_2_ or fluorobenzene affords **10**[Ga_2_Cl_7_], a salt with an unprecedented bicyclo[2.1.1]‐backbone. Our studies have shown that cation **10** ^+^ undergoes unusual protonation reactions, yielding the first examples of cationic triphosphanes and selective cleavage of one imidazoliumyl substituent to form an unprecedented *cyclo*‐triphosphane‐1,2‐diide of the type [LP_3_]^−^, which exhibits interesting coordination behavior toward GaCl_3_ and Pd(0).

## 2 Results and Discussion

**Figure 2:**
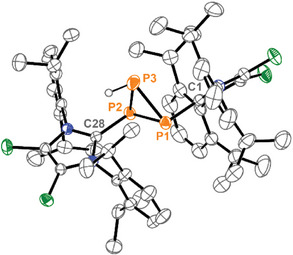
Molecular structure of cation **8** ^2+^ in **8**[OTfGaCl_3_]_2_, hydrogen atoms are omitted for clarity and thermal ellipsoids are displayed at 50% probability; selected bond lengths [Å] and angles [°]: C1–P1 1.853(3), C28–P2 1.851(3), P1–P2 2.221(1), P1–P3 2.206(2), P2–P3 2.214(2), C1–P1–P2 100.66(8), C1–P1–P3 96.06(12), P2–P1–P3 60.02(4), P1–P2–P3 59.65(5), P1–P3–P2 60.33(4), C28–P2–P1 102.25(8), C28–P2–P3 97.05(10).

Initially, we investigated adding HOTf to a deep green solution of **6**[GaCl_4_], which immediately results in the formation of an orange oil. The ^31^P{^1^H} NMR spectrum of this oil reveals an AMX spin system (*δ*(P_A_) = −202.3 ppm, *δ*(P_M_) = −182.6 ppm, *δ*(P_X_) = −156.8 ppm; ^1^ *J*(P_A_P_M_) = −158.8 Hz, ^1^ *J*(P_A_P_X_) = −130.4 Hz, ^1^ *J*(P_M_P_X_) = −203.0 Hz, ^1^ *J*(P_A_H) = 155.7 Hz, ^2^ *J*(P_M_H) = 17.8 Hz, ^2^ *J*(P_X_H) = 34.2 Hz), which can be unambiguously assigned to the cyclotriphosphanediium cation **8** ^2+^ (Scheme 2).^[^ ^14^ ^]^ Recrystallization by diffusion of *n*‐hexane into a solution of the product in 1,2‐C_6_H_4_F_2_ yielded crystals of the dication **8** ^2+^ as a [OTf‐GaCl_3_]‐salt, suitable for crystal structure analysis (Figure 2). The molecular structure of **8** ^2+^ confirms the *trans*‐arrangement of the imidazoliumyl‐substituents (C28–P2–P1–C1: 178.1(1)°), and the bond lengths and angles in the *cylco*‐P_3_ core are similar to those observed in (*t*‐BuP)_3_^[^ ^15^ ^]^ and (MesP)_3_.^[^ ^16^ ^]^ Protonation of **6** ^+^ likely proceeds via the phosphanyl‐substituted diphosphene **9H** ^2+^, and this process is reversible. The addition of NHC **L** to a solution of **8** ^2+^ in CH_2_Cl_2_ immediately yields a deep green‐colored reaction mixture indicating the reformation of **6** ^+^. This procedure can be repeated several times, and monitoring of the multiple protonation/deprotonation reactions by ^31^P{^1^H} NMR spectroscopy does not reveal any significant decomposition.

In order to study the reactivity of **6**[GaCl_4_] with another electrophile, we chose [PCl_4_][GaCl_4_] as a Cl^+^‐source. Reaction of **6**[GaCl_4_] and [PCl_4_][GaCl_4_] in the presence of additional GaCl_3_ affords the chlorinated product **9Cl** ^2+^ accompanied by the formation of PCl_3_. The nucleophilic site of **10** ^+^ bearing the formal negative charge in the Lewis structure is chlorinated to give the dicationic phosphanyldiphosphene **9Cl** ^2+^ (Scheme 2). This outcome is closely related to the intermediately formed protonated compound **9H** ^2+^ and, thus, strongly supports the supposed reaction sequence from **6** ^+^ to **8** ^2+^. The ^31^P NMR spectrum exhibits an AMX spin system with three dd resonances at *δ*  = 56.0, 454.8, and 533.8 ppm. The low‐field signals M and X are assigned to the diphosphene moiety with ^1^ *J*(PP)  = −560 Hz. Multiplet A exhibits two large coupling constants of the magnitudes 202 (P_A_P_X_) and 714 Hz (P_A_P_M_).

The molecular structure of dicationic **9Cl** ^2+^ was obtained by vapor diffusion of *n*‐pentane into *o*‐difluorobenzene of the crude product mixture. **9Cl** ^2+^ crystallizes as racemic salt with both tetrachlorogallate and heptachlorodigallate as counterions (see Supporting Information, Figure S3).

**Scheme 3:**
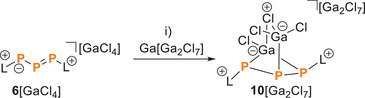
Reaction of Ga[Ga_2_Cl_7_] with **6**[GaCl_4_] to **10**[Ga_2_Cl_7_]. C_6_H_5_F, −40 °C to rt; extraction with *n*‐hexane/CH_2_Cl_2_ (3:2); 55 %.

**Figure 3:**
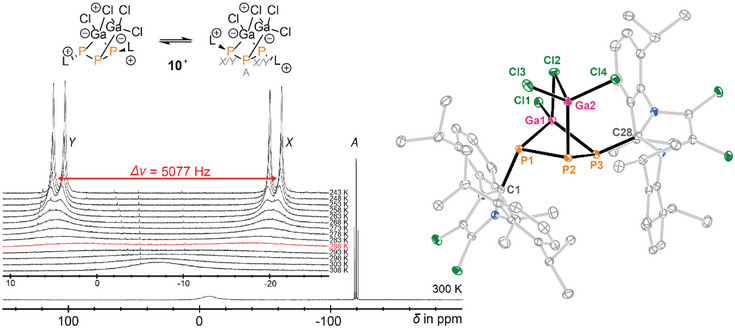
Left: ^31^P{H} NMR spectrum (300 K) and VT measurements (insert, 243 to 308 K of **10**[Ga_2_Cl_7_] in CD_2_Cl_2_); right: molecular structure of cation **10** ^+^ in **10**[Ga_2_Cl_7_]·CH_2_Cl_2_, hydrogen atoms, anions and solvate molecules are omitted for clarity and thermal ellipsoids are displayed at 50% probability; selected bond lengths [Å] and angles [°]: P1–P2 2.2102(8), P2–P3 2.2500(9), P1–Ga1 2.3932(8), P3–Ga1 2.3371(7), P2–Ga2 2.3571(7), P1–C1 1.829(3), P3–C28 1.839(3), Ga1–Cl1 2.1372(8), Ga1–Cl2 2.3148(7), Ga2–Cl2 2.3816(7), Ga2–Cl3 2.1591(8), Ga2–Cl4 2.1558(7), P1–P2–P3 92.76(3), P1–Ga1–P3 86.09(3), Ga1–Cl2–Ga2 94.14(3), Cl1–Ga1–Cl2 103.93(3), P2–Ga2–Cl2 103.67(3), Cl3–Ga2–Cl4 111.00(3).

From these studies, we learned that resonance structures **I** and **II** of the central dicoordinated phosphorus atom participate in a conventional conjugated allylic system, using its p‐type orbitals for both π‐ and σ‐bonds. Therefore, the s‐type electron lone pair of the central phosphorus atom is unavailable for electrophiles, but those on the adjacent phosphorus atoms are accessible. To further investigate the reactivity of **6**[GaCl_4_], we performed the reaction with one equiv. of Ga^I^[Ga_2_Cl_7_] in fluorobenzene (Scheme 3). After removing all volatiles and extracting the residue with a mixture of *n*‐hexane and dichloromethane (3:2), we obtained salt **10**[Ga_2_Cl_7_] (2.16 g, 55 % yield). The molecular structure of **10**[Ga_2_Cl_7_] was determined by single‐crystal X‐ray analysis (Figure 3, right) of yellow crystals obtained by diffusion of *n*‐pentane into an *n*‐hexane/CH_2_Cl_2_ solution at −30 °C. Compared to **6** ^+^, the P–P bond lengths in **10** ^+^ fall within the range of single bonds (Σ*r*_cov_  =  2.22 Å;^[^ ^17^ ^]^ **6** ^+^: average 2.092 Å^[^ ^11^ ^]^), with P1–P2 being slightly shorter (2.2102(9) Å) than P2–P3 (2.2500(9) Å), and the P1–P2–P3 bond angle (92.76(3)°) is less acute than in **6** ^+^ (87.2(4)°). The average P–C bond lengths (1.833(3) Å) are slightly elongated compared to **6** ^+^ (average 1.815 Å), indicating the loss of conjugation as the triphosphaallyl system is involved in coordination. Additionally, the cation contains three P–Ga bonds to two different gallium atoms (P1–Ga1 2.3931(8) Å, P3–Ga1 2.3372(7) Å, P2–Ga2 2.3572(7) Å), each displaying a *pseudo*‐tetrahedral environment (Ga1: P1–Ga1–P3 86.09(2)°, Cl1–Ga1–Cl2 103.93(3)°; Ga2: P2–Ga2–Cl2 103.67(3)°, Cl3–Ga2–Cl4 111.00(3)°). Consistent with [Ga_2_Cl_7_]^−^ anions,^[^ ^18^ ^]^ the Ga–Cl bond lengths vary, based on coordination number of the chlorine atom.

**Scheme 4:**
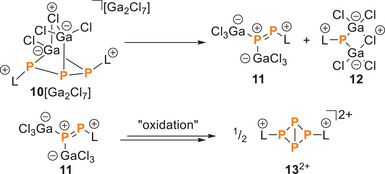
Presumed degradation of **10**[Ga_2_Cl_7_] to **11** and **12** and oxidation of the latter to dication **13** ^2+^.

The ^31^P NMR spectrum of dissolved **10**[Ga_2_Cl_7_] in CD_2_Cl_2_ at 300 K shows a triplet resonance at δ(P_A_)  =  −119.5 ppm with a ^1^ *J*(P_A_P_X_) coupling constant of −274 Hz, assigned to the central phosphorus atom. A broad unresolved signal at δ(P_X_)  =  −7.2 ppm (ν_1/2_ = 2800 Hz) suggests dynamic behavior of **10** ^+^ in solution (Figure 3, left). Both resonances integrate in a ratio of 1:2, indicating a *pseudo*‐AX_2_ spin system. Upon cooling to 243 K, an AXY spin system resolves and the broad resonances separate into two sharp doublets. Using the gutowsky‐holm equation,^[^ ^19^ ^]^ we estimated the free enthalpy of activation (ΔG^≠^) for this process from the coalescence temperature (288 K), which corresponds to the separation of the signals with a large frequency of 5077 Hz. ΔG^≠^ for this process is 48 kJ mol^−1^, comparable to a gallium‐substituted phosphorus atom of an octaphosphane recently reported by our group.^[^ ^20^ ^]^ This fluxional behavior is best explained by looking at the solid‐state structure of the cation **10** ^+^. While the parent cation **6** ^+^ is symmetrical, **10** ^+^ contains three independent phosphorus atoms with the imidazoliumyl substituents oriented both toward (*exo*) and away from (*endo*) the [GaCl]‐bridge in the bicyclic system (Scheme 4).

**Figure 4:**
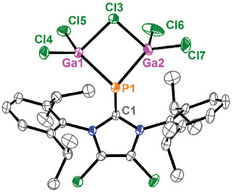
Molecular structure of **12**, hydrogen atoms are omitted for clarity and thermal ellipsoids are displayed at 50 % probability; selected bond lengths [Å] and angles [°]: P1–Ga1 2.3228(7), P1–Ga2 2.3351(7), P1–C1 1.829(3), Ga1–Cl3 2.3562(10), Ga2–Cl3 2.4129(13), Ga1–Cl4 2.1452(9), Ga1–Cl5 2.1636(8), Ga2–Cl6 2.1335(8), Ga2–Cl7 2.1632(10), Ga1–P1–Ga2 84.91(3), Ga1–Cl3–Ga2 82.48(4), P1–Ga1–Cl3 89.83(4), Cl4–Ga1–Cl5 114.58(4), P1–Ga2–Cl3 88.16(3), Cl6–Ga2–Cl7 109.99(4).

In **10** ^+^, the Ga1–Cl1 bond is the shortest (2.1371(7) Å), while the Ga2–Cl2 bond (2.3816(7) Å) is the longest. In solution, an equilibrium between the *S*,*S*/*R,R*‐ and the *R*,*S*/*S,R*‐diastereomers is rapidly established through inversion of the carbon‐bonded phosphorus atoms. Given the moderate yield of **10**[Ga_2_Cl_7_] (55 %), we investigated the reaction further and identified at least three additional by‐products in relatively low concentrations (see Figure S5). Notably, as the reaction proceeds, the solution turns intensely red, accompanied by the formation of a red amorphous solid, followed by the precipitation of crystalline **10**[Ga_2_Cl_7_]. In line with the color change, the ^31^P{^1^H} NMR spectrum revealed resonances consistent with an unsymmetrically substituted P_2_ compound displaying diphosphene character (δ(P_A_)  =  397.2 ppm and δ(P_X_)  =  604.2 ppm; ^1^ *J*(P_A_P_X_)  =  ‐577 Hz),^[^ ^13^ ^]^ which we assign to compound **11** or a related species. Similar observations have been made for other diphosphene compounds derived from **6** ^+^.^[^ ^9^ ^]^ If this compound results from the fragmentation of **10** ^+^, a corresponding [P_1_]‐unit should form simultaneously. The ^31^P{^1^H} NMR spectrum of the reaction mixture shows several singlets, which could correspond to such a species, but we could not assign these resonances as yet. However, single‐crystal X‐ray diffraction analysis on a small amount of crystals from the mother liquors from recrystallization of **10**[Ga_2_Cl_7_] revealed the neutral [P_1_] species **12** showing a four‐membered ring with a single phosphorus atom connected to an imidazoliumyl‐moiety and a Ga_2_Cl_5_‐fragment (Figure 4).

Furthermore, the ^31^P{^1^H} NMR spectrum of the reaction mixture displayed resonances (*δ*(P_X_)  =  ‐197.3 ppm, *δ*(P_A_)  =  ‐316.9 ppm, ^1^ *J*(P_A_P_X_)  =  −168.5 Hz), which correspond to the bicyclo[1.1.0]tetraphosphane dication **13** ^2+^.^[^ ^14^ ^]^ Its formation is likely initiated by a one‐electron oxidation of **11**, facilitated by the gallium derivatives present in the reaction. This step would generate a transient phosphinidene species, whose follow‐up chemistry—including dimerization—has been demonstrated in early work by Fritz,^[^ ^21^ ^]^ and in related low‐valent P₄ systems by Frank.^[^ ^22^ ^]^ Bertrand and co‐workers have also contributed to understanding the reactivity of phosphinidenes in condensed phases.^[^ ^23^ ^]^ Although not directly related to the structure of **13** ^2^⁺, these precedents support the feasibility of such dimerization steps. Various synthetic strategies toward bicyclo[1.1.0]tetraphosphanes (“P₄ butterfly” structures) have been summarized in a recent minireview by Scalambra and Romerosa.^[^ ^24^ ^]^

### 2.1 Protonation of 10[Ga_2_Cl_7_] versus Nucleophile‐Induced Ring‐Closure

**Scheme 5:**
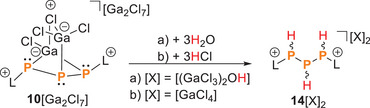
Protonation of **10**[Ga_2_Cl_7_] with 3 eq. of BRØNSTED acids H_2_O or HCl.

**Figure 5:**
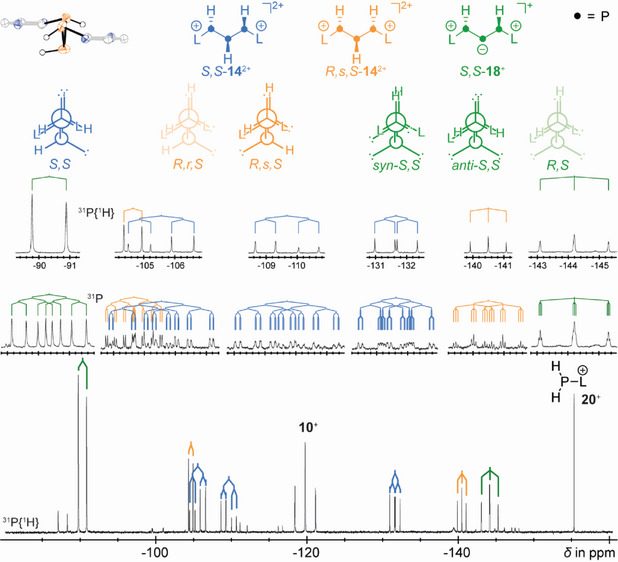
^31^P{^1^H} NMR spectrum of the reaction mixture of **10**[Ga_2_Cl_7_] and one equivalent of water (bottom); the resonance at *δ * =  −119.5 ppm belongs to the starting material and that at *δ * =  −155.5 ppm is assigned to cation **20** ^+^;^[^ ^26^ ^]^ extended signals and splitting diagrams of the product species in the ^31^P and ^31^P{^1^H} NMR spectra (middle); potential (faded) and actual (highlighted) isomers and conformers of the reaction products (top); section of the crystal structure of *rac*‐**14** ^2+^ (top, left); selected atoms and counter ions are omitted for clarity; ellipsoids are drawn at 50 % probability level.

Next, we investigated whether the cation **10^+^** could be protonated similar to **6** ^+^. Treatment of **10**[Ga_2_Cl_7_] with three equivalents of brønsted acids, such as water or HCl, induced a stepwise protonation reaction that cleaved all Ga–P bonds, ultimately yielding the dication **14** ^2+^, in which all phosphorus atoms carry one hydrogen atom (Scheme 5, Figure 5). This reaction proceeds in a stoichiometric manner, and the composition of the product, particularly the anion, was confirmed by X‐ray diffraction on single crystals obtained from the hydrolysis reaction solution (vide infra). The dication **14** ^2+^ represents a rare example of a *catena*‐triphosphane. The parent compound P_3_H_5_ is highly reactive and tends to disproportionate into PH_3_ and higher polyphosphanes, especially when heated or under irradiation.^[^ ^25^ ^]^

Detailed ^31^P NMR studies identified two out of three possible diastereomers, that is, R*,R*‐**14** ^2+^ and *R*,*s*,*S*‐**14** ^2+^ (Figure 5). The *R*,*s*,*S*‐**14** ^2+^ diastereomer shows only two resonances in the ^31^P{^1^H} NMR spectrum, while the racemic R*,R*‐**14** ^2+^ gives an independent singlet. R*,R*‐**14** ^2+^ crystallized as a racemate, consisting of the *R*,*R*‐**14** ^2+^ and *S*,*S*‐**14** ^2+^ isomers in a single crystal. R*,R*‐**14** ^2+^ gives rise to the splitting pattern highlighted in blue. The large ^2^ *J*(PP) coupling constant of 282 Hz is consistent with the parallel alignment of the lone pairs of the outer phosphorus atoms, consistent with the solid‐state structure of this cation (vide infra). The signals attributed to *R,s*,*S*‐**14** ^2+^ display a smaller ^1^ *J*(PP) coupling constant of −118 Hz. This excludes the assignment to *R*,*r*,*S*‐**14** ^2+^, as the lone pairs are aligned parallel in the *R* and *r* configurations.

This assignment is supported by ^31^P NMR data for related triphosphanes reported in the literature (Figure 7 and Table S1). For *R*,*r*,*S*‐**15**, the lone isomer of 1,3‐diaminotriphosphane obtained from the reaction of sodium and P_4_ in liquid ammonia, has a large ^1^ *J*(PP) coupling constant of ‐238 Hz.^[^ ^27^ ^]^ No configuration was assigned to the ditungsten complex of tBu_2_P_3_H_3_, [{W(CO)_5_}_2_(**16**)], which exhibits a fairly large ^1^ *J*(PP) coupling constant of ‐197 Hz as well.^[^ ^28^ ^]^

Table S1 lists the shifts and coupling constants of P_3_H_5_ (**17**)^[^ ^29^ ^]^ and its ruthenium complex [{RuCp(PPh_3_)_2_}_2_(**17**)].^[^ ^30^ ^]^ An intermediate bearing an A_2_X spin system with only two hydrogen atoms was also identified in the reaction mixture by ^31^P NMR spectroscopy. This species is thought to be the *rac*‐**18** ^+^ monocation (Figure 5, top right), related to the linear polyphosphide LiP_3_H_4_ (see Table 1),^[^ ^31^ ^]^ although it cannot be ruled out that the central phosphorus atom may still carry a gallium fragment. The assignment to a hypothetical *meso* compound *R*,*S*‐**18** ^+^ is ruled out due to the observed large ^3^ *J*(PH) coupling constant of 95 Hz. A systematic study of the magnitude of the ^3^ *J*(PH) coupling was conducted by hersh et al.,^[^ ^32^ ^]^ following the well‐known karplus relation for ^3^ *J*(HH) coupling constants.^[^ ^33^ ^]^ They found that the minima occur when the torsion angle between the phosphorus lone pair and the hydrogen atom at 1,3‐distance is 90°, with maxima at 0° or 180°. The assignment of the products to either the *syn‐* or *anti‐*conformer remains unclear at this stage. One clue is the relatively large ^1^ *J*(PP) coupling constant of ‐223 Hz. However, the presence of gallium may influence this value. Diffraction analysis or ^1^H NMR spectroscopy of the pure compound could clarify this issue, but this was beyond the scope of this study.

**Table 1:** Characterization of selected bond critical points and DI(A,B) values in compound **25** ^+^ (all values in au.).

BCP	∇^2^ρ(*r*_c_)	G(*r*_c_)/ ρ(*r*_c_)	H(*r*_c_)	DI(A,B)
Pd–P2	0.0848	0.6156	−0.0332	0.8144
Pd–P3	0.0910	0.6250	−0.0293	0.7938
Pd–P4	0.1690	0.7447	−0.0336	0.8180
Pd–P5	0.1508	0.7526	−0.0345	0.8253
P1–P2	−0.0939	0.3233	−0.0589	1.0406
P1–P3	−0.0949	0.3249	−0.0595	1.0657
P2–P3	−0.1172	0.3520	−0.0721	1.2222

**Figure 6:**
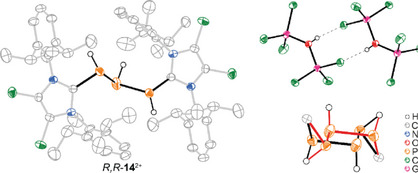
Molecular structure of dication *R,R*‐**14** ^2+^ (left) and of the dimer of the anion [(GaCl_3_)_2_OH]^−^ (top, right); the disorder of the two enantiomeric C_2_P_3_H_3_ sections is depicted in red (*S,S*‐**14** ^2+^) and black (*R,R*‐**14** ^2+^; bottom, right); hydrogen atoms and disorder are omitted for clarity; ellipsoids are drawn at 50% probability level.

Crystals of racemic *R*,*S*‐**14**[(GaCl_3_)_2_OH]_2_ were obtained by slow diffusion of *n*‐pentane into the reaction solution of **10**[Ga_2_Cl_7_] and water (1:3). The salt crystallized in the centrosymmetric space group *P *− 1. Both enantiomers were disordered on the same site (see Figure 6, bottom right). The hydrogen atoms bonded to phosphorus were refined using the electron density maxima in the difference Fourier map, and the P–H bond lengths were restrained to be similar. The P–P and P–C bond lengths and the P1–P2–P3 bond angle did not differ significantly from those in the starting material **10**[Ga_2_Cl_7_].

**Figure 7:**
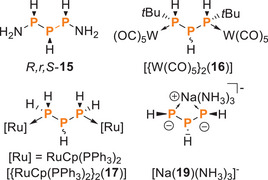
Compounds possessing a P_3_H_3_ structural unit.

The angle (93.85(5)°) is, however, smaller than in related P_3_H_3_ compounds, such as [{W(CO)_5_}_2_(**16**)]^[^ ^28^ ^]^ and [Na(NH_3_)_5_][Na(**19**) (NH_3_)_3_]^[^ ^34^ ^]^ (Figure 7; Table 1). The counterion represents an OH^−^ moiety coordinated to two GaCl_3_ fragments. A similar anion was previously described in compound [InI(18c6)][(GaCl_3_)_2_OH], were it forms a hydrogen bridge to the crown ether.^[^ ^35^ ^]^ In *rac*‐**14**[(GaCl_3_)_2_OH]_2_, the anions form hydrogen‐bridging dimers across the inversion centre, involving two O–H···Cl bridges (Figure 6; top right). Similar results were obtained when **10**[Ga_2_Cl_7_] was treated with 1 M HCl solution in Et_2_O, where the counteranions were suggested to be [GaCl_4_]^−^ (Scheme5 and Figure S9 for the ^31^P{^1^H} NMR spectrum of the reaction mixture). In all cases, however, the isolation of the respective salts proved challenging due to the complexity of the reaction mixture. Additionally, these triphosphanes exhibited decomposition phenomena, as evidenced by the formation of the cationic primary phosphane [LPH_2_]^+^ (**20** ^+^)^[^ ^26^ ^]^ in the reaction mixture (see Figure 5).

**Figure 8:**
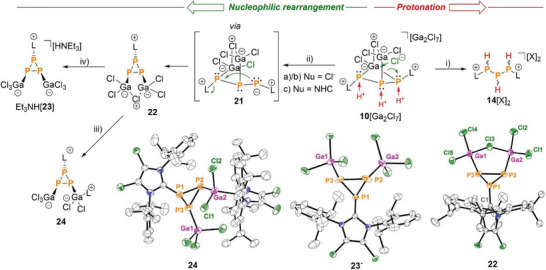
(top) Proposed reaction of **10**[Ga_2_Cl_7_] with brønsted acids (protonation) to cations **14**[X]_2_, nucleophile‐induced ring closure to cyclo‐triphosphirane‐2,3‐diide **21** and subsequent nucleophilic attack to yield either Et_3_NH[**23**] or **24**; i) a) + 3 eq. H_2_O, rt, CH_2_Cl_2_, [X]^−^ = [(GaCl_3_)_2_OH]^−^, b) + 3 eq. HCl, rt, CH_2_Cl_2_, [X]^−^ = [GaCl_4_]^−^; ii) Nu = Cl^−^: a) + 1 eq. HDMAP[Cl], ‐LGaCl_3_, ‐HDMAP[Ga_2_Cl_7_], CH_2_Cl_2_, rt; b) + Et_4_N[Cl], ‐LGaCl_3_, – Et_4_N[Ga_2_Cl_7_], CH_2_Cl_2_, rt, 16%; Nu = NHC L: c) + NHC, – 2 LGaCl_3_, – GaCl_3_, CH_2_Cl_2_, rt; iii) + NHC, CH_2_Cl_2_, rt; iv): from **10**[Ga_2_Cl_7_] in one step: + 3 Et_3_NH[Cl], – 2 Et_3_NH [GaCl_4_], CH_2_Cl_2_, rt, 20%; (bottom) molecular structures of **22, 23** ^+^ in Et_3_NH[**23**]**·**C_6_H_5_F and **24**, hydrogen atoms, anions, and solvate molecules are omitted for clarity and thermal ellipsoids are displayed at 50% probability; selected bond lengths [Å] and angles [°]: **22**: P1‐P2: 2.1937(13), P2‐P3: 2.2158(15), P1‐P3: 2.2005(15), P2‐Ga2: 2.3479(12), P3‐Ga1: 2.3351(12), P1‐C1: 1.855(4), P1‐P2‐Ga2: 91.12(5), P1‐P3‐Ga1: 97.32(5); **23** ^−^: P1‐P2: 2.1791(9), P2‐P3: 2.1959(13), P1‐P3: 2.1690(9), P2‐Ga1: 2.3464(9), P1‐C: 1.855(2), P1‐P2‐Ga1: 100.52(3); **24**: P1‐P2 2.196(3), P1‐P3 2.185(2), P2‐P3 2.213(2), P2‐Ga2 2.3603(18), P3‐Ga1 2.3469(16), Ga2‐Cl1 2.1970(17), Ga2‐Cl2 2.176(2).

In further studies, we treated compound **10**[Ga_2_Cl_7_] with one equivalent of HDMAP[Cl] (DMAP = 4‐dimethylaminopyridine) as a mild proton source. Unlike the reaction with HCl, the known *catena* species **14** ^2+^ was not formed (Figure 8, iia).

The reaction mixture exhibited a new product with a characteristic A_2_X spin system in the ^1^H‐coupled and decoupled ^31^P NMR spectra (*δ*(P_A_) = ‐238.6 ppm, *δ*(P_X_) = ‐158.8 ppm, ^1^ *J*(P_A_P_X_) = ‐175 Hz), typical for three‐membered saturated P_3_‐ring systems.^[^ ^36^, ^37^ ^]^ The absence of any ^1^ *J*_PH_ couplings indicates that the reaction did not proceed via protonation, but was driven by the presence of Cl^−^ counterions. To confirm this assumption, we repeated the same reaction with one equivalent of Et_4_N[Cl] as an alternative Cl^−^ source. After two hours, the reaction NMR displayed the same A_2_X spin pattern as observed with HDMAP[Cl], confirming that Cl^−^ initiated a form of ring closure (Figure 8 iib).

Both reactions were worked up, revealing the formation of the LGaCl_3_ adducts, HDMAP[Ga_2_Cl_7_] (in the reaction with HDMAP[Cl]) and Et_4_N[GaCl_4_] (in the reaction with Et_4_N[Cl]), which were identified by X‐ray analysis. The reactions proceed relatively rapidly, and our studies indicate that product formation is completed within two hours. However, the product was not stable over time, transforming into new species. We isolated the corresponding product in small amounts by removing all volatiles and washing the formed LGaCl_3_ adduct with benzene, followed by fluorobenzene. Recrystallization from CH_2_Cl_2_ by vapor diffusion of *n*‐pentane at ‐35 °C yielded pale yellow blocks suitable for X‐ray analysis from the Et_4_N[Cl] reaction, after removing a first batch of crystalline Et_4_N[GaCl_4_]. The obtained crystals were found to be a mixture of two compounds, both featuring a cyclic P_3_‐core with one imidazolium fragment bound to one phosphorus atom. The other two phosphorus atoms in the three‐membered ring each carry a GaCl_2_ fragment. In compound **22**, this is bridged by an additional chloride, while in compound **22′**, it is bridged by an OH group, forming a bicyclic structure (see Figure S13 and S17). Both compounds are neutral, with the positive charge of the imidazolium fragment being compensated by the bridging Cl^−^ or OH^−^, respectively. Compound **22′** resulted from the hydrolysis of **22**, as we observed that this compound was highly sensitive to moisture and readily transformed during work‐up.

Both compounds **22** and **22′** crystallized in the space group *P*2_1_/c. The [P_3_[(GaCl_2_)_2_OH]] moiety in **22′** is disordered, so only average bond lengths and angles are discussed. The P–P bonds within the P_3_ moiety in **22** (P1–P3: 2.2005(15) Å, P1–P2: 2.1937(13) Å, P2–P3: 2.2158(15) Å) and **22′** (average 2.200 Å) are comparable to related three‐membered P_3_ derivatives, such as Dipp_3_P_3_ (Dipp = 2,6‐diisopropylphenyl) (P1–P2: 2.1991(4) Å, P1–P3: 2.2440(4) Å, P2–P3: 2.2124(3) Å) or (L_C_)_3_P[OTf]_3_ (L_C_ = 4,5‐dimethyl‐1,3‐diisopropylimidazol‐2‐yl) (P1–P2: 2.2459(6) Å, P1–P3: 2.2139(6) Å, P2–P3: 2.2293(6) Å).^[^ ^36^, ^37^ ^]^ The P–Ga bond lengths in **22** and **22′** (average 2.34 Å) are typical for Ga–Cl complexes.^[^ ^38^ ^]^ Within the (GaCl_2_)_2_Cl fragment, the Ga–Cl bond lengths vary depending on the coordination mode, with 2.3205(12) Å and 2.3062(11) Å toward the bridging Cl^−^ and an average 2.1618 Å for the terminal Ga–Cl bonds. Comparable values are also observed in **10** ^+^ (e.g., Ga1–Cl1_term_: 2.134(2) Å, Ga2–Cl2_bridge_: 2.366(19) Å). The P–P–Ga fold angles in **22** (97.32(5)° and 91.12(5)°) are narrower than those in related derivatives, and the imidazolium substituent and the [(GaCl_2_)_2_X] fragment (X = Cl, OH) are in *anti*‐configuration.

**Scheme 6:**
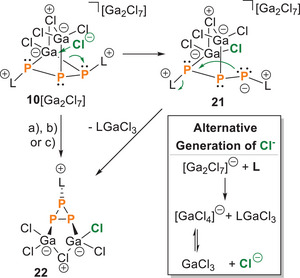
Mechanism of the formation of **22** through the chloride‐induced rearrangement of **10^+^**. Reaction of **10**[Ga_2_Cl_7_] with 1 eq. of NHC **L** to yields compound **22** through in‐situ generation of chloride from the [Ga_2_Cl_7_]^−^ counterion. a) + 1 eq. HDMAP[Cl], ‐LGaCl_3_, ‐HDMAP[Ga_2_Cl_7_], CH_2_Cl_2_, rt; b) + Et_4_N[Cl], ‐LGaCl_3_, – Et_4_N[Ga_2_Cl_7_], CH_2_Cl_2_, rt, 16 %; c)+L, – 2 LGaCl_3_, rt, CH_2_Cl_2_.

Coherent with the decreased bond lengths between Ga and the bridging oxygen atom (average 1.9401 Å) compared to the bridging Ga–Cl bonds, the Ga–(OH)–Ga angle in **22′** (average 113.61°) is significantly wider than the Ga–Cl–Ga angle (99.78(4)°) in **22**. In **22′**, the Ga–(OH) bond lengths are slightly elongated (average 1.94 Å) compared to the Ga–(OH)–Ga moieties in compound {Fe(CO)_4_}_2_Ga_4_Cl_5_(OH)_3_(THF), which has a Ga_4_Fe_2_O_4_ core (1.877(2) Å‐1.903(2) Å).^[^ ^39^ ^]^ The [(GaCl_2_)_2_Cl] fragment, as observed in **22**, was first reported by schulz and co‐workers^[^ ^40^ ^]^ and has also been observed in compound **10^+^** (vide supra). In this reaction, a different process occurs compared to the protonation of the phosphorus atoms in cation **10^+^**. Here, the chloride ion likely attacks the bridging Ga atom of the four‐membered P_3_Ga‐ring, leading to the cleavage of one of the P–Ga bonds and the generation of an exocyclic phosphanidic phosphorus atom, forming intermediate **21** (Scheme 6). This phosphanidic P atom subsequently intramolecularly attacks the P atom carrying the second imidazolium substituent (**L**), leading to the formation of a P_3_‐ring and the release of the imidazolium substituent as free NHC **L**. The free NHC **L** then reacts with the counteranion [Ga_2_Cl_7_]^−^ to form an LGaCl_3_ adduct and the [GaCl_4_]^−^ anion (Scheme 6, box). Our research also demonstrated that Cl could be replaced by OH in compound **22** (see Supporting Information, Figure S14), offering further opportunities for new bonding motifs. Additionally, we also found that the carbene **L** released during the reaction is trapped by GaCl_3_ and is therefore unavailable for further reactions. Given that the [Ga_2_Cl_7_]^−^ anion is essentially a masked Cl^−^ source coordinated by two GaCl_3_ units, we hypothesized that in the presence of a nucleophilic free **L** in the correct stoichiometric ratio, this anion would liberate Cl^−^, leading to the formation of compound **22**.

**Scheme 7:**
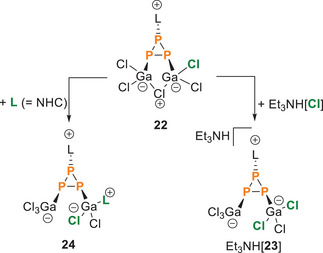
Nucleophilic opening of **22** with NHC **L** to yield **24** or with Et_3_NH[Cl] to yield Et_3_NH[**23**].

This approach avoids the formation of salts observed in the reaction with HDMAP[Cl] and Et_4_N[Cl], yielding only two equivalents of the LGaCl_3_ adduct (Scheme 6). This is due to the tetrachlorogallate being in equilibrium with GaCl_3_ and Cl^−^ in solution.^[^ ^41^ ^]^ However, the isolation of compound **22** synthesized under these conditions is again hindered by its extreme sensitivity toward nucleophiles, particularly traces of moisture. The selective addition of nucleophiles to **22** leads to the opening of the Ga–Cl–Ga bridge to afford the respective two‐coordinate triphosphiranes. For example, the addition of one equivalent of NHC **L** to **22** affords compound **24** (Figure 8 and Scheme 7; for experimental details see Supporting Information Section 2). Interestingly, similar results were observed when compound **10**[Ga_2_Cl_7_] was reacted with a twofold excess of **L**, forming **22** in‐situ. The isolation of **24** was hampered due to its low stability and the concurrently formed **L**GaCl_3_ adduct, which proved inseparable from **24**. However, we were able to isolate a small amount of compound **24** through recrystallization to allow for further analysis. When dissolved in CH_2_Cl_2_ compound **24** exhibits the expected ABX spin system in its ^31^P NMR spectrum (*δ*(P_A_) = −212.6 ppm, *δ*(P_B_) = −206.1 ppm, *δ*(P_X_) = −133.1 ppm, ^1^ *J*(P_A_P_B_) = −251 Hz, ^1^ *J*(P_A_P_X_)  = −186 Hz, ^1^ *J*(P_B_P_X_)  = −189 Hz). Crystals suitable for structural analysis were obtained via fractional crystallization. The crystal structure is chiral, belonging to the *P*2_1_2_1_2_1_ space group, suggesting that crystallization proceeded by spontaneous resolution, resulting in a racemic conglomerate. However, only one crystal was measured by X‐ray diffraction, yielding a Flack^[^ ^2^ ^]^ parameter x of 0.009(8). The molecular structure of compound **24** aligns with the binding parameters of the LP_3_ backbone in compounds **22** and **22′** (e.g., P2‐P3 bond lengths in Å: **24**: 2.213(2), **22**: 2.2158(15), **22′** 2.219(3)). As anticipated, the structure confirms the opening of the [(GaCl_2_)_2_Cl] fragment and reveals the *cis* arrangement of the attached GaCl_3_ with respect to the GaCl_2_ **L** group, expected from the nucleophilic opening of **22** by **L**. Additionally, the latter is oriented *trans* relative to the imidazoliumyl substituents directly attached to the P_3_‐ring, likely due to the larger spatial requirement of the **L** substituents.

The nucleophilic opening of compound **22** by a chloride source was also tested. Compound **22** was prepared in situ by reacting **10**[Ga_2_Cl_7_] with one equivalent of Et_4_N[Cl], followed by treatment with one equivalent of Et_3_NH[Cl] to facilitate crystallization and the removal of the by‐product LH[GaCl_4_] (see the Supporting Information for details). After three hours, the ^31^P NMR spectrum revealed a new AX_2_ spin system (*δ*(P_A_) = −215.2 ppm, *δ*(P_X_) = −139.9 ppm, ^1^ *J*(P_A_P_X_) = −186 Hz), but we were unable to separate or crystallize the compound from the reaction mixture. However, this reaction can also be carried out starting directly from **10**[Ga_2_Cl_7_] using three equivalents of Et_3_NH[Cl] or one equivalent of Et_3_NH[Cl] and two equivalents of Et_4_N[Cl] (Figure 8, reaction iv). In both cases, an immediate color change from pale yellow to deep red is observed, and after two hours, the same A_2_X spin system is observed in the respective ^31^P NMR spectrum. A series of salts, such as protonated NHC **L** ([H**L**][GaCl_4_]), Et_3_NH[GaCl_4_], Et_4_N[GaCl_4_], and the free base Et_3_N, were identified as by‐products. The excess protons lead to the protonation of the formed **L**GaCl_3_ adduct. After several attempts, we succeeded in isolating the new compound **23** ^−^ as the Et_3_NH‐salt by slowly diffusing *n*‐pentane into a saturated CH_2_Cl_2_ solution of the crude material, resulting in a 20 % yield. The crude material was obtained by removing all volatiles from the reaction mixture in vacuo and washing the solid material with *o*‐difluorobenzene. Fractional crystallization from the fluorobenzene solution using *n*‐pentane allowed us to separate Et_3_NH[GaCl_4_] (for further details, see the Supporting Information). Compound Et_3_NH[**23**] crystallizes in the space group *P2*_1_/c, with one molecule of fluorobenzene in the asymmetric unit. The P–P bond lengths within the P_3_ moiety (P1–P2: 2.1791(9) Å, P1‐P3: 2.1690(9) Å, P2–P3: 2.1959(13) Å) are significantly shortened compared to those in other triphosphirane derivatives, such as [(L_C_)_3_P_3_][OTf]_3_ (average 2.230 Å)^[^ ^37^ ^]^ or Dipp_3_P_3_ (average 2.225 Å).^[^ ^36^ ^]^ This shortening can be attributed to negative hyperconjugation between the lone pair electrons on the P1 atom and the P–Ga σ* orbitals, which are in an eclipsed configuration.^[^ ^42^ ^]^ This hyperconjugation leads to an elongation of the P–Ga bond and the observed reduction in P–P bond lengths. The P–Ga bond lengths could not be reliably determined due to the disorder of the GaCl_3_ units in the molecular structure of **23** ^−^.

**Scheme 8:**
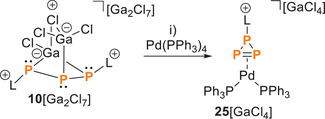
Reaction of **10**[Ga_2_Cl_7_] with Pd(PPh_3_)_4_. i)‐ L–GaCl_3_, – 2 PPh_3_, – Ga[GaCl_4_], CH_2_Cl_2_, rt, 5 minutes, 40 %.

**Scheme 9:**
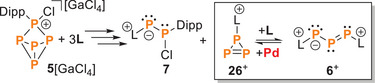
Proposed mechanism for the Pd(0)‐initiated back reaction of **6** ^+^ to **26** ^+^.

Finally, we sought to gain some initial insight into the coordination behavior of **10**[Ga_2_Cl_7_] toward transition metals. Treatment of **10**[Ga_2_Cl_7_] with one equivalent of Pd(PPh_3_)_4_ in CH_2_Cl_2_ results in a ring closure to form an **L**P_3_‐core, similar to the reactions of **10**[Ga_2_Cl_7_] with nucleophiles. This process leads to the formation of the triphosphirene complexes **25**[GaCl_4_], accompanied by the simultaneous release of PPh_3_ and the formation of various by‐products, including [H**L**][GaCl_4_], **L**GaCl_3_, and Ph_3_PGaCl_3_. The protonated by‐products likely arise from solvent activation by the liberated lewis acid GaCl_3_, which also explains the formation of various adducts. The overall reaction is depicted in Scheme 8 and proceeds rapidly, with a color change of the reaction solution occurring within 5 minutes. The final product, **25**[GaCl_4_], was isolated after a series of purification steps. First, all volatiles were removed in vacuo, and the residue was extracted with fluorobenzene. Addition of *n*‐pentane to the extract resulted in a brownish oil, which was then recrystallized from dichloromethane and *n*‐pentane at ‐35 °C, affording crystalline **25**[GaCl_4_] in 40% yield, suitable for further structure analysis. This reaction does not involve a redox process, instead, the Pd(0) initiates the liberation of Ga(I) and the formation of the triphosphirene [LP_3_]^+^ (**26** ^+^) according to Scheme 9, representing the reverse of the key step of our reported [3 + 2] fragmentation of compound **5**[GaCl_4_] with three equivalents of the NHC **L**.^[^ ^11^ ^]^ The ^31^P{^1^H} NMR spectrum of dissolved **25**[GaCl_4_] in CD_2_Cl_2_ exhibits an AM_2_X_2_ spin system. The two groups of signals resonating at high field (*δ*(P_A_) = −182.3 ppm, *δ*(P_M_) = −88.0 ppm) are assigned to the **L**P_3_‐ligand. The chemical shift for P_M_ and the coupling constant (^1^ *J*(P_A_P_M_) = −217 Hz) are consistent with those observed for uncoordinated triphosphiranes such as *t*Bu_3_P_3_,^[^ ^43^ ^]^ with *δ*(P_M_) = −71.3 ppm and ^1^ *J*(P_A_P_M_) = −201 Hz. However, the resonance of the P_A_ nucleus is observed at a much higher field compared to *t*Bu_3_P_3_ (*δ*(P_A_) = −109.6 ppm) due to the electron‐withdrawing effect of the imidazoliumyl substituent. This observation is consistent with our previously reported [(L_C_)_3_P_3_][OTf]_3_ (L_C_ = 1,3‐bis(2,6‐diisopropylphenyl)‐imidazol‐2‐yl), which exhibits *δ*(P_A_) = −142.5 ppm and *δ*(P_X_) = −130.8 ppm.^[^ ^37^ ^]^ The resonance for the PPh_3_‐phosphorus atoms (*δ*(P_X_) = 14.3 ppm) is slightly shifted to a higher field compared to Pd(PPh_3_)_4_ (*δ*(P) = 29.8 ppm).^[^ ^44^ ^]^

**Figure 9:**
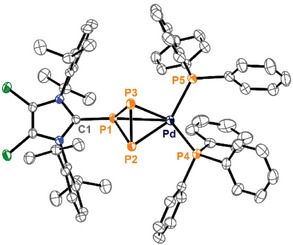
Molecular structure of **25** ^+^ in **25**[GaCl_4_]**·**CH_2_Cl_2_, hydrogen atoms are omitted for clarity and thermal ellipsoids are displayed at 50% probability; selected bond lengths [Å]: P1‐P2: 2.2033(8), P2‐P3: 2.1319(8), P1‐P3: 2.2053(8), P1‐Pd: 2.9269(6), Pd‐P2: 2.3874(6), Pd‐P3: 2.3781(6), P1‐C: 1.861(2), Pd‐P4: 2.3323(5), Pd‐P5: 2.3557(5).

Single crystals suitable for X‐ray analysis exhibit slight disorder, but the well‐resolved Pd‐η^2^‐complex **25** ^+^ (sight occupancy 97%) was the main component (Scheme 9). The P–C bond length is 1.861(2) Å, slightly longer compared to **10** ^+^ (1.835(4) Å). The Pd–P2 (2.3874(6) Å) and Pd–P3 (2.3781(6) Å) bonds are slightly longer compared to the Pd–PPh_3_ bonds (2.3323(5) and 2.3557(6) Å), similar to related (PPh_3_)_2_Pd‐diphosphene complexes such as (η^2^‐F_3_C–P = P–CF_3_)Pd(PPh_3_)_2_ (Pd‐PPh_3_: average 2.35 Å, Pd–P: average 2.36 Å) or (η^2^‐Mes–P = P–Mes)Pd(PBu_3_)_2_ (Pd–PBu_3_: average 2.33 Å, Pd–P: average 2.38 Å; Mes = 1,3,5‐trimethylphenyl).^[^ ^45^, ^46^ ^]^ The P1–P2 (2.2013(16) Å) and P1–P3 (2.2057(16) Å) bond lengths fall within the range for P–P single bonds. In contrast to the triphosphiranes [HNEt_3_][**23**] and **22**, the P2–P3 bond length in the complex **25** ^+^ is shortened (2.1216(17) Å), consistent with the P–P bond lengths in diphosphene complexes such as [(η^2^‐L_C_–P = P–L_C_)Pd(PPh_3_)_2_][OTf]_2_ (L_C_ = 4,5‐dimethyl‐1,3‐diisopropyl‐imidazol‐2‐yl) (2.1340(12) Å), (η^2^‐F_3_C–P = P–CF_3_)Pd(PPh_3_)_2_ (2.121 Å), and (η^2^‐Mes–P = P–Mes)Pd(PBu_3_)_2_ (2.1355(9) Å).^[^ ^37^, ^45^, ^46^ ^]^ These results support the characterization of the LP_3_ moiety in **25** ^+^ as a triphosphirene ligand (Figure 9).

**Figure 10:**
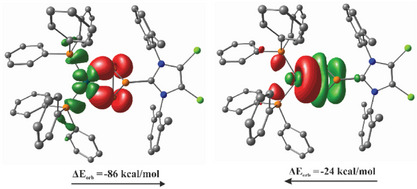
Contour plots of complementary NOCVs of complex **25** ^+^ (surface isovalue = 0.04) showing donor (green) and acceptor (red) together with the corresponding orbital interaction energies. Arrows depict the flux of electrons. Hydrogen atoms are omitted for clarity.

**Figure 11:**
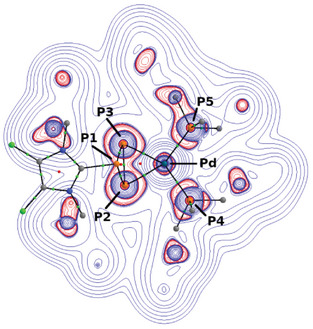
Visualization of ∇^2^ρ(r) (P2–P3–Pd plane, green points: BCP, red points: RCP) and the molecular graph of compound **25^+^** with atom numbering. Charge accumulations (∇^2^ρ(r) < 0) are printed in red, charge depletion (∇^2^ρ(r) > 0) in blue. 2,6‐Dimethylphenyl and phenyl moieties were omitted for clarity.

A related triphosphirene, Mes*P_3_, undergoes dimerization to form a tricyclic hexaphosphane, but was trapped as a cycloaddition product in the presence of excess cyclohexadiene.^47^ However, in the case of **25** ^+^, the charge repulsion and stabilizing effect of the Pd(PPh_3_)_2_ unit prevent this less favorable dimerization pathway. To gain a more detailed understanding of the bonding in cation **25** ^+^, we conducted a DFT study^[^ ^48^ ^]^ using the PBE0^[^ ^49^ ^]^‐D3BJ^[^ ^50^ ^]^/def2‐TZVP^[^ ^51^ ^]^ level of theory. In this model, we simplified the system by replacing the isopropyl groups in cation **25** ^+^ with methyl groups. The coordination of the Pd atom by the triphosphirene moiety follows the Dewar‐Chatt‐Duncanson (DCD) model, which is typically used to describe bonding in alkene‐metal π‐complexes. These bonding interactions involve both σ‐donation and π‐back‐donation and have been previously discussed in related Ni(0) and Pd(0) diphosphoniodiphosphirane complexes based on molecular orbitals.^[^ ^52^ ^]^ The molecular orbital analysis of cation **25** ^+^ revealed four doubly occupied d‐orbitals on the Pd center, along with two σ‐type Pd–P bonds to the P_3_‐ring. The P2–P3 bond displays typical single‐bond σ‐symmetry. This observation supports the formation of a metallacyclopropane‐like structure due to strong metal‐to‐ligand π‐back‐bonding, as predicted by the DCD model (see Supporting Information for additional information). An energy decomposition analysis (EDA) was performed to quantify the interactions involved in the formation of the Pd–P bond in cation **25** ^+^. In this analysis, the system was split into two fragments: [(PPh_3_)_2_Pd] and [NHC‐P_3_]^+^. The resulting natural orbitals for chemical valence (NOCVs) indicate a dominant π‐type metal‐to‐ligand back‐bonding, with an orbital interaction energy of ‐86 kcal/mol (Figure 10). To further understand the electron density's topology and bonding interactions, we employed the atoms‐in‐molecules (AIM)^[^ ^53^ ^]^ approach. Bond critical points (BCPs) were identified for all Pd–P bonds, located in regions of local charge depletion (∇2ρ(*r*) > 0), with a magnitude of approximately 10^−1^ au. Typically, positive values of the Laplacian function for electron density typically suggest closed‐shell, dative interactions. However, in cation **25** ^+^, the values for the local electronic kinetic energy density G(*r*_c_)/ρ(*r*_c_) and negative energy densities H(*r*_c_) suggest a more covalent, shared bonding character, classifying **25** ^+^ as a borderline case between dative and covalent bonding. In comparison, the P–P bonds in the three‐membered cycle exhibit a higher degree of covalency, supported by the delocalization index DI(A,B), which offers a reasonable estimate of bond order (Figure 11).^[^ ^54^ ^]^

From the BCP and natural bonding orbitals (NBO) analysis, we observed the DI(A,B) value for the Pd–P bonds to be approximately 0.80 and a value of 1.22 for the P2–P3 atom pair, which suggests the retention of partial double bond character (Table 1). The charges derived from AIM and NBO calculations are generally consistent, except for P4 and P5, where AIM and NBO charges are + 1.85 au and + 0.99 au, respectively (Table 2). The central Pd atom, along with P2 and P3, shows minimal charge, while P1 exhibits slight positive charge. Interestingly, the wiberg bond index (WBI) for Pd–P bonds sums up to 1.66, indicating that the Pd–P bonds are fluctuating in strength (Table 2). This is further supported by DI(A,B) values being less than 1 and the mayer bond indices averaging 0.68 for the Pd‐P bonds. Restricted natural resonance theory (NRT) on compound **25^+^** gave many resonance structures (Figures S38 and S39, SI). Two of these resonance structures with a total weight of 33.4% suggest 3c/4e “hyperbonding” involving the triphosphirene P atoms, Pd and PPh_3_ ligands.^[^ ^55^ ^]^ In addition, two resonance structures, accounting for 25.4 % of the total weight, feature a P2–P3 double bond, consistent with the DI(P2,P3) value of 1.22, indicating a partial double bond character in this bond (see the Supporting Information for details).

**Table 2:** Atomic charges and sum of the Wiberg bond indices of selected atoms in cation **25^+^**.

Atom	q_AIM_/au	q_NBO_/au	WBI
Pd	−0.0305	0.0353	1.6613
P1	0.3789	0.1333	3.0765
P2	0.0466	−0.1071	3.0359
P3	0.0378	−0.0928	3.0196
P4	1.8468	0.9919	3.5744
P5	1.8546	0.9876	3.5373

Overall, our computational data on **25^+^** are consistent with a triphosphirene‐palladium complex with significant metal‐to‐ligand π‐back‐bonding in accord with the DCD model.

## 3 Conclusion

In conclusion, this work introduces the first imidazoliumyl‐substituted bicyclo[2.1.1]‐P₃ compound, **10**[Ga_2_Cl_7_], achieved through the selective reduction of a triphosphaallyl precursor using Ga(I)[Ga_2_Cl_7_]. The resulting Ga_2_Cl_4_‐bridged triphosphane framework opens up new avenues for phosphorus‐based reactivity, as demonstrated by its selective P–C bond cleavage reactions with various chloride sources, including Et_3_NH[Cl], Et_4_N[Cl], and HDMAP[Cl]. These reactions highlight the formation of novel Ga_x_Cl_y_‐substituted triphosphiranes, such as Et_3_NH[**23**] and **24**, through a rare ring closure of the P_3_ moiety. Furthermore, the reaction with [Pd(PPh_3_)_4_] produced the exceptional palladium complex **25**[GaCl_4_], featuring a bicyclic P_3_Pd moiety, which has been structurally characterized.

DFT calculations provide significant insights into the bonding of **25**[GaCl_4_], revealing a strong π‐back‐donation from the metal center to the triphosphirene ligand, along with evidence of a 3‐center‐,4‐electron hyperbonding interaction within the P_3_Pd system. The detailed computational analysis supports the presence of unusual bonding phenomena, such as “long bonding,” in the phosphorus framework. This study not only introduces new types of P_3_ compounds but also demonstrates their reactivity and coordination potential, which could have broader implications in the development of novel phosphorus‐containing ligands and coordination complexes.

Future work will focus on further exploration of the reactivity of these systems, including their use as building blocks for functional materials and potential applications in catalysis. Additionally, the observed metal‐ligand interactions in the P_3_Pd moiety pave the way for more in‐depth studies on the stabilization of phosphorus‐rich coordination environments in both synthetic and catalytic contexts.

## Author Contributions

J.F.‐R., J.F., F.H., J.J.W, and M.H. conducted the experiments and optimized the syntheses, isolations, and purifications. C.Z. and C.S. were responsible for chemical calculations. J.F., J.J.W., and F.H. were responsible for collecting X‐ray data and refinement. R.W., and J.J.W. conceived, oversaw, and directed the project. J.F., F.H., and J.J.W. prepared the initial draft of the paper. J.J.W. and R.W. acquired funding. All authors discussed and analyzed the results and contributed to the editing of the manuscript.

## Conflict of Interests

The authors declare no conflict of interest.

## Supporting information



Supporting Information

## Data Availability

The data that supprot the findings of this study are available in the Supporting Information of this article. CCDC Deposition numbers: 2 385 680 – 2 385 694, and 2 449 066 contain the supplementary crystallographic data for this paper. These data are provided free of charge by the joint Cambridge Crystallographic Data Centre and Fachinformationszentrum Karlsruhe Access Structures service.

## References

[1] M. Donath , F. Hennersdorf , J. J. Weigand , Chem. Soc. Rev. 2016, 45, 1145. 26853380 10.1039/c5cs00682a

[2] M. Yoshifuji , I. Shima , N. Inamoto , K. Hirotsu , T. Higuchi , J. Am. Chem. Soc. 1981, 103, 4587.

[3] Y. Wang , Y. Xie , P. Wei , R. B. King , H. F. Schaefer III , P. V. R. Schleyer , G. H. Robinson , Science 2008, 321, 1069. 18719279 10.1126/science.1160768

[4] Y. Wang , Y. Xie , P. Wei , R. B. King , H. F. Schaefer , P. v. R. Schleyer , G. H. Robinson , J. Am. Chem. Soc. 2008, 130, 14970. 18937460 10.1021/ja807828t

[5] M. Y. Abraham , Y. Wang , Y. Xie , P. Wei , H. F. Schaefer , P. V. R. Schleyer , G. H. Robinson , Chem. ‐ Eur. J. 2010, 16, 432. 19937872 10.1002/chem.200902840

[6] B. D. Ellis , C. L. Macdonald , Coord. Chem. Rev. 2007, 251, 936.

[7] a) Y. Wang , Y. Xie , P. Wei , H. F. Schaefer , P. v. R. Schleyer , G. H. Robinson , J. Am. Chem. Soc. 2013, 135, 19139; 24299493 10.1021/ja411667f

[8] A. Beil , R. J. Gilliard , H. Grützmacher , Dalton Trans. 2016, 45, 2044. 26400646 10.1039/c5dt03014e

[9] K. Schwedtmann , M. H. Holthausen , C. H. Sala , F. Hennersdorf , R. Fröhlich , J. J. Weigand , Chem. Commun. 2016, 52, 1409. 10.1039/c5cc08248j 26627185

[10] a) S. Yao , Tris(trimethylsilyl)silyl stabilized phosphorus and lead clusters , 2005;

[11] M. H. Holthausen , S. K. Surmiak , P. Jerabek , G. Frenking , J. J. Weigand , Angew. Chem., Int. Ed. 2013, 52, 11078. 10.1002/anie.201302914 24038818

[12] A. M. Tondreau , Z. Benkő , J. R. Harmer , H. Grützmacher , Chem. Sci. 2014, 5, 1545.

[13] W. Frank , W. Hönle , A. Simon , Z. Naturforsch. B. 1990, 45, 1.

[14] M. Holthausen , Syntheses and reactivity studies of cationic polyphosphorus cages , 2013. 10.1039/c4cs00019f PMC4288811 24740160

[15] J. Hahn , M. Baudler , C. Krüger , Y.‐H. Tsay , Z. Naturforsch. B. 1982, 37, 797.

[16] C. Frenzel , E. Hey‐Hawkins , Phosphorus Sulfur Silicon Relat. Elem. 1998, 143, 1.

[17] P. Pyykkö , M. Atsumi , Chem. Eur. J. 2009, 15, 186. 19058281 10.1002/chem.200800987

[18] J. Frötschel‐Rittmeyer , M. Holthausen , C. Friedmann , D. Röhner , I. Krossing , J. J. Weigand , Sci. Adv. 2022, 8, eabq8613. 36070385 10.1126/sciadv.abq8613 PMC9451154

[19] a) H. S. Gutowsky , C. H. Holm , J. Chem. Phys. 1956, 25, 1228;

[20] F. Hennersdorf , J. J. Weigand , Angew. Chem. Int. Ed. 2017, 56, 7858. 10.1002/anie.201703953 28475263

[21] G. Fritz , T. Vaahs , H. Fleischer , E. Matern , Angew. Chem. Int. Ed. Engl. 1989, 28, 315.

[22] V. Breuers , C. W. Lehmann , W. Frank , Chem. ‐ Eur. J. 2015, 21, 8812. 25703334 10.1002/chem.201406131

[23] M. M. Hansmann , R. Jazzar , G. Bertrand , J. Am. Chem. Soc. 2016, 138, 8356. 27340902 10.1021/jacs.6b04232

[24] F. Scalambra , A. Romerosa , Eur. J. Inorg. Chem. 2021, 291. 10.1021/acs.inorgchem.1c03831 PMC9019812 35378037

[25] a) T. P. Fehlner , J. Am. Chem. Soc. 1968, 90, 6062;

[26] M. Cicač‐Hudi , C. M. Feil , N. Birchall , M. Nieger , D. Gudat , Dalton Trans. 2020, 49, 17401. 33216079 10.1039/d0dt03633a

[27] M. Baudler , D. Düster , Z. Naturforsch. B. 1987, 42, 330.

[28] M. Scheer , C. Kuntz , M. Stubenhofer , M. Zabel , A. Y. Timoshkin , Angew. Chem., Int. Ed. 2010, 49, 188. 10.1002/anie.200904827 19957255

[29] P. Junkes , M. Baudler , J. Dobbers , D. Rackwitz , Z. Naturforsch. B. 1972, 27, 1451.

[30] P. Barbaro , C. Bazzicalupi , M. Peruzzini , S. Seniori Costantini , P. Stoppioni , Angew. Chem., Int. Ed. 2012, 51, 8628. 10.1002/anie.201203908 22810960

[31] M. Baudler , Angew. Chem., Int. Ed. 1982, 21, 492.

[32] W. H. Hersh , S. T. Lam , D. J. Moskovic , A. J. Panagiotakis , J. Org. Chem. 2012, 77, 4968. 22612503 10.1021/jo3003776 PMC3377502

[33] M. Karplus , J. Am. Chem. Soc. 1963, 85, 2870;

[34] N. Korber , J. Aschenbrenner , Dalton Trans. 2001, 1165.

[35] B. F. T. Cooper , H. Hamaed , W. W. Friedl , M. R. Stinchcombe , R. W. Schurko , C. L. B. Macdonald , Chem. Eur. J. 2011, 17, 6148. 21500291 10.1002/chem.201002946

[36] A. Schumann , F. Reiß , H. Jiao , J. Rabeah , J.‐E. Siewert , I. Krummenacher , H. Braunschweig , C. Hering‐Junghans , Chem. Sci. 2019, 10, 7859. 31853345 10.1039/c9sc02322d PMC6839504

[37] K. Schwedtmann , J. Haberstroh , S. Roediger , A. Bauzá , A. Frontera , F. Hennersdorf , J. J. Weigand , Chem. Sci. 2019, 10, 6868. 31391910 10.1039/c9sc01701a PMC6640194

[38] S. Herler , P. Mayer , J. S. auf der Günne , A. Schulz , A. Villinger , and J. J. Weigand , A. Triazaphosphole , Angew. Chem., Int. Ed. 2005, 44, 7790. 10.1002/anie.200502475 16259031

[39] T. Adamczyk , G.‐M. Li , G. Linti , H. Pritzkow , A. Seifert , T. Zessin , Eur. J. Inorg. Chem. 2011, 3480.

[40] A. Villinger , A. Westenkirchner , R. Wustrack , A. Schulz , Inorg. Chem. 2008, 47, 9140. 18803376 10.1021/ic801501c

[41] J. J. Weigand , N. Burford , A. Decken , Eur. J. Inorg. Chem. 2008, 4343.

[42] J. Joy , E. D. Jemmis , K. Vidya , Faraday Discuss. 2015, 177, 33. 25653178 10.1039/c4fd00183d

[43] M. Baudler , J. Hahn , H. Dietsch , G. Fürstenberg , Z. Naturforsch. B. 1976, 31, 1305.

[44] E. Ye , H. Tan , S. Li , W. Y. Fan , Angew. Chem., Int. Ed. 2006, 45, 1120. 10.1002/anie.200503408 16385592

[45] I. G. Phillips , R. G. Ball , R. G. Cavell , Inorg. Chem. 1992, 31, 1633.

[46] S. Gómez‐Ruiz , E. Hey‐Hawkins , Dalton Trans. 2007, 5678. 18060112 10.1039/b711616k

[47] J. E. Borger , A. W. Ehlers , M. Lutz , J. C. Slootweg , K. Lammertsma , Angew. Chem. Int. Ed. 2017, 56, 285. 10.1002/anie.201607234 27900815

[48] F. Neese , WIREs Comput. Mol. Sci. 2012, 2, 73.

[49] C. Adamo , V. Barone , J. Chem. Phys. 1999, 110, 6158.

[50] S. Grimme , S. Ehrlich , L. Goerigk , J. Comput. Chem. 2011, 32, 1456. 21370243 10.1002/jcc.21759

[51] F. Weigend , Phys. Chem. Chem. Phys. 2006, 8, 1057. 16633586 10.1039/b515623h

[52] S. C. Kosnik , J. F. Binder , M. C. Nascimento , A. Swidan , C. L. B. Macdonald , Chem. ‐ Eur. J. 2019, 25, 1208. 30468552 10.1002/chem.201805711

[53] R. F. W. Bader , Acc. Chem. Res. 1985, 18, 9.

[54] C. L. Firme , O. Antunes , P. M. Esteves , Chem. Phys. Lett. 2009, 468, 129.

[55] a) C. R. Landis , F. Weinhold , Inorg. Chem. 2013, 52, 5154; 23597392 10.1021/ic4000395

