# Supplementary material for: Synthesis and Reactivity of Triphosphaallyl Cation Stabilized by N‐Heterocyclic Carbenes
Source: Chemistry. 2025 Jun 4;31(37):e202501311. doi: 10.1002/chem.202501311 (PMC12223479; doi:10.1002/chem.202501311)
Supplement: Supplementary file 1 — Supporting Information [file CHEM-31-e202501311-s001.pdf]

Supporting Information

Synthesis and Reactivity of Triphosphaallyl Cation Stabilized by N-Heterocyclic Carbenes

Julia Frötschel-Rittmeyer,[a] Felix Hennersdorf,[a] Jannis Fidelius,[a] Chris Sala,[a] Christoph Ziegler,[b] Michael Holthausen,[a] Kai Schwedtmann,[a] Robert Wolf,[b] and Jan J. Weigand*,[a]

[a]Faculty of Chemistry and Food Chemistry, Technische Universität Dresden, 01062 Dresden, Germany

[b]Institute of Inorganic Chemistry, Universität Regensburg, 93040 Regensburg, Germany

[*] E-mail: jan.weigand@tu-dresden.de

Table of contents

1. General remarks, materials and methods 4

2. Synthetic details and characterization data 5

2.1. Reaction of $L_2P_3[GaCl_4]$ ($6[Ga_2Cl_7]$) with HOTf 5

2.2. Reaction of $6[GaCl_4]$ with $[PCl_4][GaCl_4]$ and $GaCl_3$ (1:1:2) 6

2.3. Synthesis of $L_2P_3(Ga_2Cl_4)[Ga_2Cl_7]$ ($10[Ga_2Cl_7]$) 7

2.4. Hydrolysis of $L_2P_3(Ga_2Cl_4)[Ga_2Cl_7]$ ($10[Ga_2Cl_7]$) with water 10

2.5. Hydrolysis of $L_2P_3(Ga_2Cl_4)[Ga_2Cl_7]$ ($10[Ga_2Cl_7]$) with HCl 11

2.6. Reaction of $10[Ga_2Cl_7]$ with HDMAP[Cl] 12

2.7. Synthesis of 22 by reaction of $10[Ga_2Cl_7]$ with $Et_4N[Cl]$ 13

2.8. Synthesis of $Et_3NH[LP_3(GaCl_3)_2]$ ($Et_3NH[23]$) by reaction of $10[Ga_2Cl_7]$ with $Et_3NH[Cl]$ 15

2.9. Reaction of $10[Ga_2Cl_7]$ with one equiv. of L 18

2.10. Reaction of$10[Ga_2Cl_7]$ with an excess of L 18

2.11. Reaction of $10[Ga_2Cl_7]$ with $NEt_4[Cl]$ and L 19

2.12. Synthesis of 24 by reaction of $10[Ga_2Cl_7]$ wit 2 equiv. of NHC L 19

2.13. Synthesis of $LP_3Pd(PPh_3)_2[GaCl_4]$ ($25[GaCl_4]$) 23

2.14. Variable temperature NMR studies 25

2.15. Compounds possessing a P_3H_3 structural unit and their P NMR chemical shifts 26

3. X-ray Diffraction Refinements 27

3.1. General remarks 27

3.2. Crystallographic data 29

4. Computational details 35

5. References 49

1. General remarks, materials and methods

All reported relevant compounds are fully characterized by multinuclear NMR spectroscopy, IR- and Raman spectroscopy, elemental analysis and X-ray crystallography. Manipulations were performed in a Glovebox MB Unilab or using Schlenk techniques under an atmosphere of purified nitrogen or argon, respectively. Dry, oxygen-free solvents (CH_2Cl_2, C_6H_5F, *o*-$C_6H_4F_2$ (distilled from CaH_2), *n*-hexane, *n*-pentane (distilled from potassium)) were employed. Deuterated benzene (C_6D_6) was purchased from Sigma-Aldrich and distilled from potassium. and dichloromethane (CD_2Cl_2) were purchased from Sigma-Aldrich. All distilled and deuterated solvents were stored over molecular sieves (4 Å). All glassware was oven-dried at 160 °C prior to use. Compound **6**[$GaCl_4$][1], NHC **L**[2], HDMAP[Cl][3] were prepared according to literature procedures. Et_3NH[Cl] and Et_4N[Cl] were purchased from Sigma Aldrich and were dried according to a previously published procedure.[4] Pd(PPh_3)$_4$, was purchased from Sigma Aldrich and was used as received. Ga[Ga_2Cl_7] was purchased from Shanghai Richem International and was used as received. NMR spectra were measured on a Bruker AVANCE III HD Nanobay (^{1}H (400.13 MHz), ^{13}C (100.61 MHz), ^{31}P (161.98 MHz) ^{195}Pt (86.01 MHz)) 400 MHz UltraSield or on a Bruker AVANCE III HDX, 500 MHz Ascend (^{1}H (500.13 MHz), ^{13}C (125.75 MHz), ^{31}P (202.45 MHz)). All ^{13}C NMR spectra were exclusively recorded with composite pulse decoupling. Reported numbers assigning atoms in the ^{13}C spectra were indirectly deduced from the cross-peaks in 2D correlation experiments (HMBC, HSQC). Chemical shifts were referenced to $\delta_{TMS} = 0.00$ ppm (^{1}H, ^{13}C), $\delta_{H3PO4(85\%)} = 0.00$ ppm (^{31}P) and $\delta_{K2PtCl6} = 0.00$ ppm (^{195}Pt). Chemical shifts (δ) are reported in ppm. Coupling constants (J) are reported in Hz. The designation of the spin system was performed by convention. The furthest down field resonance is denoted by the latest letter in the alphabet, and the furthest upfield by the earliest letter. Melting points were recorded on an electrothermal melting point apparatus (Büchi Switzerland, Melting point M-560) in sealed capillaries under Argon atmosphere and are uncorrected. Infrared (IR) and Raman spectra were recorded at ambient temperature using a Bruker Vertex 70 instrument equipped with a RAM II module (Nd-YAG laser, 1064 nm). The Raman intensities are reported in percent relative to the most intense peak and are given in parenthesis. An ATR unit (diamond) was used for recording IR spectra. The intensities are reported relative to the most intense peak and are given in parenthesis using the following abbreviations: vw = very weak, w = weak, m = medium, s = strong, vs = very strong. Elemental analyses were performed on a Vario MICRO cube Elemental Analyzer by Elementar Analysatorsysteme GmbH in CHNS mode.

2. Synthetic details and characterization data

2.1. Reaction of $L_2P_3[GaCl_4]$ (6[Ga_2Cl_7]) with HOTf

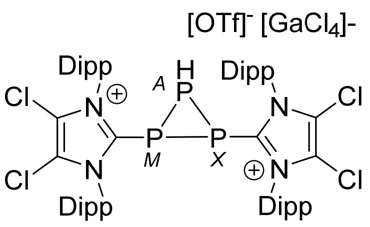

A solution of **6**[$GaCl_4$] (62 mg, 0.05 mmol, 1.0 eq.) in C_6H_5F / benzene (1 : 1 mixture, 5 mL) was stirred at ambient temperature. A solution of HOTf (30 mg, 0.2 mmol, 4.0 eq.) in C_6H_5F / benzene (1 : 1 mixture, 2 mL) was added dropwise within 1 min giving rapidly an orange oil and a yellowish supernatant solution. The supernatant was removed and the oil was washed with *n*-hexane (3 x 3 mL) yielding an orange solid. The material was dissolved in CD_2Cl_2 and investigated by means of $^{31}P\{^1H\}$ NMR spectroscopy. CD_2Cl_2 was removed *in vacuo* and the residue was dissolved in 1,2-$C_6H_4F_2$. Single crystals of **8**[OTf*$GaCl_3$]$_2$, suitable for X-ray single crystal structure determination but covered with an orange oil, were obtained by slow diffusion of *n*-hexane into the 1,2-$C_6H_4F_2$ solution of the respective compounds at –35 °C.

^{31}P{^{1}H} NMR (CD_2Cl_2, 300 K, in ppm): AMX spin system of **8**$^{2+}$: δ (P_A) = –202.3, δ(P_M) = –182.6, δ(P_X) = –156.8, $^1J(P_AP_M)$ = –158.8 Hz, $^1J(P_AP_X)$ = –130.4 Hz, $^1J(P_MP_X)$ = –203.0 Hz; **^{31}P NMR** (CD_2Cl_2, 300 K, in ppm): AMXZ spin system of **8**$^{2+}$: $^1J(P_AH_Z)$ = 155.7 Hz, $^2J(P_MH_Z)$ = 17.8 Hz, $^2J(P_XH_Z)$ = 34.2 Hz.

The addition of one equivalent of **NHC** (**L**) to a colourless solution of **8**$^{2+}$in C_6H_5F gives a deep green solution of **6**$^+$. Subsequent addition of one equivalent of HOTf gives a colourless solution of **8**$^+$ again without significant decomposition as monitored by $^{31}P\{^1H\}$ NMR spectroscopy.

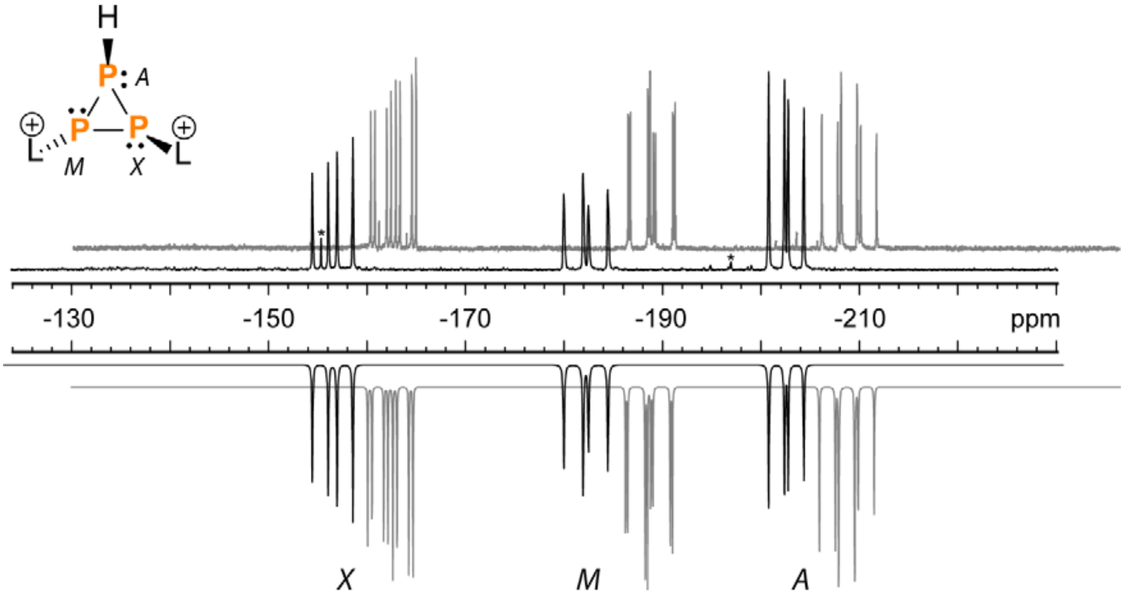

Fig. S1. Experimental $^{31}P\{^1H\}$ (upwards, black) and ^{31}P NMR spectrum (upwards, grey) and simulated $^{31}P\{^1H\}$ NMR spectrum (downwards, black) and ^{31}P NMR spectrum (downwards, grey) of **8**$^{2+}$ (CD_2Cl_2, 300 K); small amounts of unidentified side products are marked with asterisks.

2.2. Reaction of 6[$GaCl_4$] with [PCl_4][$GaCl_4$] and $GaCl_3$ (1:1:2)

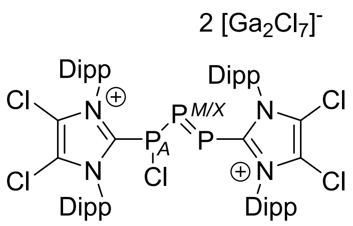

To the green solution of **6**[$GaCl_4$] (34.0 mg, 0.0279 mmol) and $GaCl_3$ (9.2 mg, 0.052 mmol, 2 eq.) in 2 mL of dichloromethane solid [PCl_4][$GaCl_4$] (10.7 mg, 0.0278 mmol, 1 eq.) was added resulting in the immediate colour change to dark red. After NMR analysis of the solution all volatiles were removed *in vacuo*. The red residue was dissolved in *o*-difluorobenzene and cooled to −30 °C for crystallization of **9Cl**[$GaCl_4$][Ga_2Cl_7] · 2.681 *o*-$C_6H_4F_2$ · 0.319 C_5H_{12} by vapour diffusion with *n*-pentane.

^{31}P{^{1}H} (202 MHz, C_6D_6-cap., 300 K, in ppm): δ = 56.0 (1P, dd, $J(P_AP_X)$ = 202 Hz, $J(P_AP_M)$ = 715 Hz, P_A), 454.8 (1P, dd, $J(P_MP_X)$ = 560 Hz, $J(P_AP_M)$ = 715 Hz, P_M), 533.8 (1P, dd, $J(P_AP_X)$ = 202 Hz, $J(P_MP_X)$ = 560 Hz, P_X).

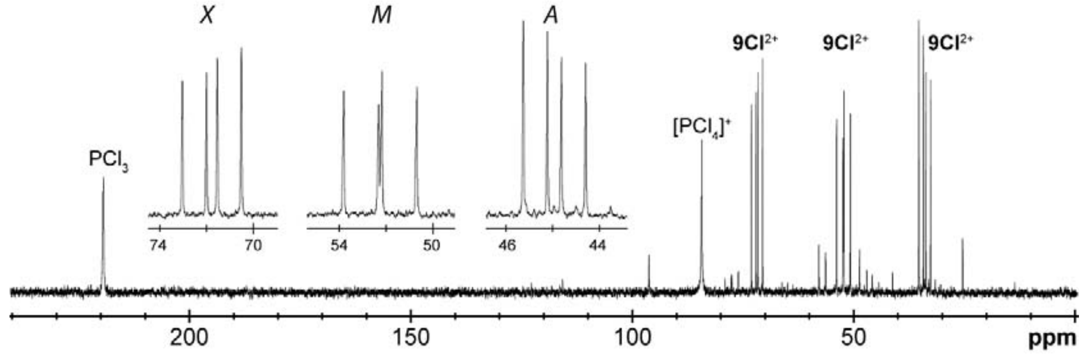

Fig. S2. ^{31}P{^{1}H} NMR spectrum of the reaction mixture **6**[$GaCl_4$] with [PCl_4][Cl] and 2 $GaCl_3$ in CH_2Cl_2 (C_6D_6-capillary, 300 K).

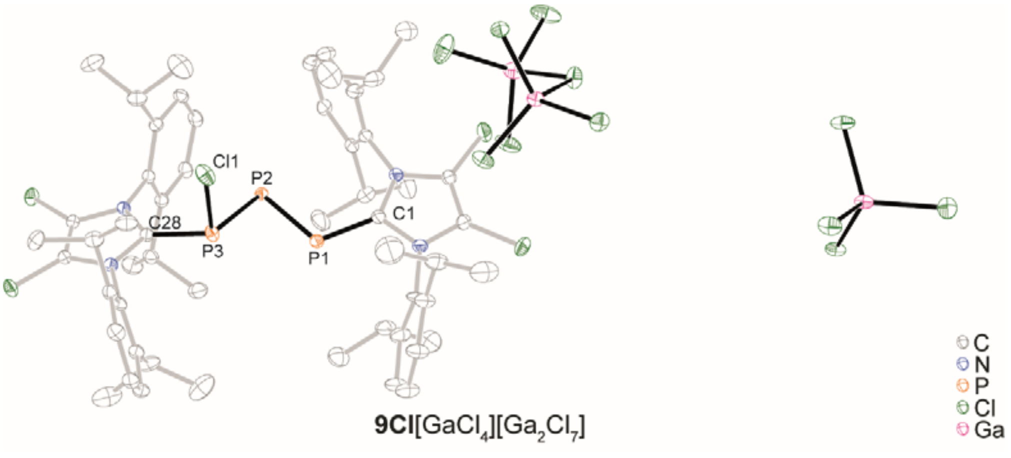

Fig. S3. Molecular structure of **9Cl**[$GaCl_4$][Ga_2Cl_7]·3 $C_6H_4F_2$; hydrogen atoms, solvent molecules and disorder are omitted for clarity; ellipsoids are drawn at 50 % probability level.

2.3. Synthesis of $L_2P_3(Ga_2Cl_4)[Ga_2Cl_7]$ (10[Ga_2Cl_7])

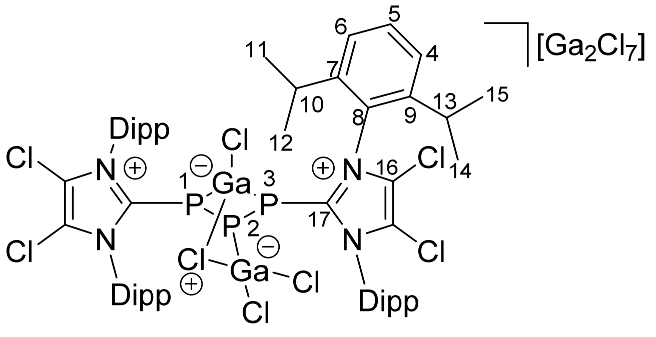

2.82 g of **6**[$GaCl_4$] (1 eq., 2.3 mmol) and 1.06 g Ga[Ga_2Cl_7] (1 eq., 2.3 mmol) were suspended in 55 ml fluorobenzene at -40 °C. The green suspension was allowed to warm to room temperature while stirring overnight to give a brown solution. The solvent was removed *in vacuo* and the residue recrystallized multiple times from a CH_2Cl_2 / *n*-hexane mixture (60 ml : 40 ml) at -30 °C. After filtration and drying *in vacuo*, **10**[Ga_2Cl_7] (2.16 g, 55%) was obtained as air sensitive yellow crystalline solid.

^{1}H NMR (CD_2Cl_2, 300 K, in ppm): 1.08 (12H, d, $^3J_{HH}$ = 6.7 Hz, H15), 1.10 (12H, d, $^3J_{HH}$ = 6.7 Hz, H11), 1.16 (12H, d, $^3J_{HH}$ = 6.8 Hz, H12), 1.26 (12H, d, $^3J_{HH}$ = 6.8 Hz, H14), 2.28 (4H, b, H10), 2.36 (4H, b, H13), 7.34 (4H, d, $^3J_{HH}$ = 7.7 Hz, H4), 7.39 (4H, d, $^3J_{HH}$ = 7.7 Hz, H6), 7.69 (4H, t, $^3J_{HH}$ = 7.7 Hz, H5); **^{13}C{^{1}H} NMR** (CD_2Cl_2, 300 K, in ppm): 24.1 (4C, C14), 24.8 (12C, C11,12,15), 29.9 (4C, C13), 30.1 (4C, C10), 124.8 (4C, C16), 126.9 (4C, C6), 127.1 (4C, C4), 128.7 (4C, C8), 134.1 (4C, C5), 146.0 (4C, C9), 146.5 (4C, C7), 154.0 (2C, C17, $^1J_{PC}$ = 133 Hz); **^{31}P{} NMR** (CD_2Cl_2, 300 K, in ppm): -119.7 (1P, t, -277 Hz, P2); 5.0 (2P, b, P1,3); **Raman** (100 mW, 1000 scans, 298 K, [cm^{-1}]): 3072 (13), 2970 (21), 2939 (34), 2914 (28), 2871 (16), 1584 (25), 1467 (12), 1443 (13), 1393 (16), 1359 (79), 1330 (93), 1318 (100), 1294 (40), 1267 (16), 1239 (10), 1186 (5), 1167 (6), 1104 (8), 1049 (12), 987 (9), 888 (14); **IR** (ATR, 298 K, [cm^{-1}]): 2967 (w), 2930 (w), 2872 (w), 1573 (m), 1460 (w), 1441 (w), 1387 (m), 1366 (m), 1326 (w), 1301 (w), 1273 (w), 1182 (w), 1168 (w), 1109 (w), 1060 (w), 1047 (w), 935 (w), 802 (m), 760 (w), 732 (w), 692 (w), 668 (w), 543 (w), 416 (vs), 400 (vs), 390 (vs), 375 (vs), 358 (s); **Elemental analysis**: $C_{54}H_{68}Cl_{15}Ga_4N_4P_3$: calcd.: N: 3.3, C: 38.7, H: 4.1; found: N: 3.3, C: 38.5; H: 4.2; **m.p.:** 153 °C (decomposition).

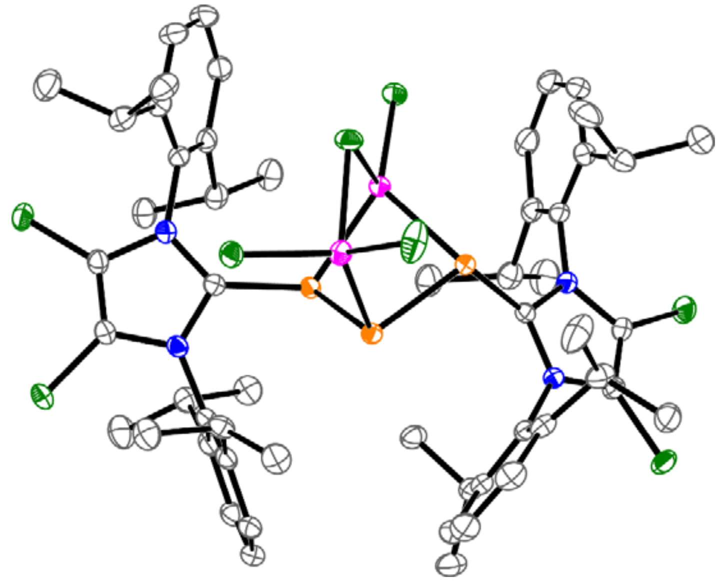

Fig. S4. Molecular structure of 10^{+} in **10**[Ga_2Cl_7]·CH_2Cl_2; thermal ellipsoids are displayed at 50 % probability level; hydrogen atoms, solvent molecules and the anion are omitted for clarity.

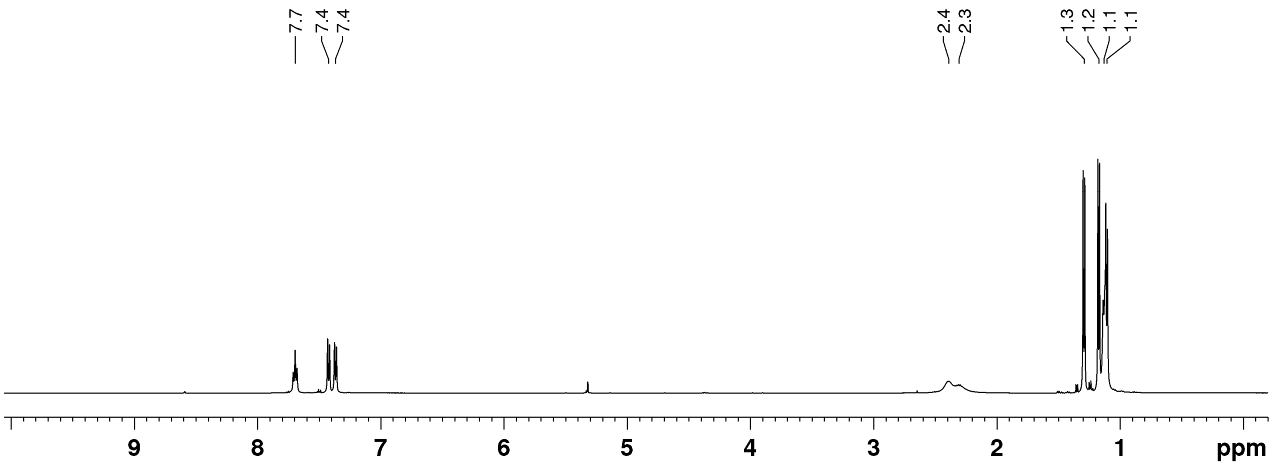

Fig. S5. ^{1}H NMR spectrum (CD_2Cl_2, 300 K) of **10**[Ga_2Cl_7].

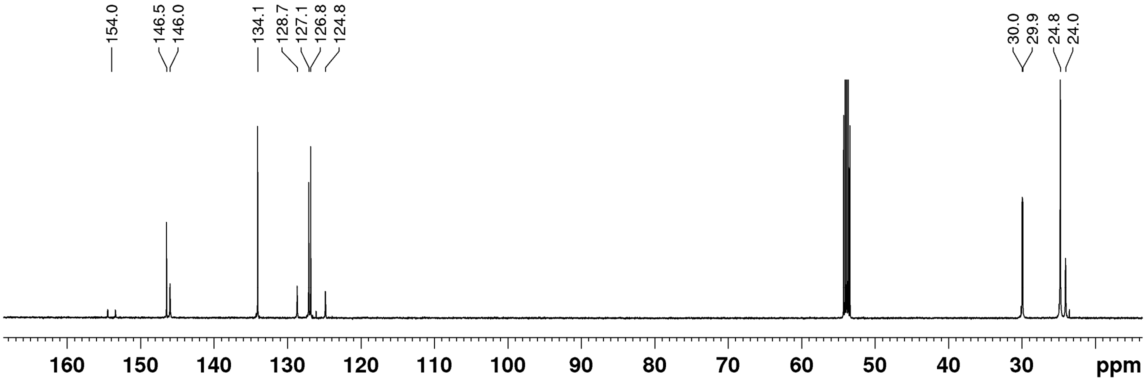

Fig. S6. $^{13}C\{^{1}H\}$ NMR spectrum (CD_2Cl_2, 300 K) of **10**[Ga_2Cl_7].

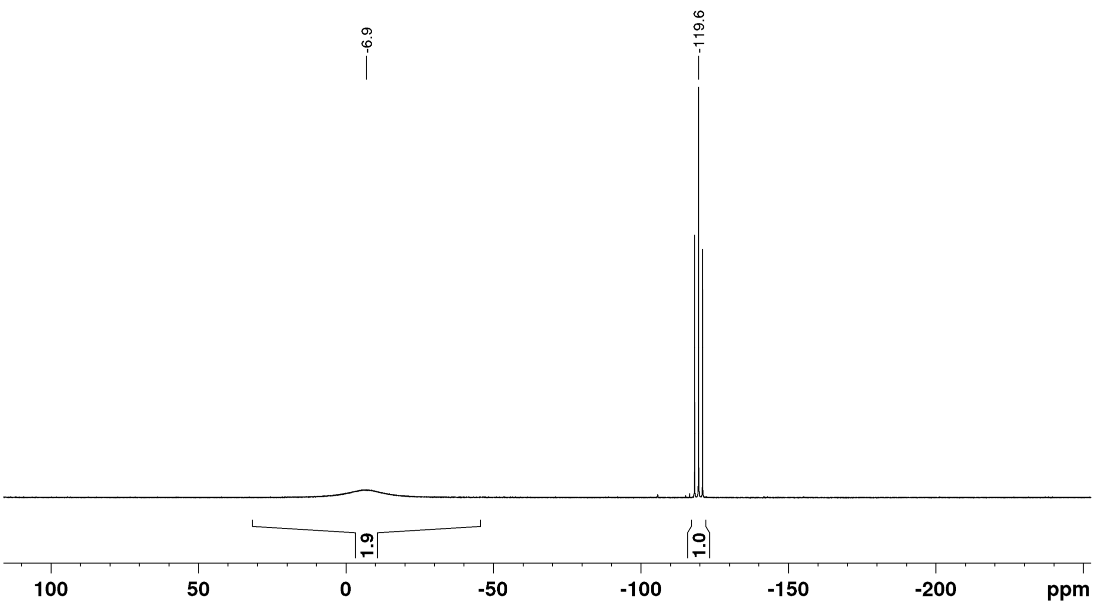

Fig. S7. ^{31}P NMR spectrum (CD_2Cl_2, 300 K) of **10**[Ga_2Cl_7].

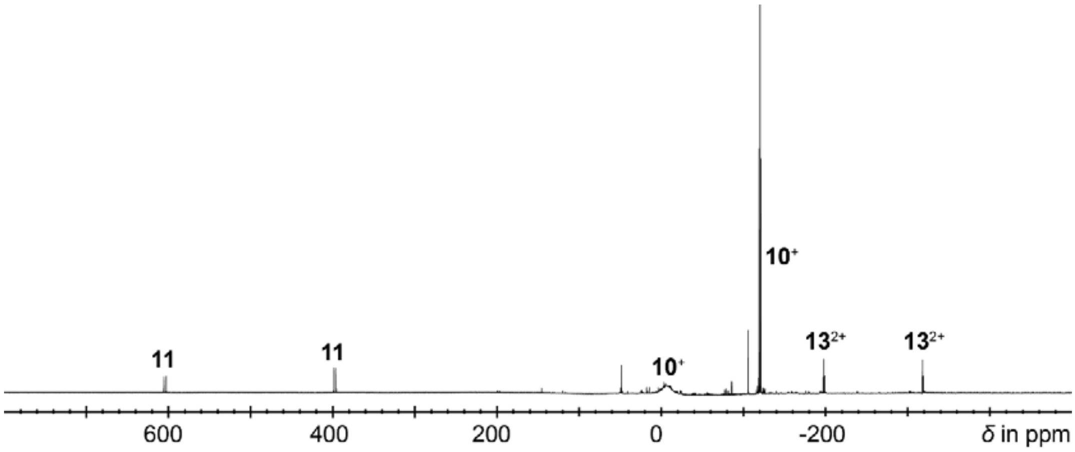

Fig. S8. ^{31}P NMR spectrum (C_6H_5F, 300 K) of a reaction mixture of **6**[$GaCl_4$] and Ga[Ga_2Cl_7].

2.4. Hydrolysis of $L_2P_3(Ga_2Cl_4)[Ga_2Cl_7]$ (**10**[Ga_2Cl_7]) with water

To an orange solution of **10**[Ga_2Cl_7] (50.0 mg, 0.0298 mmol) in 1 mL of dichloromethane 1.6 mL of a stock solution of water in dichloromethane (0.0184 M, 0.294 mmol) were added. After 3 h and NMR measurement on this solution a second equivalent of water (1.6 mL, 0.294 mmol) was added yielding a pale yellow solution. NMR analysis revealed full conversion of the starting material.

R,s,S-$\mathbf{14}^{2+}$: ^{31}**P NMR** (C_6D_6-cap., 300 K, in ppm): δ = -140.5 (1P, dtt, $^1J_{P(A)H}$ = 191 Hz, $^2J_{P(A)H}$ = 15 Hz, $^1J_{P(A)P(X)}$ = -117 Hz, P_A), -90.3 (2P, dddd, $^1J_{P(X)H}$ = 181 Hz, $J_{P(X)H}$ = 58 Hz, $J_{P(X)H}$ = 13 Hz, $^1J_{P(A)P(X)}$ = -117 Hz, P_X).

rac-$\mathbf{14}^{2+}$: ^{31}**P NMR** (C_6D_6-cap., 300 K, in ppm): δ = -131.6 (1P, ddd, $^1J_{P(A)H}$ = 189 Hz, $^2J_{P(A)H}$ = 25 Hz, $^2J_{P(A)H}$ = 5 Hz, $^1J_{P(A)P(X)}$ = -146 Hz, $^1J_{P(A)P(M)}$ = -132 Hz, P_A), -109.7 (1P, ddd, $^1J_{P(M)H}$ = 229 Hz, $J_{P(M)H}$ = 31 Hz, $J_{P(M)H}$ = 6 Hz, $^2J_{P(M)P(X)}$ = 282 Hz, $^1J_{P(A)P(M)}$ = -132 Hz, P_M), -105.5 (1P, ddd, $^1J_{P(X)H}$ = 229 Hz, $J_{P(X)H}$ = 24 Hz, $J_{P(X)H}$ = 4 Hz, $^1J_{P(A)P(X)}$ = -146 Hz, $^2J_{P(M)P(X)}$ = 282 Hz, P_X).

rac-$\mathbf{14}^{+}$: ^{31}**P NMR** (C_6D_6-cap., 300 K, in ppm): δ = -144.2 (1P, tt, $^2J_{P(A)H}$ = 9 Hz, $^1J_{P(A)P(X)}$ = -223 Hz, P_A), -90.3 (2P, td, $^1J_{P(X)H}$ = 170 Hz, $^3J_{P(X)H}$ = 95 Hz, $^1J_{P(A)P(X)}$ = -223 Hz, P_X).

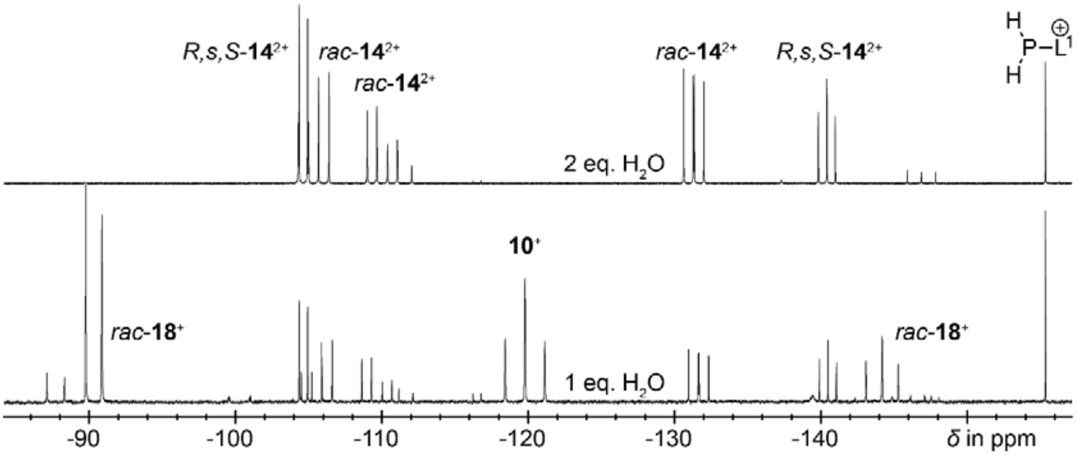

Fig. S9. $^{31}P\{^1H\}$ NMR spectra of the reaction mixture of **10**[Ga_2Cl_7] after addition of 1 and 2 eq. of water (C_6D_6-cap., CH_2Cl_2, 300K).

In the reaction mixture of a 1:1 mixture of **10**[Ga_2Cl_7] and water in CH_2Cl_2 an intermediate with A_2X spin system bearing only two hydrogen atoms was identified by $^{31}P\{^1H\}$ NMR spectroscopy. The species is assumed to be mono-cation **18**$^+$ (**Fig. S**, top right) However, it cannot be completely excluded that the central P atom still bears a gallium based moiety.

2.5. Hydrolysis of $L_2P_3(Ga_2Cl_4)[Ga_2Cl_7]$ (10[Ga_2Cl_7]) with HCl

To a solution of **10**[Ga_2Cl_7] (50.0 mg, 0.0298 mmol) in 2 mL of CH_2Cl_2 45 µL of HCl in diethyl ether (2 M, 0.90 mmol) were added. The $^{31}P\{^1H\}$ NMR spectrum of the reaction solution is mostly identical to that of the addition of 2 eq. of water (see **Fig. S**). After NMR analysis the solution was set for crystallization by vapour diffusion with n-pentane at -30 °C. X ray diffraction analysis proved the formation of **14**[$(Cl_3Ga)OH]_2$.

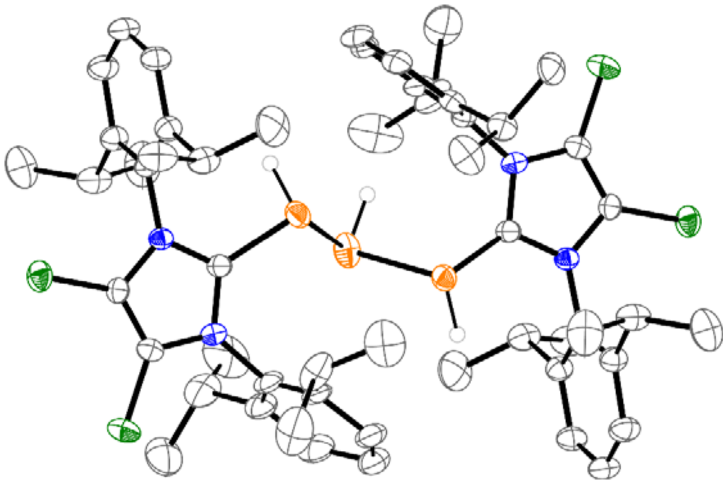

Fig. S10. Molecular structure of **14**$^{2+}$ in **14**[$(Cl_3Ga)_2OH]_2$; thermal ellipsoids are displayed at 50 % probability level; hydrogen atoms, solvent molecules and the anion are omitted for clarity.

2.6. Reaction of 10[Ga_2Cl_7] with HDMAP[Cl]

HDMAP[Cl] (7.5 mg, 0.047 mmol) in 3 ml CH_2Cl_2 was added to a solution of **10**[Ga_2Cl_7] (80 mg, 0.047 mmol) in 3 ml CH_2Cl_2. After stirring for 4 h at room temperature the solvent was removed *in vacuo*. The residue was washed with 1 ml benzene und 1 ml C_6H_5F to remove the byproduct HDMAP[$GaCl_4$]. The resulting orange solid contains **22** and **L**H[$GaCl_4$].

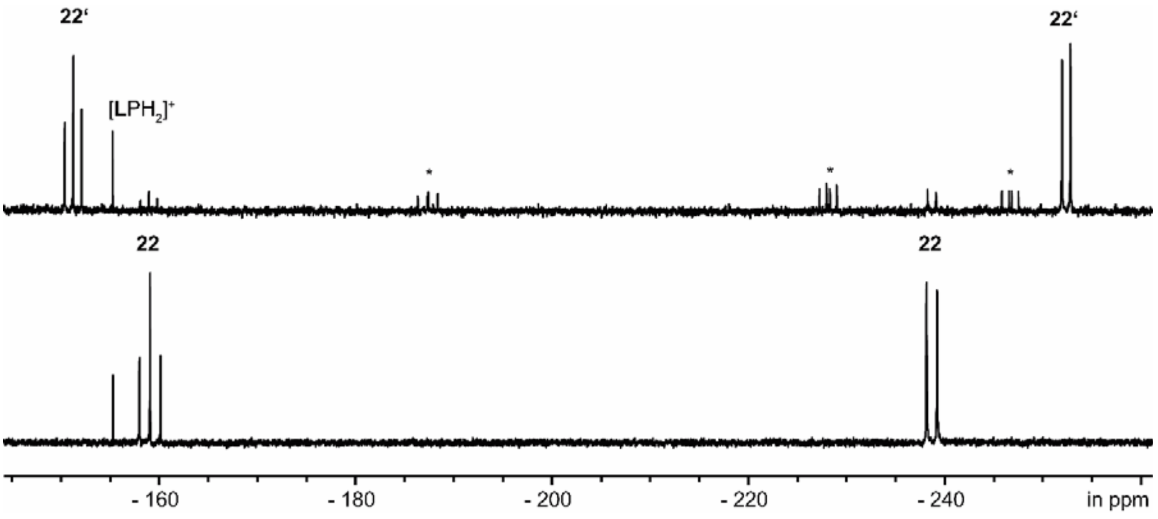

Fig. S11. $^{31}P\{^1H\}$ NMR (CD_2Cl_2, 300 K) of **22** (bottom) and decomposition product of **22** in the presence of HDMAP[Cl] (top); the resonances can be assigned as follows: $\delta(P_A) = -238.6$ ppm, $\delta(P_X) = -158.8$ ppm (**22**), $\delta(P_A) = -252.4$ ppm, $\delta(P_X) = -151.3$ ppm (**22’**), $\delta(P) = -155.4$ ppm ([LPH_2]$^+$); asterisk assigned to unidentified compound.

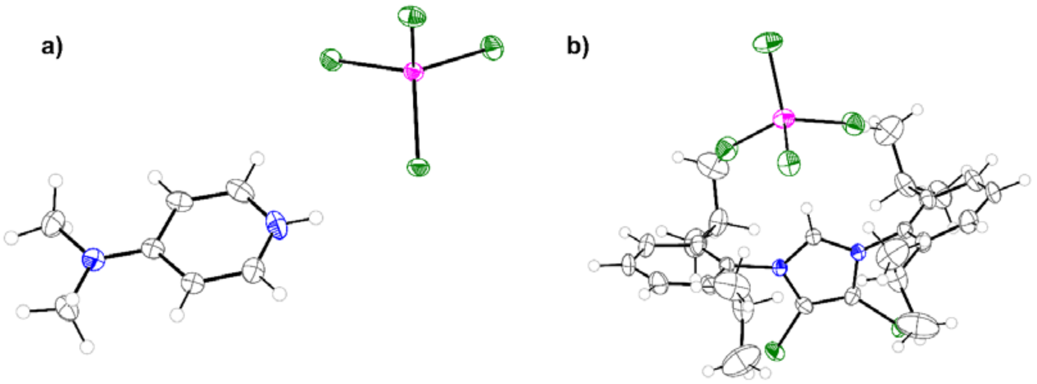

Fig. S12. Molecular structures of a) HDMAP[$GaCl_4$] and b) **L**H[$GaCl_4$]; thermal ellipsoids are displayed at 50 % probability level.

2.7. Synthesis of 22 by reaction of 10[Ga_2Cl_7] with Et_4N[Cl]

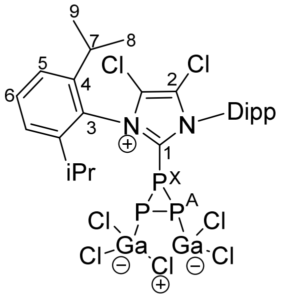

A solution of [Et_4N[Cl]] (6 mg, 0.05 mmol) in 0.5 ml CH_2Cl_2 was added dropwise to a solution of **10**[Ga_2Cl_7] (60 mg, 0.05 mmol) in 1 ml CH_2Cl_2. After 3 h the resulting solution was concentrated *in vacuo* and 4 ml *n*-pentane were added. The suspension was filtered and the resulting solid was dried *in vacuo* and dissolved in CH_2Cl_2. By slow diffusion of *n*-hexane into this saturated solution, **22** was isolated by fractional crystallization. The first crystal fraction was identified mostly as Et_4N[$GaCl_4$] and **LH**[$GaCl_4$]. The residual pale green solution was further used for crystallization and the second fraction contained **22** (5 mg, 16 %). **22** turned out to be extremely sensitive to hydrolysis even under strict inert atmosphere.

^{1}H NMR (CD_2Cl_2, 300 K, in ppm): δ = 1.24 (12H, d, $^3J_{HH}$ = 6.8 Hz, H8), 1.48 (12H, d, $^3J_{HH}$ = 6.8 Hz, H9), 2.23 (4H, sept, $^3J_{HH}$ = 6.9 Hz, H7), 7.49 (4H, d, $^3J_{HH}$ = 7.7 Hz, H5), 7.76 (2H, 7, $^3J_{HH}$ = 7.7 Hz, H6). **^{13}C{^{1}H} NMR** (CD_2Cl_2, 300 K, in ppm): δ = 24.0 (4C, s, C9), 25.0 (4C, s, C8), 30.2 (4C, s, C7), 124.1 (4C, d, ^3J(PC) = 4 Hz, C2), 126.4 (4C, s, C5), 127.6 (2C, s, C3), 134.1 (2C, s, C6), 146.2 (4C, s, C4), C1 was not observed due to signal broadening. **^{31}P{} NMR** (CD_2Cl_2, 300 K, in ppm): δ = -238.6 (2P, d, $^1J_{P(A)P(X)}$ = -175 Hz, P_A), -158.8 (1P, t, $^1J_{P(A)P(X)}$ = -175 Hz, P_X). **IR** (ATR, 298 K, in cm^{-1}): ν = 2965 (s), 2927 (m), 2870 (m), 1561 (vs), 1462 (s), 1442 (s), 1389 (s), 1366 (s), 1326 (m), 1275 (m), 1181 (s), 1110 (m), 1059 (m), 936 (m), 804 (vs), 761 (s), 543 (m), 531 (m), 436 (m), 420 (s). **Raman**: Due to fluorescence, the Raman spectrum could not be recorded. **Elemental analysis**: $C_{27}H_{34}Cl_7Ga_2N_2P_3 \cdot 0.5\ C_6H_{14}$: calcd.: N: 3.1, C: 39.6, H: 4.5; found: N: 3.4, C: 39.4; H: 4.2; **Melting point:** 220-222 °C (decomposition).

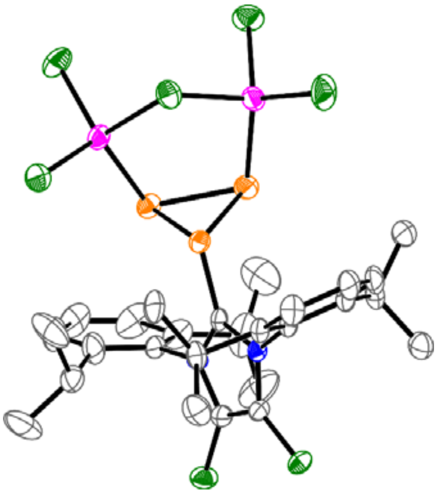

Fig. S13. Molecular structure of **22**; thermal ellipsoids are displayed at 50 % probability level; hydrogen atoms are omitted for clarity.

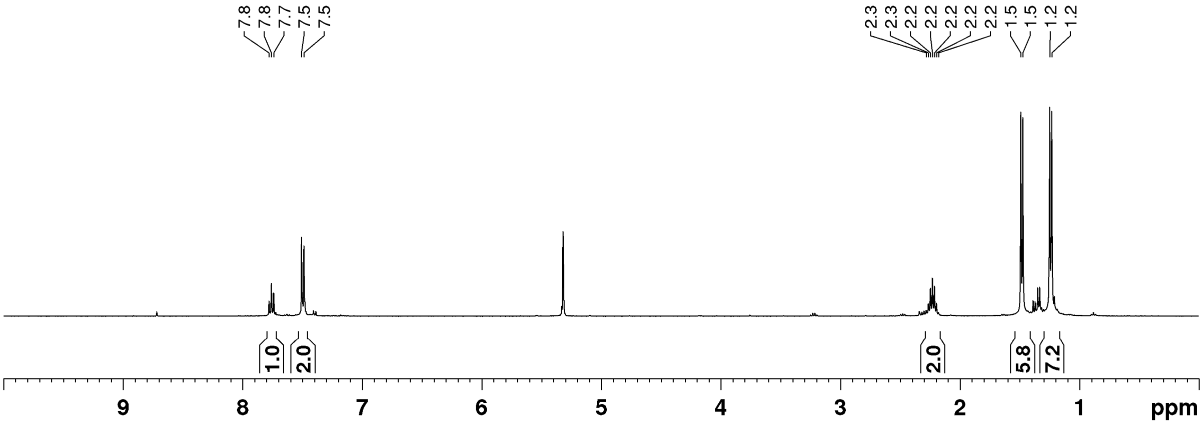

Fig. S14. ^{1}H NMR spectrum (CD_2Cl_2, 300 K) of **22**.

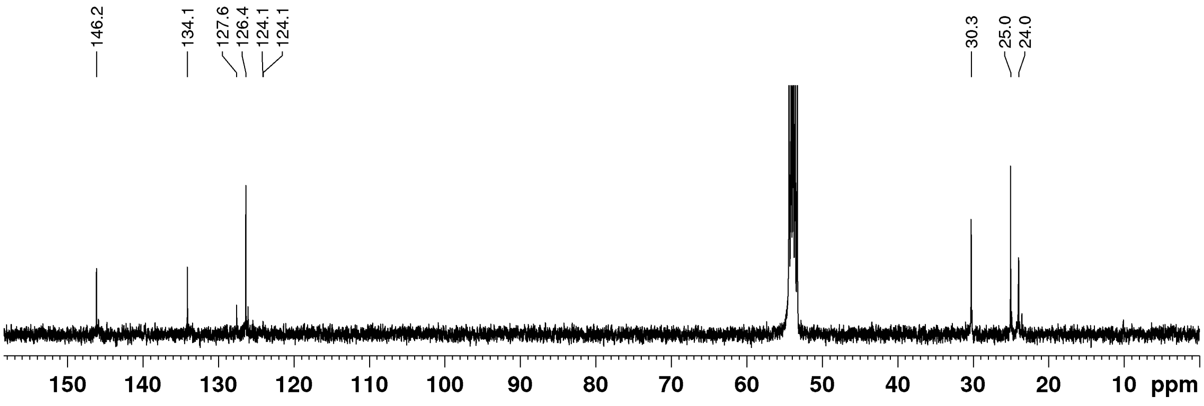

Fig. S15. $^{13}C\{^{1}H\}$ NMR spectrum (CD_2Cl_2, 300 K) of **22**.

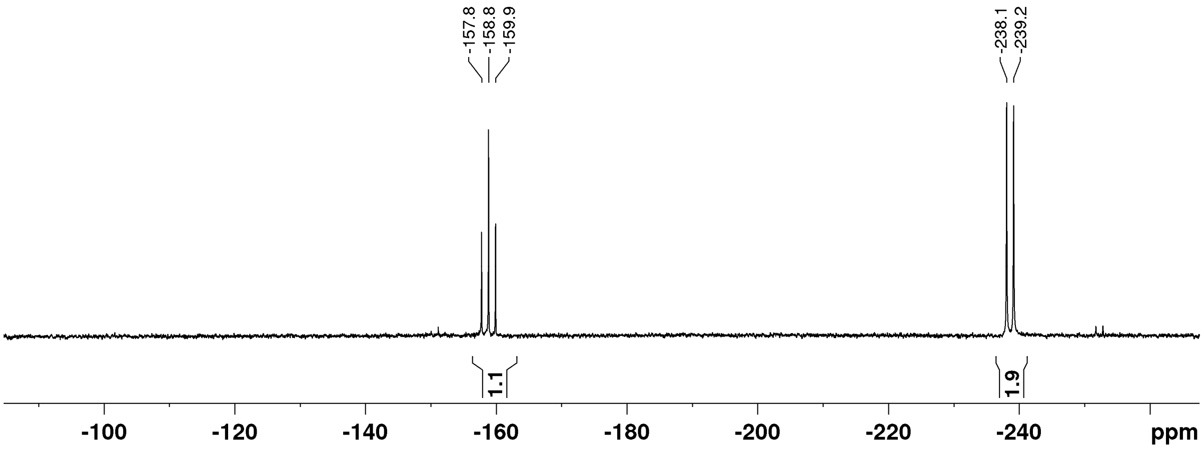

Fig. S16. ^{31}P NMR spectrum (CD_2Cl_2, 300 K) of **22**

From the solution that was used for NMR measurements small crystals formed which were identified by X-Ray diffraction as **22'**, the hydrolysis product of **22**.

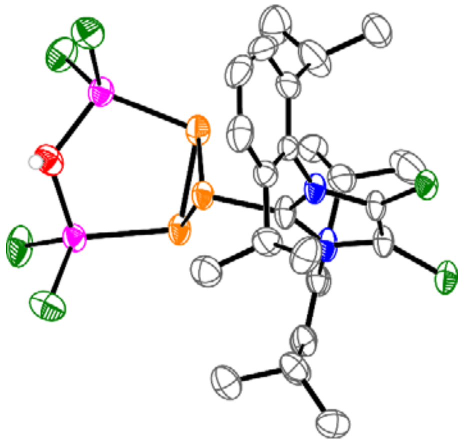

Fig. S17. Molecular structure of **22'** in **22'**·CH_2Cl_2·0.5 *n*-Hexane; thermal ellipsoids are displayed at 50 % probability level; hydrogen atoms are omitted for clarity.

2.8. Synthesis of $Et_3NH[LP_3(GaCl_3)_2]$ (Et_3NH[23]) by reaction of 10[Ga_2Cl_7] with Et_3NH[Cl]

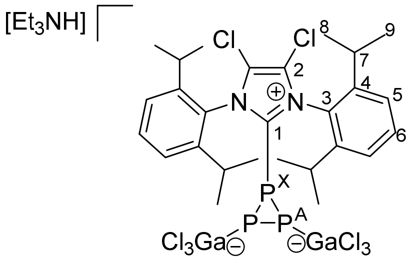

A solution of Et_3NH[Cl] (24 mg, 0.06 mmol) in 3 ml CH_2Cl_2 was added to a solution of **10**[Ga_2Cl_7] (100 mg, 0.02 mmol) in 5 ml CH_2Cl_2. The solvent was removed *in vacuo* after stirring for 4 h at room temperature. The residue was washed with *o*-$C_6H_4F_2$. Et_3NH[**23**] with by-product **L**H[$GaCl_4$] was precipitated from the saturated *o*-$C_6H_4F_2$ solution with *n*-pentane. Et_3NH[**23**] (12 mg, 20 %) was isolated by fractional crystallization by diffusion of *n*-pentane into a saturated C_6H_5F solution at –30 °C as red crystals. The purity of the resulting crystals was determined by elemental analysis. Et_3NH[**23**] is unstable in solution, resulting in fast decomposition within minutes. We therefore abstain of any interpretation of the shown NMR data (see Figures **SI 14-15**).

^{1}H NMR (CD_2Cl_2, 300 K, in ppm): δ = 1.22 (12H, d, ^3J(HH) = 6.8 Hz, H8), 1.35 (9H, t, ^3J(HH) = 7.4 Hz, CH_3 (Et_3NH)), 1.50 (12H, d, ^3J(HH) = 6.8 Hz, H9), 2.27 (4H, sept, ^3J(HH) = 6.8 Hz, H7), 3.22 (6H, q, ^3J(HH) = 7.4 Hz, CH_2 (Et_3NH)), 7.03 (1H, s, NH) 7.46 (4H, d, ^3J(HH) = 7.7 Hz, H5), 7.70 (2H, t, ^3J(HH) = 7.7 Hz, H6). **^{13}C{^{1}H} NMR** (CD_2Cl_2, 300 K, in ppm): δ = 9.3 (3C, s, CH_3(Et_3NH)), 23.9 (4C, s, C8), 25.1 (4C, s, C9), 30.2 (4C, s, C7), 48.0 (3C, CH_2(Et_3NH)), 123.1 (4C, m, C2), 126.2 (4C, s, C5), 128.0 (2C, s, C3), 133.5 (2C, s, C6), 146.0 (4C, s, C4), C1 was not observed due to signal broadening. **^{31}P NMR** (CD_2Cl_2, 300 K, in ppm): δ = -216.6 (2P, d, $^1J_{P(A)P(X)}$ = -185 Hz, P_A), -142.4 (1P, t, $^1J_{P(A)P(X)}$ = -185 Hz, P_X). **IR** (ATR, 298 K, in cm^{-1}): ν = 3096 (m), 2967 (s), 2930 (m), 2872 (m), 2722 (w), 2690 (w), 1567 (m), 1520 (m), 1461 (s), 1390 (s), 1368 (m), 1355 (w), 1330 (m), 1301 (w), 1278 (w), 1257 (w), 1243 (w), 1198 (m), 1182 (m), 1156 (w), 1110 (w), 1060 (m), 1047 (w), 1030 (m), 1012 (w), 937 (w), 807 (vs), 760 (s), 669 (w), 593 (w), 544 (m), 431 (w). **Raman:** Due to fluorescence, the Raman spectrum could not be

recorded. **Elemental analysis**: $C_{33}H_{50}Cl_8Ga_2N_3P_3$ calc.: N: 4.2, C: 39.5, H: 4.8; found: N: 4.2, C: 39.9; H: 5.0. **Melting point**: 171-173 °C (decomposition).

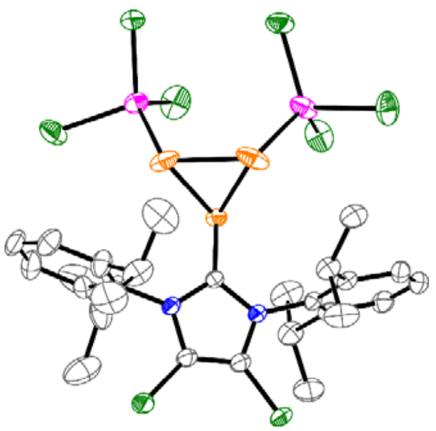

Fig. S18. Molecular structure of **23**$^-$ in Et_3NH[**23**]·C_6H_5F; thermal ellipsoids are displayed at 50 % probability level; hydrogen atoms, counter ion and solvate molecules are omitted for clarity.

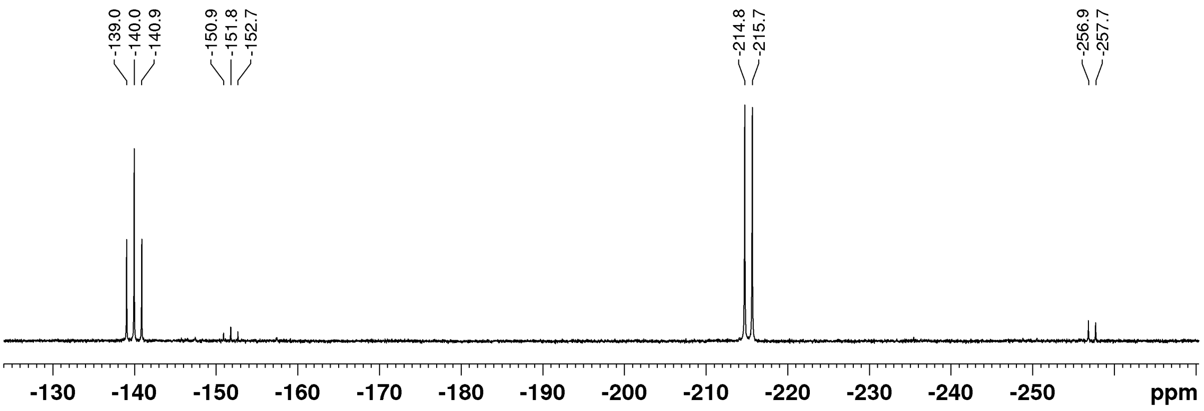

Fig. S19. ^{31}P NMR spectrum of the obtained crystals of Et_3NH[**23**]. (CD_2Cl_2, 300 K)

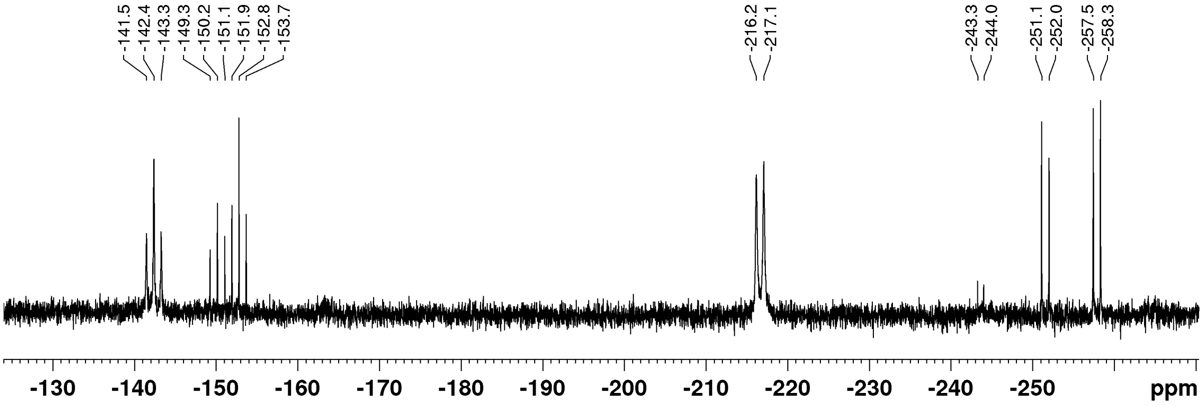

Fig. S20. ^{31}P NMR spectrum of the obtained crystals of Et_3NH[**23**] showing clear signs of decomposition after 1h in solution. (CD_2Cl_2, 300 K)

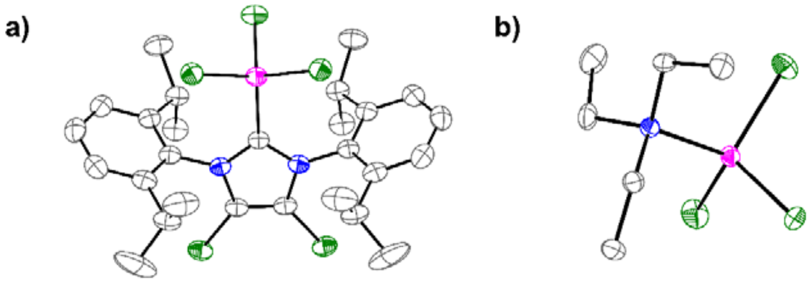

Fig. S21. Molecular structure of the side products a) **L**$GaCl_3$ in **L**$GaCl_3 \cdot CH_2Cl_2$ and b) Et_3NGaCl_3; thermal ellipsoids are displayed at 50 % probability level; hydrogen atoms, counter ion and solvate molecules are omitted for clarity.

2.9. Reaction of 10[Ga_2Cl_7] with one equiv. of L

A solution of **L** (5.5 mg, 0.012 mmol) in 1 ml CH_2Cl_2 is added dropwise to a solution of **10**[Ga_2Cl_7] (20 mg, 0.012 mmol) in 1 ml CH_2Cl_2. The reaction darkens slightly within 15 min and is subsequently submitted to NMR analysis. The ^{31}P NMR spectrum of the reaction mixture shows clean formation of **22** in solution. All efforts to isolate **22** from the mixture of **22** and L-$GaCl_3$ failed, due to the high sensitivity of **22**.

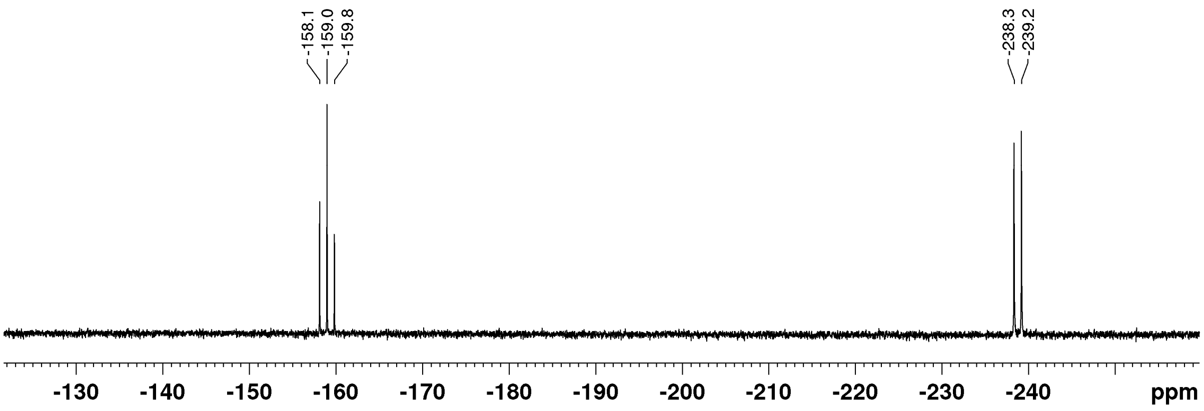

Fig. S22. ^{31}P NMR spectrum of the reaction mixture of **10**[Ga_2Cl_7]with 1 equiv. of NHC **L**. (C_6D_6 cap., CH_2Cl_2, 300 K)

2.10. Reaction of10[Ga_2Cl_7] with an excess of L

L (15 mg, 0.033 mmol) is added to a solution of **10**[Ga_2Cl_7] (20 mg, 0.012 mmol) in 0.6 ml CD_2Cl_2. The reaction darkens slightly within seconds and is subsequently submitted to NMR analysis. The ^{31}P NMR spectrum of the reaction mixture shows clean formation of **24** in solution.

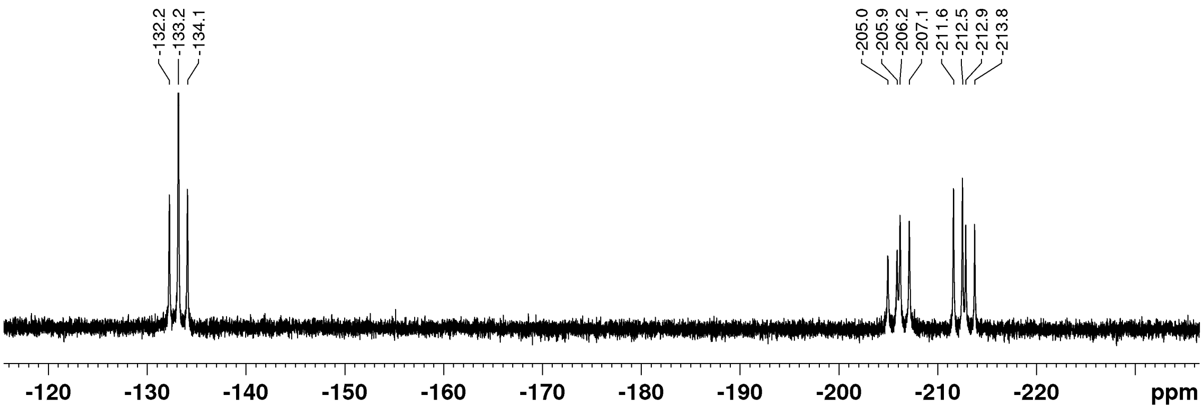

Fig. S23. ^{31}P NMR spectrum of the reaction mixture of **10**[Ga_2Cl_7] with an excess of NHC **L**. (CD_2Cl_2, 300 K).

2.11. Reaction of 10[Ga_2Cl_7] with NEt_4[Cl] and L

10[Ga_2Cl_7] (20 mg, 0.012 mmol, 1 equiv.) and NEt_4[Cl] (3.3 mg, 0.012 mmol, 1 equiv.) are stirred in CH_2Cl_2 for 2 h. Afterwards, a solution of **L** (5.5 mg, 0.012 mmol, 1 equiv.) is added. The reaction darkens slightly within seconds and is subsequently submitted to NMR analysis.

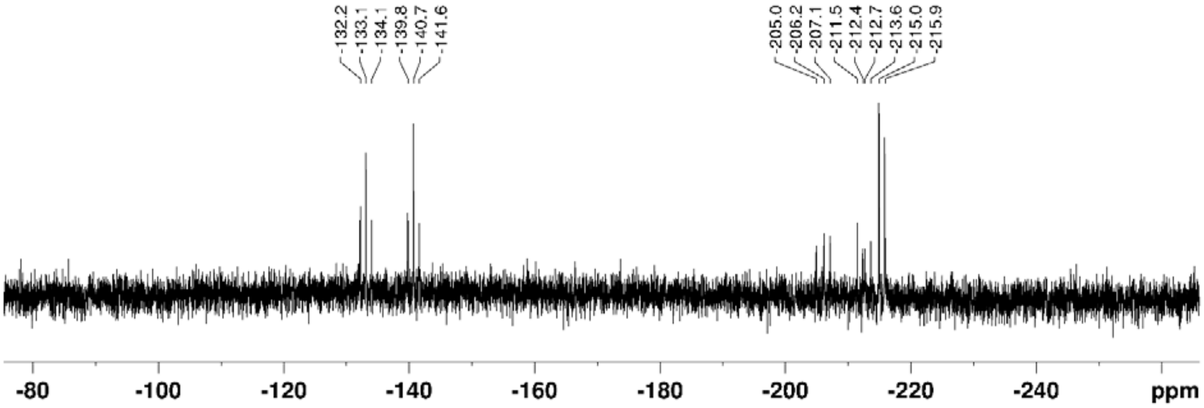

Fig. S24. ^{31}P NMR spectrum of a reaction solution of **10**[Ga_2Cl_7], NEt_4[Cl] and **L** in CH_2Cl_2 (300 K).

2.12. Synthesis of 24 by reaction of 10[Ga_2Cl_7] wit 2 equiv. of NHC L

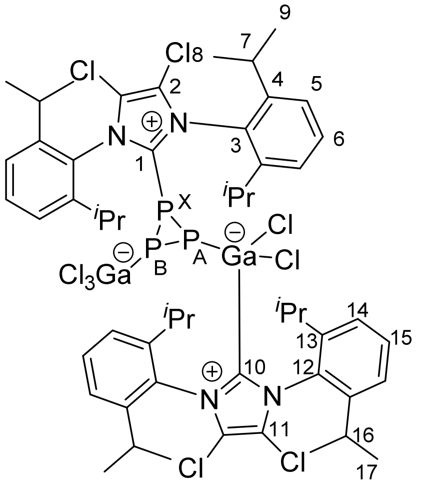

A solution of **L** (90 mg, 0.20 mmol) in 3 ml CH_2Cl_2 was added to a solution of **10**[Ga_2Cl_7] (150 mg, 0.09 mmol) in 5 ml CH_2Cl_2. The ^{31}P NMR spectrum of the reaction mixture showed full conversion of **10**[Ga_2Cl_7] to **24**. However, because of their very similar solubility **24** could not completely be separated from concurrently formed **L**-H[$GaCl_4$]. Drying the reaction mixture resulted in a dark residue which was washed with 3 ml of toluene. Small amounts of light brown powder were separated off by filtration, washed with *n*-pentane and dried *in vacuo*. The obtained powder consisted of **24** with 20% of **L**-H[$GaCl_4$] and 1 equiv. of CH_2Cl_2 (deducted by ^{1}H NMR spectroscopy and elemental analysis). Single crystals suitable for X-Ray diffraction analysis were obtained by slow diffusion of *n*-pentane into a solution of the mixture in CH_2Cl_2 at -30°C.

^{1}H NMR (CD_2Cl_2, 300 K, in ppm): δ = 1.10 (6H, d, $^3J_{HH}$ = 6.8 Hz, H9/H17), 1.12 (6H, d, $^3J_{HH}$ = 6.8 Hz, H9/H17), 1.16 (6H, d, $^3J_{HH}$ = 6.8 Hz, H9/H17), 1.19 (6H, d, $^3J_{HH}$ = 6.8 Hz, H9/H17), 1.35 (6H, d, $^3J_{HH}$ = 7.0 Hz, H9/H17), 1.36 (6H, d, $^3J_{HH}$ = 6.9 Hz, H9/H17), 1.44 (6H, d, $^3J_{HH}$ = 6.8 Hz, H9/H17), 1.44 (6H, d, $^3J_{HH}$ = 6.4 Hz, H9/H17), 2.18 (4H, m, H7/16), 2.71 (2H, sept, $^3J_{HH}$ = 6.6

Hz, H7/16), 2.80 (2H, sept, ${}^{3}J_{HH}$ = 6.7 Hz, H7/16), 7.39 (8H, m, H5/14), 7.59 (2H, t, ${}^{3}J_{HH}$ = 7.8 Hz, H6/15), 7.66 (2H, t, ${}^{3}J_{HH}$ = 7.8 Hz, H6/15); **^{31}P{} NMR** (CD_2Cl_2, 300 K, in ppm): -212.6 (1P, dd, ${}^{1}J_{P(A)P(B)}$ = -251 Hz, ${}^{1}J_{P(A)P(X)}$ = -183 Hz, P_A), -206.1 (1P, dd, ${}^{1}J_{P(B)P(A)}$ = -251 Hz, ${}^{1}J_{P(B)P(X)}$ = -189 Hz, P_B), -133.1 (1P, t, ${}^{1}J_{P(X)P(A/B)}$ = -186 Hz, P_X); **Elemental analysis**: $C_{54}H_{68}Cl_9Ga_2N_4P_3$·0.2 $C_{27}H_{35}Cl_6GaN_2$·1 CH_2Cl_2: calcd.: N: 4.0, C: 47.00, H: 5.0; found: N: 3.8, C: 47.3; H: 4.6;

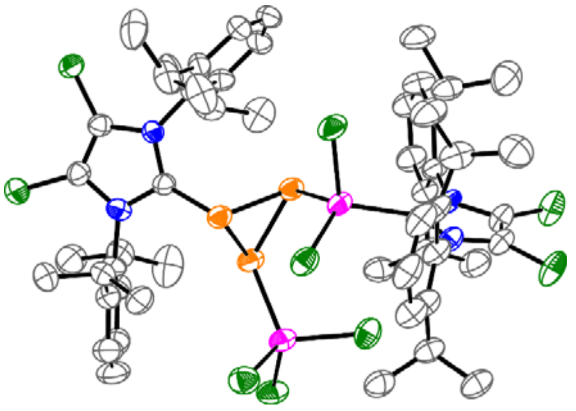

Fig. S25. Molecular structure of **24**; thermal ellipsoids are displayed at 50 % probability level; hydrogen atoms are omitted for clarity.

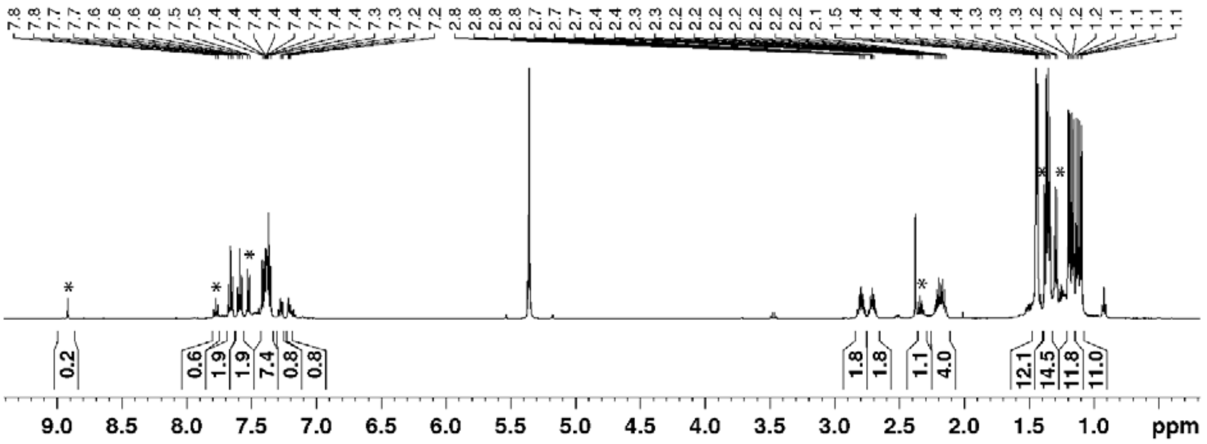

Fig. S26. ^{1}H NMR spectrum (CD_2Cl_2, 300 K) of **24**·0.2 **L**-H[$GaCl_4$](asterisks)·CH_2Cl_2.

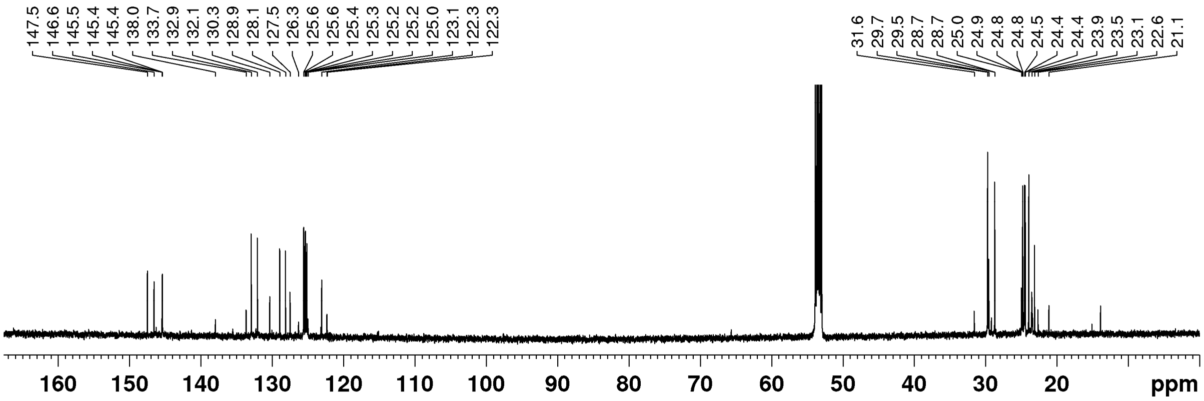

Fig. S27. $^{13}C\{^{1}H\}$ NMR spectrum (CD_2Cl_2, 300 K) of **24**·0.2 **L**-H[$GaCl_4$](asterisks)·CH_2Cl_2.

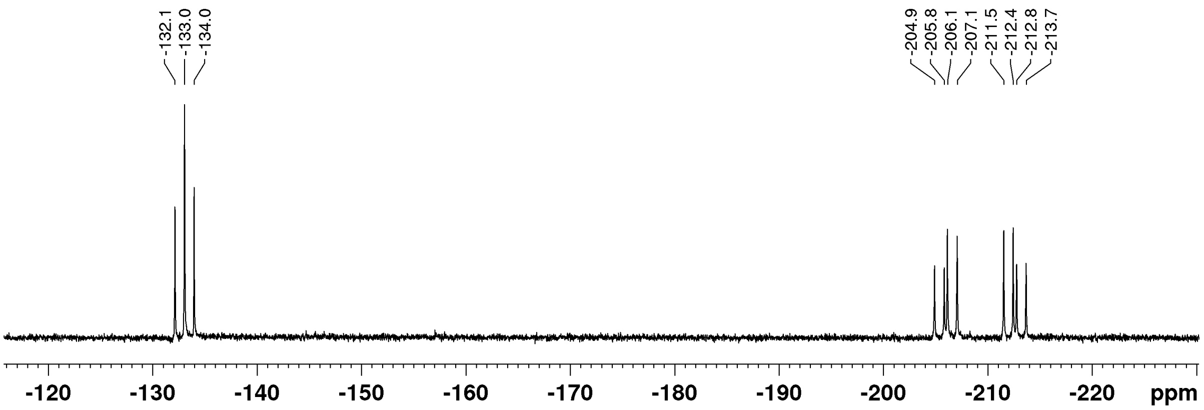

Fig. S28. ^{31}P NMR spectrum (CD_2Cl_2, 300 K) of **24**·0.2 **L**-H[$GaCl_4$](asterisks)·CH_2Cl_2.

Compound **24** proved to be instable in solution with slow decomposition to NHC-$GaCl_3$ and polymeric phosphorus species (cloudy solution).

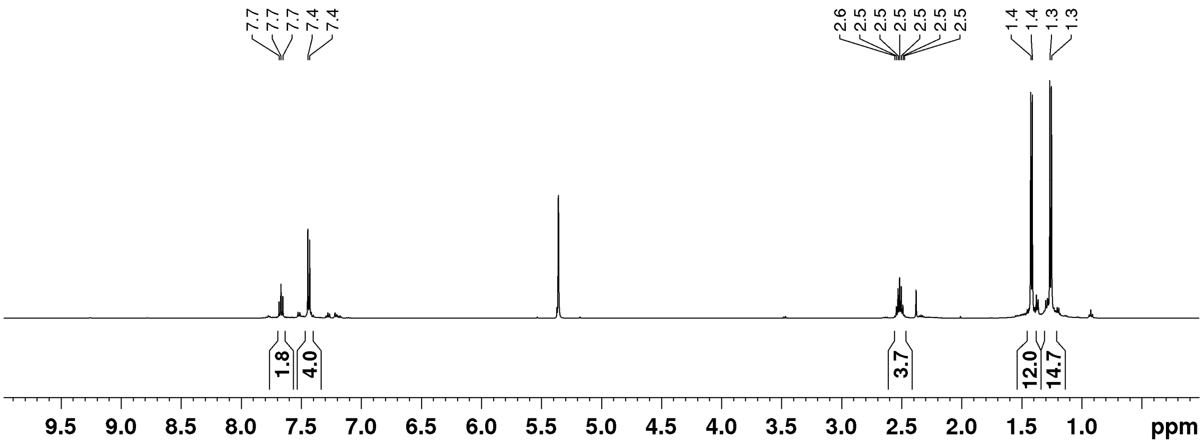

Fig. S29. ^{1}H NMR spectrum of the solution of **24** after 2 weeks under inert atmosphere. (CD_2Cl_2, 300 K)

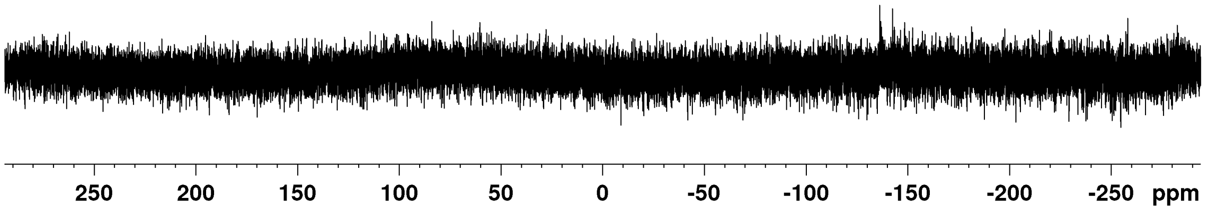

Fig. S30. ^{31}P NMR spectrum of the solution of **24** after 2 weeks under inert atmosphere. (CD_2Cl_2, 300 K)

2.13. Synthesis of $LP_3Pd(PPh_3)_2[GaCl_4]$ (25[$GaCl_4$])

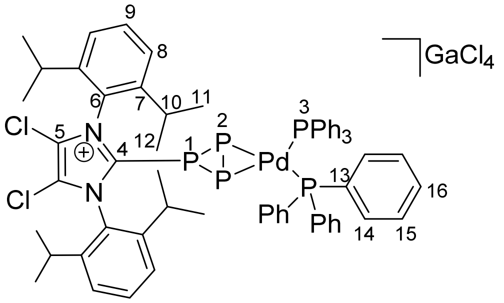

$Pd(PPh_3)_4$ (54 mg, 0.05 mmol) in 3 ml of CH_2Cl_2 was added to a solution of **10**[Ga_2Cl_7] (78 mg, 0.05 mmol, 1 eq) in 2 ml CH_2Cl_2 at room temperature. After 5 min all volatiles were removed *in vacuo*. The residue was suspended in fluorobenzene and filtered. *n*-Pentane was added to the filtrate to yield a brownish oil after stirring for 1 h. The oil was dissolved in dichloromethane and overlaid with n-pentane and stored at -30 °C to yield **25**[$GaCl_4$] (25 mg, 40 %) as brown crystals.

^{1}H NMR (CD_2Cl_2, 300 K, in ppm): 1.21 (12H, d; $^3J_{HH}$ = 6.7 Hz, H11) 1.29 (12H, d, $^3J_{HH}$ = 6.7 Hz, H12), 2.22 (4H, sep, $^3J_{HH}$ = 6.7 Hz, H10), 6.96 (12H, t, H15), 7.15 (12H, t, H14), 7.34 (6H, t, H16), 7.42 (4H, d, $^3J_{HH}$ = 7.7 Hz, H8), 7.75 (2H, t, $^3J_{HH}$ = 7.7 Hz, H9); **^{13}C{^{1}H} NMR** (CD_2Cl_2, 300K, in ppm): 24.3 (12C, C12), 24.5 (12C, C11), 29.8 (4C, C10), 121.7 (2C, $^3J_{PC}$ = 6 Hz, C5), 125.5 (4C, C8), 127.0 (2C, C6), 128.4 (12C, C14), 130.1 (6C, C16), 133.0 (2C, C9), 133.4 (6C, C13), 133.5 (12C, C15), 146.0 (4C, C7), 155.4 (1C, $^1J_{PC}$ = 190 Hz, C5); **^{31}P{^{1}H} NMR** (CD_2Cl_2, 300K, in ppm): -282.1 (1P, t,$^1J_{PP}$= -215 Hz, P1), -87.8 (2P, dt, $^1J_{PP}$= -215 Hz, $^2J_{PP}$= 30 Hz, P2), 24.2 (2P, t, $^2J_{PP}$= 30 Hz, P3); **IR** (ATR, 298 K, [cm^{-1}]): 3052 (w), 2964 (w), 2928 (w), 2868 (w), 1585 (w), 1564 (w), 1478 (w), 1465 (w), 1433 (m), 1387 (w), 1365 (w), 1327 (w), 1307 (w), 1202 (m), 1182 (m), 1148 (m), 1111 (w), 1093 (m), 1062 (w), 1027 (w), 998 (w), 934 (w), 911 (w), 847 (w), 804 (m), 762 (w), 742 (s), 692 (vs), 653 (w), 640 (w), 554 (w), 545 (w), 526 (s), 515 (vs), 504 (vs); **Elemental analysis:** $C_{63}H_{66}Cl_6GaN_2P_3Pd$: calcd.: N: 2.0, C: 54.3, H: 4.6; found: N: 2.0, C: 53.4; H: 4.6. Despite all efforts we could not obtain a sample that yielded sufficient accuracy on carbon-values, we attribute this to the possible formation of Pd-carbide species; **Melting point:** 195 °C (decomposition)

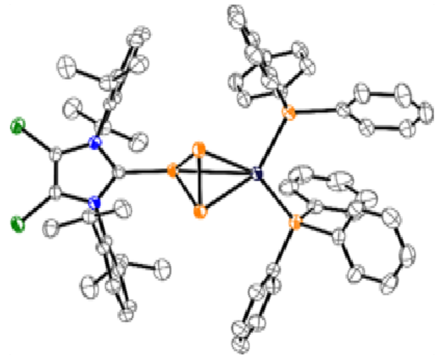

Fig. S31. Molecular structure of **25**$^+$ in **25**[$GaCl_4$]·CH_2Cl_2; thermal ellipsoids are displayed at 50 % probability level; hydrogen atoms, counter ion and solvate molecules are omitted for clarity.

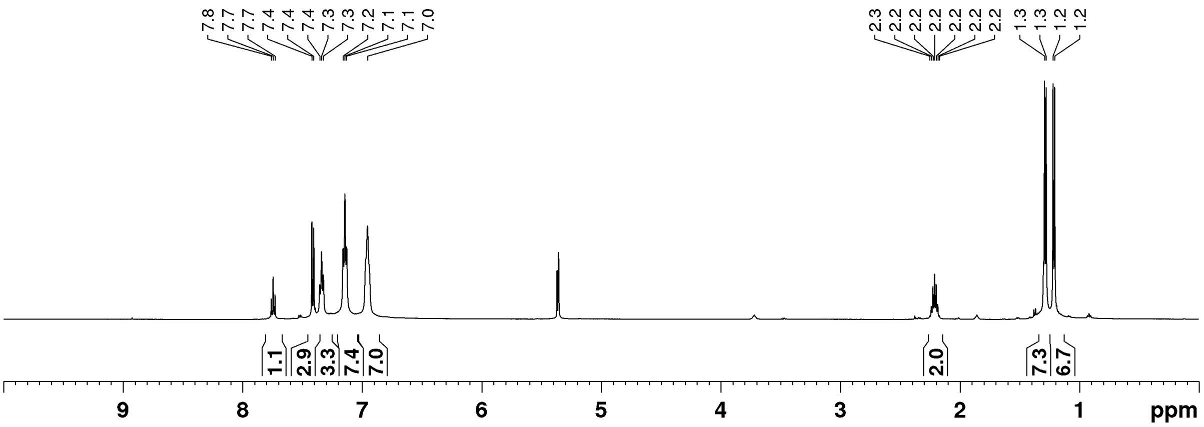

Fig. S32. ^{1}H NMR spectrum (CD_2Cl_2, 300 K) of **25**[$GaCl_4$].

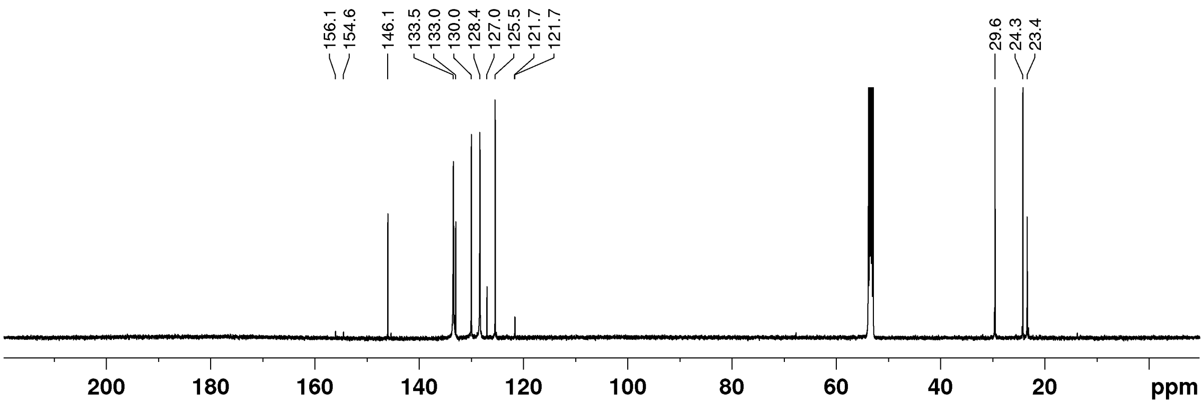

Fig. S33. $^{13}C\{^{1}H\}$ NMR spectrum (CD_2Cl_2, 300 K) of **25**[$GaCl_4$].

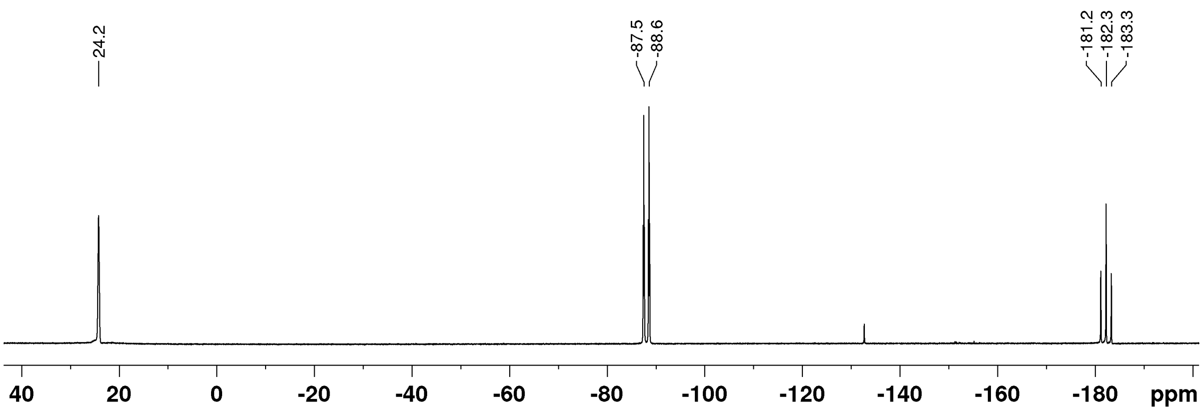

Fig. S34. ^{31}P NMR spectrum (CD_2Cl_2, 300 K) of **25**[$GaCl_4$].

2.14. Variable temperature NMR studies

The ^{31}P NMR spectrum of **10**$^+$ exhibits an AX_2 spin system at 300 K. A triplet resonance at δ = -119.5 ppm with $^1J(PP)$ = -274 Hz represents the central P atom as A part. The X part is a broad unresolved signal at δ = -7.2 ppm ($\square_{1/2}$ = 2800 Hz). Upon cooling to 243 K an AXY spin system resolves and the broad resonance separates into two sharp doublets. The Gutowsky–Holm equation[5] allows a rough estimation of $\Delta G^{\neq}$.

k_B Boltzmann constant $1.38064852 \cdot 10^{-23}\ m^2 \cdot kg \cdot s^{-2} \cdot K^{-1}$
h Planck constant $6.62607004 \cdot 10^{-34}\ m^2 \cdot kg \cdot s^{-1}$

$$k_{T_c} = \Delta\nu \cdot \frac{\pi}{\sqrt{2}}$$

$$\Delta G^{\neq} = RT \cdot \ln\left(\frac{k_B T}{k_{T_c} h}\right)$$

Coalescence occurs at 288 K and the signals are separated by 5077 Hz at low temperature. Therefore an energy barrier of 48 $kJ \cdot mol^{-1}$ can be attributed to the process.

2.15. Compounds possessing a P_3H_3 structural unit and their P NMR chemical shifts

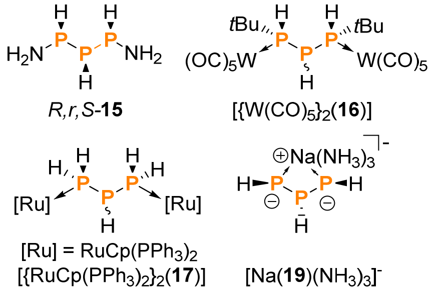

Fig. S35. Compounds possessing a P_3H_3 structural unit.

Table S1. Selected ^{31}P NMR shifts (in ppm), coupling constants (in Hz), P–P bond lengths (in Å) and P-P-P bond angles (in °) of compounds containing a H_3P_3 structural motif and of *rac*-**18**$^+$ for comparison.

	rac-**14**$^{2+}$	R,s,S-**14**$^{2+}$	*R,r,S*-15[22]	**16**[a]	**17**[25]	**17**[b]	**19**$^{2-}$[c]	*rac*-**18**$^+$	LiP_4H_4[26]
d($P_{central}$) [ppm]	-131.6	-140.5	-119	-90.7	-156.7	-148.6		-144.2	-251.7
d(P_{outer}) [ppm]	-109.7	-90.3	-152.5	-13.4	-173.2	-51		-90.3	-123
d(P'_{outer}) [ppm]	-105.5								
$^1J(P_cP_o)$ [Hz]	-146	-178	-238	-197	-147	-229		-223	-267
$^1J(P_cP'_o)$ [Hz]	-132					-213			
$^2J(P_oP'_o)$ [Hz]	282								
$^1J(P_cH)$ [Hz]	189	191	186	224		230		170	
$^1J(P_oH)$ [Hz]	229	181	127	316		350			
$^1J(P'_oH)$ [Hz]	229								
P1–P2 [Å]	2.21(1)			2.215(2)		2.197(2)	2.1977(6)		
P1–P2 [Å]	2.221(1)			2.238(2)		2.197(2)	2.1977(6)		
P1–P2–P2 [°]	93.85(5			103.25(7)	104.5[d]	99.2(1)	113.1(4)		

[a] in complex [{W(CO)5}2(**16**)][23]; [b] in complex [{RuCp(PPh3)2}2(**17**)][27]; [c] in [Na(NH3)5][Na(**19**)(NH3)3], no NMR data available[28]; [d] from Raman data

3. X-ray Diffraction Refinements

3.1. General remarks

Suitable single crystals were coated with Paratone-N oil or Fomblin Y25 PFPE oil, mounted using a glass fiber and frozen in the cold nitrogen stream. X-ray diffraction data were collected at low temperature as indicated in tables S1 and S2 on a Rigaku Oxford Diffraction SuperNova diffractometer using either Cu K_α radiation ($\lambda = 1.54184$ Å) generated by micro-focus sources. The data reduction and absorption correction was performed using CrysAlisPro[6], respectively. For further crystal and data collection details see **section 3.2**. Using Olex2[7], the structures were solved with SHELXT[8] by direct methods and refined with SHELXL[9] by least-square minimization against F^2 using first isotropic and later anisotropic thermal parameters for all non-hydrogen atoms. Hydrogen atoms were added to the structure models on calculated positions using the riding model. Images of the structures were produced with Olex2 software.

Two different solvates of **10**[Ga_2Cl_7] were identified to crystallize from a dichloromethane solution upon cooling and layering with *n*-pentane. A third crystal structure of the solvent free salt was determined of solvate crystals after heating during the attempt to determine the melting point. The structural parameters differentiate only slightly between the three structures. Due to disorder of the $Ga_2Cl_7^-$ anion and one imidazoliumyl-moiety in the solvent free structure SIMU, SADI and SAME restraints were applied to achieve a meaningful, converging model.

The solvent free structure and the solvate **10**[Ga_2Cl_7] · 2 CH_2Cl_2 have a closely related packing allowing the assumption that the former originates from the latter after evaporation of the solvent (see **Fig. S**). This process is accompanied by a shrinking of the unit cell volume of 6%. The solvate **10**[Ga_2Cl_7] · CH_2Cl_2 is built up differently and not related.

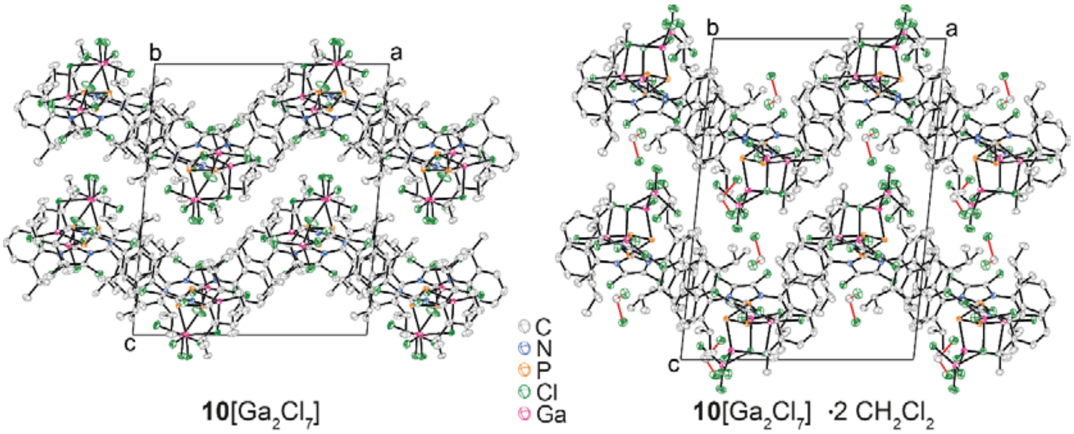

Fig. S36. Packing diagrams along [010] of the related structures **10**[Ga_2Cl_7] and the solvate **10**[Ga_2Cl_7]·2 CH_2Cl_2; the solvent molecules are highlighted by red bonds.

Single crystals of **25**[$GaCl_4$] were obtained by slow diffusion of *n*-pentane into a solution of **25**[$GaCl_4$] in fluorobenzene. Two of the solvate molecules, one phenyl group and the P_3Pd core are disordered over two positions. The minor component of the P_3Pd core represents less than 10%. To achieve a meaningful, converging model SIMU, SADI, FLAT and SAME restraints were applied.

3.2. Crystallographic data

Table S2. Crystallographic data of **8**$[Cl_3GaOTf]_2$, **9Cl**$[GaCl_4][Ga_2Cl_7]\cdot 3\ C_6H_4F_2$, and **10**$[Ga_2Cl_7]$.

	**8**$[Cl_3GaOTf]_2$_squeeze	**9Cl**$[GaCl_4][Ga_2Cl_7]$	**10**$[Ga_2Cl_7]$
Empirical formula	$C_{74}H_{80}Cl_{10}F_{12}Ga_2N_4O_6P_3S_2$	$C_{71.69}H_{82.25}Cl_{16}F_{5.37}Ga_3N_4P_3$	$C_{54}H_{68}Cl_{15}Ga_4N_4P_3$
Formula weight [g/mol]	2000.39	1971.18	1676.66
Temperature [K]	153(2)	100.0(2)	99.9(2)
Crystal system	triclinic	triclinic	monoclinic
Space group	P-1	*P*−1	$P2_1/c$
A [Å]	14.2206(5)	13.6871(2)	17.1010(2)
b [Å]	14.6422(5)	16.6177(2)	21.2518(3)
c [Å]	23.6877(8)	19.6162(3)	19.8695(3)
A [°]	102.888(2)	93.5990(10)	90
β [°]	90.442(2)	94.9660(10)	94.7050(13)
γ [°]	117.125(2)	90.0450(10)	90
Volume [$Å^3$]	4245.0(3)	4436.07(11)	7196.77(17)
Z	2	2	4
ρ_c [g cm^{-3}]	1.565	1.476	1.547
μ [mm^{-1}]	1.134	6.443	7.759
F(000)	2034.0	1997.0	3376.0
Crystal size [mm^3]	0.32 × 0.25 × 0.08	0.23 × 0.202 × 0.11	0.251 × 0.226 × 0.111
$\lambda_{XK□}$ [Å]	MoKα (λ = 0.71073)	CuKα (*λ* = 1.54184)	CuKα (λ = 1.54184)
θ_{min} [°]	1.78 to 55.76	5.328 to 154.862	5.186 to 153.028
Index ranges	-18 ≤ h ≤ 18, -19 ≤ k ≤ 19, -31 ≤ l ≤ 31	−17 ≤ h ≤ 17, −20 ≤ k ≤ 21, −20 ≤ l ≤ 24	-21 ≤ h ≤ 21, -26 ≤ k ≤ 26, -25 ≤ l ≤ 25
Reflections collected	53854	33930	46261
Independent reflections	20201 [R_{int} = 0.0285, R_{sigma} = 0.0389]	33930 [twin]	14977 [R_{int} = 0.0454, R_{sigma} = 0.0413]
Data / restraints / parameters	20201/2/822	33930/768/1238	14977/575/1016
GooF on F^2	1.092	1.094	1.022
Final R indices [I≥2σ(I)]	R_1 = 0.0564, wR_2 = 0.1822	R_1 = 0.0579 wR_2 = 0.1592	R_1 = 0.0735, wR_2 = 0.1809
Final R indices (all data)	R_1 = 0.0725, wR_2 = 0.1934	R_1 = 0.0665 wR_2 = 0.1644	R_1 = 0.0975, wR_2 = 0.2007
Largest diff. peak and hole [e $Å^3$]	1.92/-1.86	1.88/−1.00	3.11/-1.09
CCDC	2385681	2449066	2385684

Table S3. Crystallographic data of **10**[Ga_2Cl_7]·2 CH_2Cl_2, **10**[Ga_2Cl_7]·CH_2Cl_2, **12**, and *rac*-**14**[$(Cl_3Ga)_2OH$].

	**10**[Ga_2Cl_7]·CH_2Cl_2	**10**[Ga_2Cl_7]·2 CH_2Cl_2	**12**
Empirical formula	$C_{55}H_{70}Cl_{17}Ga_4N_4P_3$	$C_{56}H_{72}Cl_{19}Ga_4N_4P_3$	$C_{27}H_{34}Cl_7Ga_2N_2P$
Formula weight [g/mol]	1761.59	1846.51	805.12
Temperature [K]	100.0	99.98(18)	100.01(10)
Crystal system	triclinic	monoclinic	triclinic
Space group	P-1	$P2_1/c$	P-1
A [Å]	11.06831(13)	16.24760(14)	9.25771(9)
b [Å]	16.5810(3)	21.01748(16)	18.25509(18)
c [Å]	20.4203(2)	22.5448(2)	20.21174(15)
Α [°]	78.7424(11)	90	84.3049(7)
β [°]	82.1019(9)	95.7862(8)	83.9454(7)
γ [°]	87.9538(11)	90	86.4314(8)
Volume [$Å^3$]	3640.50(8)	7659.46(11)	3375.34(5)
Z	2	4	4
ρ_c [g cm^{-3}]	1.607	1.601	1.584
μ [mm^{-1}]	8.359	8.603	7.672
F(000)	1772.0	3712.0	1624.0
Crystal size [mm^3]	0.229 × 0.127 × 0.016	0.252 × 0.223 × 0.17	0.415 × 0.237 × 0.036
$\lambda_{XK□}$ [Å]	CuKα (λ = 1.54184)	CuKα (λ = 1.54184)	CuKα (λ = 1.54184)
θ_{min} [°]	5.434 to 153.73	5.468 to 153.452	6.264 to 153.358
Index ranges	$-13 \leq h \leq 12$, $-20 \leq k \leq 20$, $-25 \leq l \leq 24$	$-20 \leq h \leq 16$, $-24 \leq k \leq 26$, $-28 \leq l \leq 27$	$-11 \leq h \leq 11$, $-22 \leq k \leq 23$, $-25 \leq l \leq 24$
Reflections collected	38935	93248	82943
Independent reflections	15121 [R_{int} = 0.0411, R_{sigma} = 0.0466]	16069 [R_{int} = 0.0324, R_{sigma} = 0.0190]	14101 [R_{int} = 0.0390, R_{sigma} = 0.0199]
Data / restraints / parameters	15121/0/764	16069/0/791	14101/2/762
GooF on F^2	1.040	1.073	1.095
Final R indices [I≥2σ(I)]	R_1 = 0.0374, wR_2 = 0.0972	R_1 = 0.0334, wR_2 = 0.0881	R_1 = 0.0418, wR_2 = 0.1128
Final R indices (all data)	R_1 = 0.0448, wR_2 = 0.1029	R_1 = 0.0369, wR_2 = 0.0918	R_1 = 0.0420, wR_2 = 0.1130
Largest diff. peak and hole [e $Å^3$]	0.82/-1.47	1.33/-1.09	1.32/-1.03
CCDC	2385686	2385688	2385690

Table S4. Crystallographic data of *rac*-**14**[$(Cl_3Ga)_2OH$] **22**, and **22’**·CH_2Cl_2 *n*-hexane.

	rac-**14**[$(Cl_3Ga)_2OH$]	**22**	**22’**·CH_2Cl_2·*n*-hexane
Empirical formula	$C_{54}H_{73}Cl_{16}Ga_4N_4O_2P_3$	$C_{27}H_{34}Cl_7Ga_2N_2P_3$	$C_{31}H_{44}Cl_8Ga_2N_2OP_3$
Formula weight [g/mol]	1749.15	867.06	976.63
Temperature [K]	100.00(16)	100.01(10)	99.99(10)
Crystal system	triclinic	monoclinic	monoclinic
Space group	P-1	$P2_1/n$	$P2_1/c$
A [Å]	10.58550(10)	10.4194(2)	10.44880(10)
b [Å]	12.2725(2)	21.0063(3)	21.7784(3)
c [Å]	15.17260(10)	17.2261(2)	18.8820(3)
A [°]	105.0510(10)	90	90
β [°]	99.3760(10)	102.2200(10)	98.9810(10)
γ [°]	90.8640(10)	90	90
Volume [Å^3]	1874.62(4)	3684.90(10)	4244.07(10)
Z	1	4	4
ρ_c [g cm^{-3}]	1.549	1.563	1.528
μ [mm^{-1}]	7.812	7.869	7.484
F(000)	882.0	1744.0	1980.0
Crystal size [mm^3]	0.275 × 0.208 × 0.03	0.174 × 0.151 × 0.032	0.188 × 0.042 × 0.036
$\lambda_{XK\square}$ [Å]	CuKα (λ = 1.54184)	Cu Kα (λ = 1.54184)	Cu Kα (λ = 1.54184)
θ_{min} [°]	6.124 to 153.234	6.728 to 153.51	6.24 to 153.752
Index ranges	-13 ≤ h ≤ 12, -15 ≤ k ≤ 15, -19 ≤ l ≤ 19	-10 ≤ h ≤ 13, -26 ≤ k ≤ 26, -21 ≤ l ≤ 15	-13 ≤ h ≤ 11, -25 ≤ k ≤ 27, -23 ≤ l ≤ 23
Reflections collected	41589	26760	52102
Independent reflections	7826 [R_{int} = 0.0336, R_{sigma} = 0.0170]	7628 [R_{int} = 0.0708, R_{sigma} = 0.0578]	8890 [R_{int} = 0.0522, R_{sigma} = 0.0329]
Data / restraints / parameters	7826/73/519	7628/0/408	8890/152/592
GooF on F^2	1.027	0.969	1.036
Final R indices [I≥2σ(I)]	R_1 = 0.0274, wR_2 = 0.0746	R_1 = 0.0489, wR_2 = 0.1213	R_1 = 0.0499, wR_2 = 0.1370
Final R indices (all data)	R_1 = 0.0280, wR_2 = 0.0751	R_1 = 0.0674, wR_2 = 0.1278	R_1 = 0.0593, wR_2 = 0.1460
Largest diff. peak and hole [e Å^3]	0.73/-0.55	0.77/-1.14	0.92/-0.53
CCDC	2385683	2385693	2385685

Table S5. Crystallographic data of $HNEt_3$[**23**]$\cdot C_6H_5F$, **24**, and **25**[$GaCl_4$]$\cdot CH_2Cl_2$.

	$HNEt_3$[**23**]$\cdot C_6H_5F$	**24**	**25**[$GaCl_4$]$\cdot CH_2Cl_2$
Empirical formula	$C_{39}H_{55}Cl_8FGa_2N_3P_3$	$C_{54}H_{68}Cl_9Ga_2N_4P_3$	$C_{64}H_{66}Cl_8GaN_2P_5Pd$
Formula weight [g/mol]	1100.81	1324.52	1477.75
Temperature [K]	100.0(6)	100.00(10)	100.00(10)
Crystal system	monoclinic	orthorhombic	triclinic
Space group	$P2_1/c$	$P2_12_12_1$	P-1
A [Å]	10.48038(9)	12.3341(2)	10.45460(10)
b [Å]	21.3418(2)	20.5572(2)	18.0673(2)
c [Å]	22.8449(2)	28.8311(7)	18.9145(3)
A [°]	90	90	86.6960(10)
β [°]	99.8982(8)	90	88.2990(10)
γ [°]	90	90	74.8970(10)
Volume [$Å^3$]	5033.67(8)	7310.3(2)	3443.16(8)
Z	4	4	2
ρ_c [g cm^{-3}]	1.453	1.203	1.425
μ [mm^{-1}]	6.397	4.803	6.846
F(000)	2248.0	2720.0	1504.0
Crystal size [mm^3]	0.169 × 0.114 × 0.052	0.13 × 0.1 × 0.02	0.283 × 0.205 × 0.025
$\lambda_{XK\square}$ [Å]	CuKα (λ = 1.54184)	Cu Kα (λ = 1.54184)	Cu Kα (λ = 1.54184)
θ_{min} [°]	8.564 to 153.82	5.28 to 153.812	4.68 to 153.48
Index ranges	$-11 \leq h \leq 13, -26 \leq k \leq 25, -27 \leq l \leq 28$	$-15 \leq h \leq 15, -23 \leq k \leq 25, -35 \leq l \leq 35$	$-13 \leq h \leq 9, -22 \leq k \leq 22, -23 \leq l \leq 23$
Reflections collected	30613	70442	40574
Independent reflections	10496 [R_{int} = 0.0305, R_{sigma} = 0.0319]	15021 [R_{int} = 0.0662, R_{sigma} = 0.0591]	14289 [R_{int} = 0.0399, R_{sigma} = 0.0390]
Data / restraints / parameters	10496/295/678	15021/0/665	14289/26/795
GooF on F^2	1.025	1.078	1.026
Final R indices [I≥2σ(I)]	R_1 = 0.0468, wR_2 = 0.1218	R_1 = 0.0504, wR_2 = 0.1225	R_1 = 0.0351, wR_2 = 0.0899
Final R indices (all data)	R_1 = 0.0515, wR_2 = 0.1264	R_1 = 0.0684, wR_2 = 0.1335	R_1 = 0.0386, wR_2 = 0.0927
Largest diff. peak and hole [e $Å^3$]	1.61/-0.83	0.37/-0.73	0.72/-1.08
Flack Parameter	2385680	0.009(8)	--
CCDC		2385689	2385687

Table S6. Crystallographic data of Et_3NGaCl_3 , H-DMAP[$GaCl_4$], and **L**-$GaCl_3 \cdot CH_2Cl_2$.

	Et_3NGaCl_3	H-DMAP[$GaCl_4$]	**L**-$GaCl_3 \cdot CH_2Cl_2$
Empirical formula	$C_6H_{15}NCl_3Ga$	$C_7H_{11}N_2Cl_4Ga$	$C_{28}H_{36}Cl_7GaN_2$
Formula weight [g/mol]	277.26	334.70	718.46
Temperature [K]	100.00(10)	100~~.01~~(1~~0~~)	100~~.0~~(1~~3~~)
Crystal system	orthorhombic	monoclinic	monoclinic
Space group	$Pna2_1$	$P2_1/n$	$P2_1/m$
A [Å]	13.20270(10)	9.0511(2)	9.8124(4)
b [Å]	7.09500(10)	13.9349(3)	15.9107(8)
c [Å]	11.91280(10)	10.1955(3)	10.9348(5)
A [°]	90	90	90
β [°]	90	99.172(3)	103.412(5)
γ [°]	90	90	90
Volume [$Å^3$]	1115.91(2)	1269.48(6)	1660.60(14)
Z	4	4	2
ρ_c [g cm^{-3}]	1.650	1.751	1.437
μ [mm^{-1}]	9.546	10.440	6.474
F(000)	560.0	664.0	736.0
Crystal size [mm^3]	0.23 × 0.15 × 0.08	0.26 × 0.17 × 0.05	0.223 × 0.059 × 0.022
$\lambda_{XK□}$ [Å]	Cu Kα (λ = 1.54184)	Cu Kα (λ = 1.54184)	CuKα (λ = 1.54184)
θ_{min} [°]	13.412 to 153.008	10.844 to 153.154	8.312 to 152.848
Index ranges	$-16 \le h \le 16$, $-8 \le k \le 7$, $-15 \le l \le 14$	$-10 \le h \le 11$, $-12 \le k \le 17$, $-12 \le l \le 10$	$-11 \le h \le 12$, $-18 \le k \le 20$, $-13 \le l \le 13$
Reflections collected	10282	7869	10961
Independent reflections	2287 [R_{int} = 0.0245, R_{sigma} = 0.0183]	2583 [R_{int} = 0.0275, R_{sigma} = 0.0257]	3574 [R_{int} = 0.0391, R_{sigma} = 0.0340]
Data / restraints / parameters	2287/1/131	2583/0/129	3574/0/185
GooF on F^2	1.059	1.057	1.084
Final R indices [$I \ge 2\sigma(I)$]	R_1 = 0.0162, wR_2 = 0.0412	R_1 = 0.0306, wR_2 = 0.0814	R_1 = 0.0600, wR_2 = 0.1519
Final R indices (all data)	R_1 = 0.0164, wR_2 = 0.0413	R_1 = 0.0357, wR_2 = 0.0858	R_1 = 0.0644, wR_2 = 0.1550
Largest diff. peak and hole [e $Å^3$]	0.17/-0.29	0.45/-0.62	1.05/-1.25
Flack Parameter	0.006(12)	--	--
CCDC	2385694	2385692	2385682

Table S7. Crystallographic data of **L-**H[$GaCl_4$].

	**L**-H[$GaCl_4$]
Empirical formula	$C_{13.5}H_{17.5}Cl_3Ga_{0.5}N$
Formula weight [g/mol]	334.99
Temperature [K]	153(1)
Crystal system	orthorhombic
Space group	$Pmn2_1$
A [Å]	18.4809(12)
b [Å]	9.3455(6)
c [Å]	9.4345(6)
A [°]	90.00
β [°]	90.00
γ [°]	90.00
Volume [$Å^3$]	1629.46(18)
Z	4
ρ_c [g cm^{-3}]	1.366
μ [mm^{-1}]	1.355
F(000)	688.0
Crystal size [mm^3]	0.13 × 0.07 × 0.03
$\lambda_{XK\square}$ [Å]	MoKα (λ = 0.71073)
θ_{min} [°]	4.36 to 55.74
Index ranges	-24 ≤ h ≤ 24, -12 ≤ k ≤ 12, -12 ≤ l ≤ 12
Reflections collected	15928
Independent reflections	3998 [R_{int} = 0.0214, R_{sigma} = 0.0193]
Data / restraints / parameters	3998/1/174
GooF on F^2	1.181
Final R indices [I≥2σ(I)]	R_1 = 0.0508, wR_2 = 0.1438
Final R indices (all data)	R_1 = 0.0519, wR_2 = 0.1442
Largest diff. peak and hole [e $Å^3$]	1.01/-0.86
Flack Parameter	0.06(2)
CCDC	2385691

4. Computational details

All calculations were carried out in the gas phase with the ORCA[10,11] program package (program version 4.0.1.2) and Gaussian16[12] (Rev. A.03). Geometry optimizations were performed at the PBE0[13]-D3BJ[14]/def2TZVP (28 electron ECP on Pd center) level of theory including the atom-pairwise dispersion correction with the Becke-Johnson damping scheme. Optimized geometries were checked for the absence negative modes with frequency analysis to assure an energetical minimum structure. NBO calculations were performed using the NBO 6.0 software[15]. Natural Resonance Theory (NRT) was limited to the atoms Pd(1), P(2), P(3), P(4), P(5), P(6) and C(11). AIM calculations were performed and visualized using the AIMall Professional software suite[16]. The bonding situation further analyzed using an energy decomposition analyses (EDA) and the natural orbitals for chemical valence (NOCs) were calculated using the Extended Transition State (ETS) method by Ziegler[17].
Iso-propyl groups of cation **25**$^+$ were substituted by methyl groups and the triflate counterion was not included in the theoretical investigation. The hypothetical **P$_3$**$^+$ and **P$_3$Pd**$^+$ fragments were optimized in D_{3h} and C_{3v} symmetry, respectively.

Table S8. Cartesian coordinates of **25**$^+$ (PBE0-D3BJ/def2TZVP).

Atom	x	y	z
Pd	2.60638	4.19078	3.81718
P1	5.53673	4.03442	3.84461
P2	4.14722	3.51285	5.46712
P3	4.29165	5.54612	4.83589
P4	1.51659	2.14571	3.59869
P5	1.65029	5.71563	2.34968
Cl	9.99068	2.08984	6.44153
Cl	10.47076	5.53170	5.91734
N	7.71024	2.92319	5.27844
N	8.00410	5.03825	4.95288
C	7.12836	4.03379	4.78598
C	8.96377	3.23092	5.76766
C	9.14993	4.56191	5.56340
C	7.09367	1.62753	5.29745
C	6.46958	1.21399	6.47316
C	5.80562	-0.00578	6.43971
H	5.28510	-0.34587	7.32702
C	5.78747	-0.77248	5.28746
H	5.25539	-1.71615	5.27879
C	6.44727	-0.34586	4.14761
H	6.43931	-0.95884	3.25418

C	7.11846	0.87057	4.12839
C	6.50593	2.04371	7.71599
H	6.23666	3.08286	7.51347
C	7.82293	1.34885	2.89816
H	8.83252	1.70284	3.11960
C	7.79596	6.39945	4.55283
C	7.37268	7.30834	5.52002
C	7.19869	8.62533	5.11442
H	6.86600	9.35972	5.83825
C	7.44650	9.00181	3.80528
H	7.31629	10.03694	3.51170
C	7.86269	8.06962	2.86948
H	8.05750	8.37288	1.84723
C	8.04886	6.73963	3.22660
C	7.10589	6.88196	6.92765
H	8.00678	6.49319	7.40986
C	8.49870	5.72024	2.22850
H	7.69959	5.00582	2.00645
C	1.59874	1.13417	5.10885
C	1.86269	-0.23090	5.07546
H	2.03215	-0.72738	4.12770
C	1.91734	-0.95931	6.25519
H	2.12466	-2.02272	6.21991
C	1.69990	-0.33389	7.47320
H	1.73766	-0.90638	8.39270
C	1.43225	1.02809	7.51293
H	1.26290	1.52293	8.46201
C	1.39102	1.75878	6.33768
H	1.20354	2.82732	6.37050
C	-0.24513	2.11891	3.15955
C	-1.21899	1.64013	4.02964
H	-0.93545	1.23049	4.99119
C	-2.55823	1.67770	3.66614
H	-3.30935	1.30240	4.35125
C	-2.93184	2.18207	2.43156
H	-3.97748	2.20760	2.14840
C	-1.96316	2.65665	1.55680
H	-2.24883	3.05828	0.59228
C	-0.63003	2.63483	1.92153
H	0.11707	3.01864	1.23594
C	2.29917	1.10317	2.32903
C	3.67861	1.19649	2.15318
H	4.25330	1.88598	2.76232

C	4.31595	0.42299	1.19744
H	5.38733	0.51310	1.06133
C	3.58362	-0.45118	0.40672
H	4.08068	-1.04979	-0.34779
C	2.21104	-0.55355	0.58077
H	1.63431	-1.23586	-0.03252
C	1.56955	0.21867	1.53742
H	0.49676	0.13837	1.66334
C	2.16759	7.44405	2.58251
C	1.29105	8.43175	3.01564
H	0.25005	8.19276	3.19609
C	1.74475	9.72972	3.21270
H	1.05356	10.49286	3.55069
C	3.07006	10.04945	2.96968
H	3.41970	11.06467	3.11652
C	3.94969	9.06485	2.53657
H	4.98857	9.30513	2.34793
C	3.50525	7.76871	2.35303
H	4.19922	7.00228	2.02302
C	-0.16012	5.79711	2.44075
C	-0.95627	6.18684	1.36781
H	-0.50417	6.42876	0.41362
C	-2.33296	6.25275	1.51218
H	-2.94775	6.55291	0.67161
C	-2.92320	5.93316	2.72659
H	-4.00040	5.98110	2.83465
C	-2.13530	5.54272	3.79918
H	-2.59341	5.27935	4.74489
C	-0.75995	5.47081	3.65479
H	-0.13959	5.14435	4.48340
C	2.05931	5.41175	0.60566
C	1.99035	6.42119	-0.35467
H	1.72537	7.43075	-0.06171
C	2.27880	6.14177	-1.68049
H	2.22077	6.93105	-2.42087
C	2.64662	4.85747	-2.06007
H	2.87327	4.64422	-3.09818
C	2.73688	3.85379	-1.10777
H	3.03896	2.85223	-1.39126
C	2.44963	4.13253	0.21944
H	2.54532	3.35321	0.96532
H	7.90544	0.54347	2.16903
H	7.28015	2.17534	2.42768

H	5.80212	1.65672	8.45153
H	7.50218	2.03566	8.16753
H	6.74715	7.72274	7.51947
H	6.34250	6.09928	6.96141
H	8.78353	6.20056	1.29337
H	9.35880	5.15069	2.58945

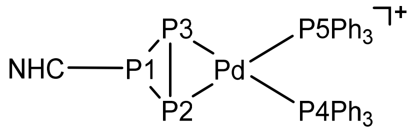

Scheme S 1: Numbering scheme of compound **25**$^{+}$.

Table S9. Cartesian coordinates of $\mathbf{P_3}^{+}$ (PBE0-D3BJ/def2TZVP).

Atom	x	y	z
P	1.04565	-0.60370	0.00000
P	-1.04565	-0.60370	0.00000
P	0.00000	1.20741	0.00000

Table S10. Cartesian coordinates of $\mathbf{PdP_3}^{+}$ (PBE0-D3BJ/def2TZVP).

Atom	x	y	z
Pd	0.00000	0.00000	1.01312
P	0.00000	1.23138	-1.03564
P	-1.06641	-0.61569	-1.03564
P	1.06641	-0.61569	-1.03564

Table S11. Frontier orbitals (isovalue = 0.04) and energies of compound **25**$^{+}$ (PBE0-D3BJ/def2TZVP).

Orbital label	Orbital plot	Orbital energy in H
HOMO-4	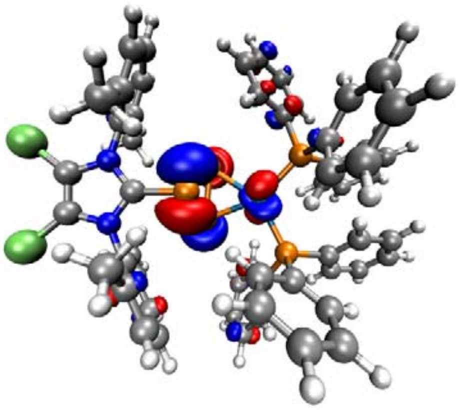	-0.33240
HOMO-3	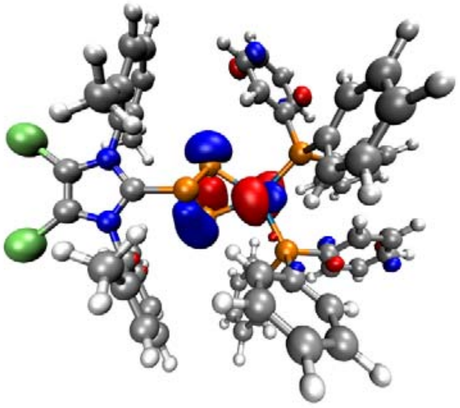	-0.32820
HOMO-2	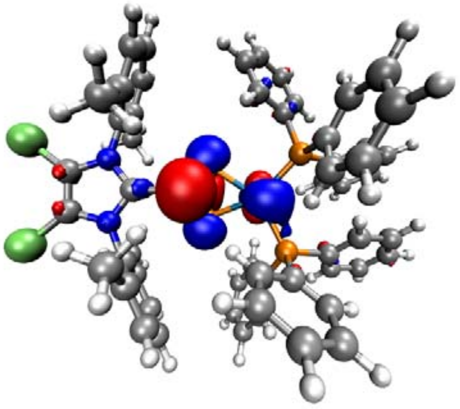	-0.31585

HOMO-1 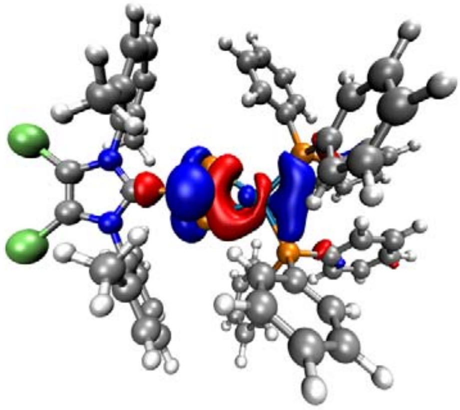 -0.31116

HOMO 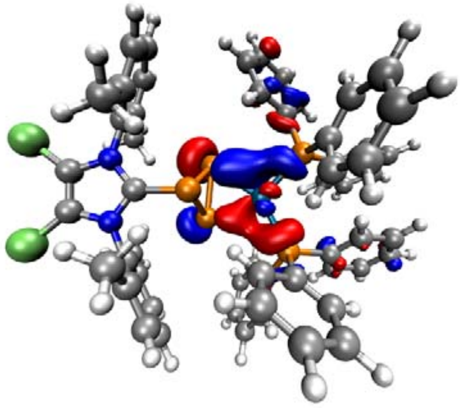 -0.29943

LUMO 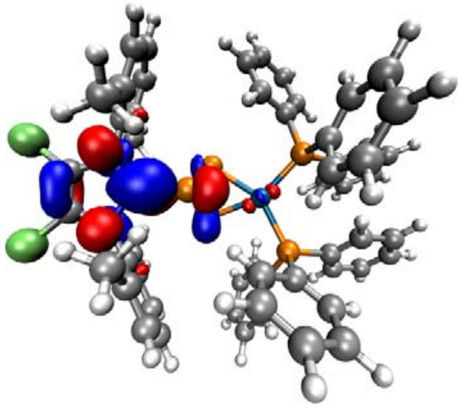 -0.13705

LUMO+1 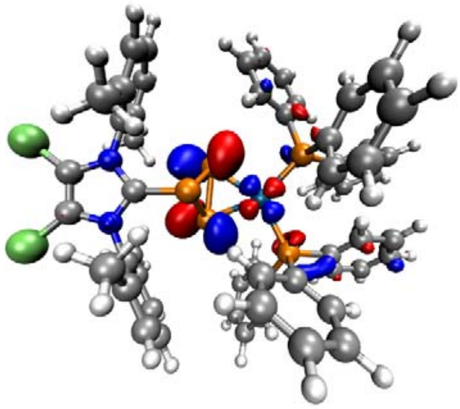 -0.13008

LUMO+2 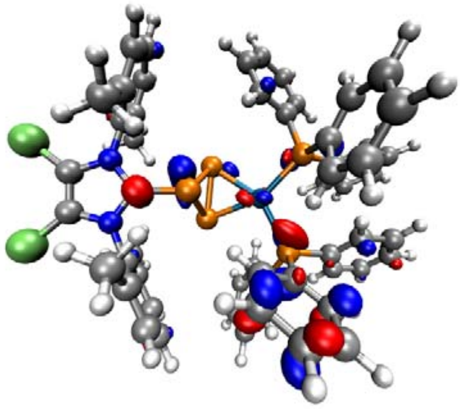 -0.11788

LUMO+3 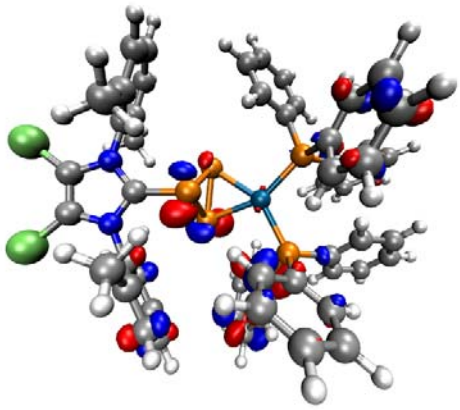 -0.11365

LUMO+4	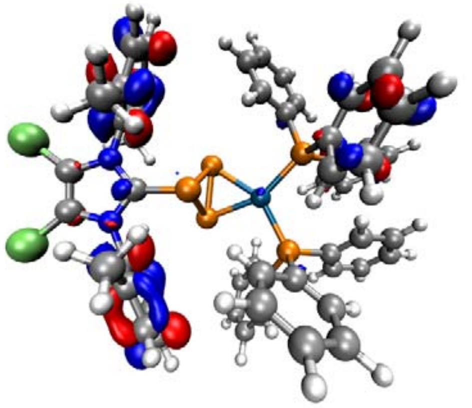	-0.11204

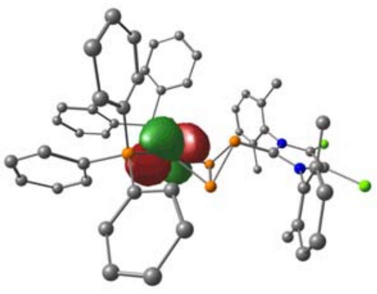

LMO 81 (0.03)

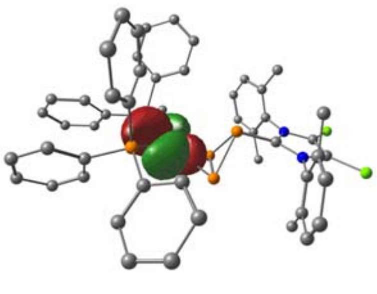

LMO 82 (0.03)

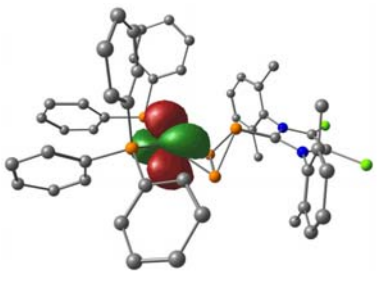

LMO 83 (0.03)

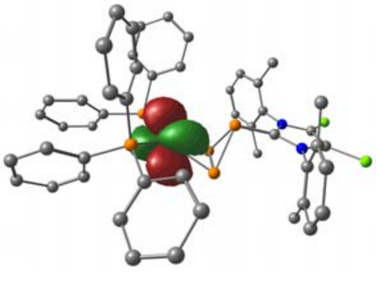

LMO 84 (0.03)

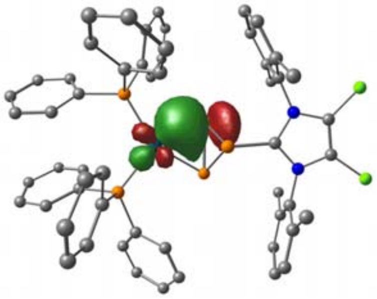

LMO 109 (0.05)

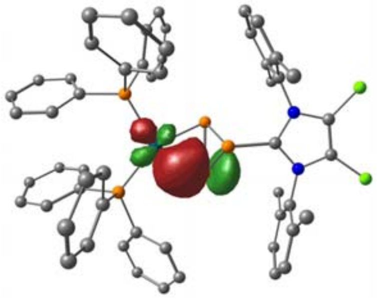

LMO 111 (0.05)

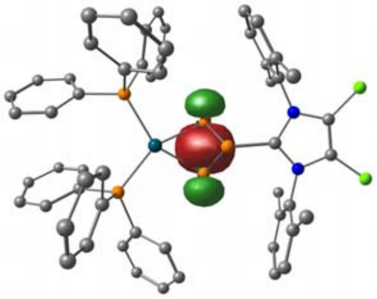

LMO 113 (0.05)

Fig. S37. Selected localized orbitals of compound **25**$^{+}$ (PBE0-D3BJ/def2TZVP). Respective isovalues are given in parenthesis.

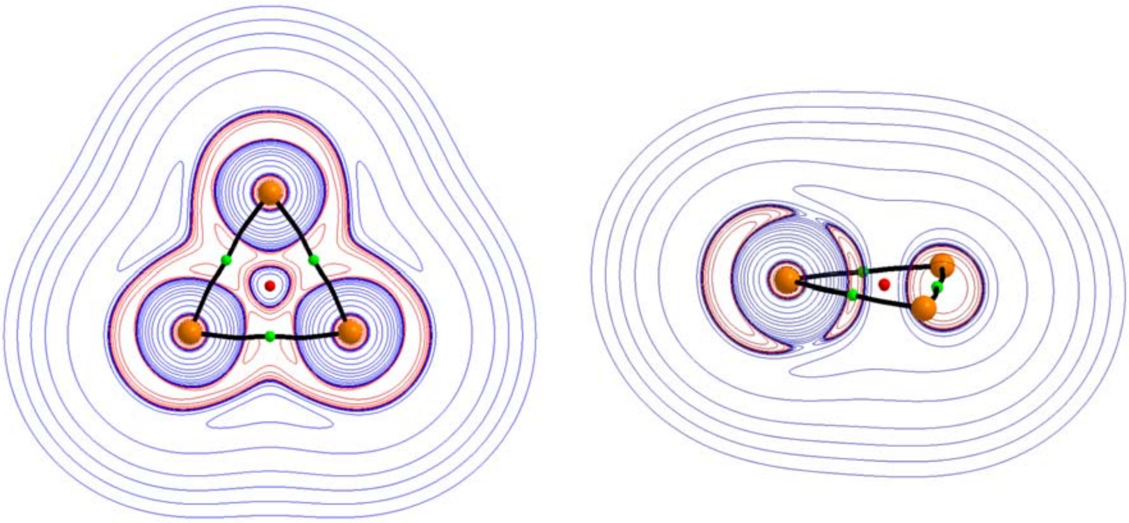

Fig. S38. Visualization of $\nabla2\rho$ of compound P_3^+ (PBE0-D3BJ/def2TZVP); left: P-P-P plane, right: plane perpendicular to molecular plane. Charge accumulations ($\nabla2\rho(r) < 0$) are printed in red, charge depletion ($\nabla2\rho(r) > 0$) in blue. (green: BCP, red: RCP).

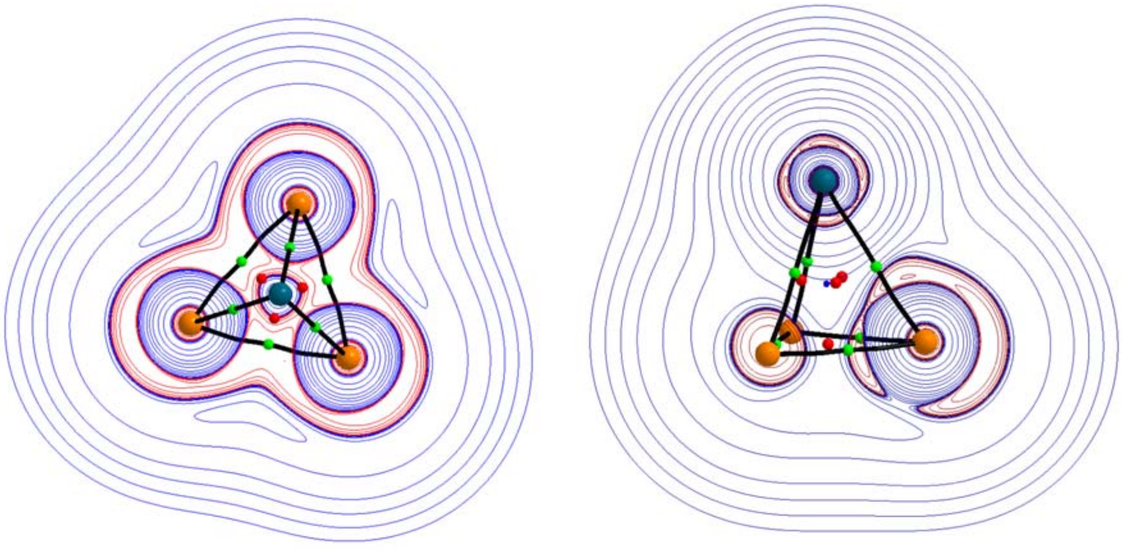

Fig. S39. Visualization of $\nabla2\rho$ of compound $[PdP_3]^+$ (PBE0-D3BJ/def2TZVP), left: P-P-P plane, right: Pd-P-BCPP–P plane. Charge accumulations ($\nabla2\rho(r) < 0$) are printed in red, charge depletion ($\nabla2\rho(r) > 0$) in blue. (green: BCP, red: RCP, blue: CCP).

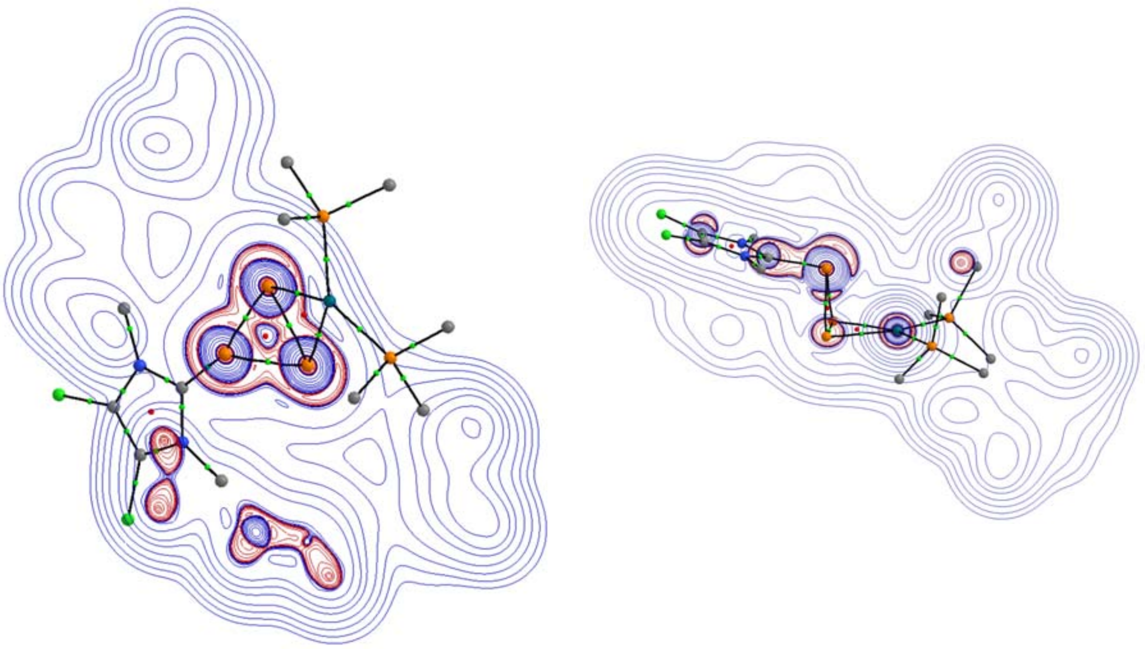

Fig. S40. Visualization of ∇2ρ of compound **25**$^{+}$ (PBE0-D3BJ/def2TZVP); left: P-P-P plane, right: P-BCPP–P-Pd plane. Charge accumulations (∇2ρ(r) < 0) are printed in red, charge depletion (∇2ρ(r) > 0) in blue. 2,6-Dimethylphenyl and phenyl moieties were omitted for clarity (green: BCP, red: RCP, blue: CCP).

Table S12. Sum of the Wiberg Bond Indices (WBI) and natural charges selected atoms in compound **25^+**, **$[PdP_3]^+$** and **P_3^+** (PBE0-D3BJ/def2TZVP).

Atom	**25^+** q in e	**25^+** WBI	**PdP_3^+** q in e	**PdP_3^+** WBI	**P_3^+** q in e	**P_3^+** WBI
Pd	0.035	1.66	0.405	1.43	-	-
P1	0.133	3.08	0.198	3.01	0.333	-
P2	-0.107	3.04	0.198	3.01	0.333	-
P3	-0.093	3.02	0.198	3.01	0.333	-
P4	0.992	3.57	-	-	-	-
P5	0.988	3.57	-	-	-	-
C11	0.197	3.78	-	-	-	-

Table S13. Selected NBOs of **P_3^+** (PBE0-D3BJ/def2TZVP).

Atom 1	Atom 2	Atom 3	Type	Occupation	Hybrid 1 (coeff.)	Hybrid 2 (coeff.)	Hybrid 3 (coeff.)
P1	-	-	LP*	2.00	$sp^{0.20}$ (100%)	-	-
P1	P2	-	BD(1)*	1.99	$sp^{10.74}$ (50%)	$sp^{10.74}$ (50%)	-
P1	P2	P3	3c2e	2.00	p (33.3%)	p (33.3%)	p (33.3%)

*Same orbital for other P atoms.

Table S14. Selected NBOs and orbital interactions of **$[PdP_3]^+$** (PBE0-D3BJ/def2TZVP).

Atom 1	Atom 2	Type	Occ.	Hybrid 1 (coeff.)	Hybrid 2 (coeff.)	Acceptor	$E^{(2)}$ in kcal/mol
Pd	-	LP[a]	2.00	d (100%)	-	-	-
P1	-	LP[b]	1.99	$sp^{0.21}$ (100%)	-	-	-
P3	-	LP(2)	0.80	p (100%)	-	BD*(1) Pd-P1[e]	170.00
Pd	P1	BD(1)[c]	1.98	d (79.5%)	$sp^{62.96}d^{1.88}$ (20.5%)	BD*(1) Pd-P2[f]	4.32
P1	P2	BD(1)[d]	1.99	$sp^{11.35}$ (50%)	$sp^{11.35}$ (50%)	-	-

[a] 3 d-type orbitals in total, [b] on all P atoms, [c] only for P1 and P2, [d] averaged values over all P–P bonds, [e] also for other BD* Pd-P2, [f] and vice versa

To further explore the bonding in compound **25^+**, we utilized restricted natural resonance theory (NRT) to identify the dominant Lewis structures (Fehler! Verweisquelle konnte nicht gefunden werden. and **Fig. S38**).[18] The most prominent resonance structures feature a bonding phenomenon referred to as "long-bonding". This form of 3c/4e hyperbonding occurs through in-phase ($\hat{\sigma}_{PP}$)

superposition of the terminal phosphorus atoms in P–Pd–P triads, which are populated by two electrons. Stabilization of the entire triad is achieved by the delocalization of the additional lone pair on the central Pd-atom ($n_{Pd} \rightarrow \hat{\sigma}^*_{PP}$).[18] No metallacyclopropane-like structure was found in the NRT analysis.

To gain further insight into the long-bond present an additional NBO analysis was performed. Selected data is summarized in **Table S**. Five lone pairs of pure d-orbital character are located on the Pd central atom and the 5s orbital forms a highly polarized Pd–P4 single bond (88.92% contribution of P4). The P2–P5 long-bond is also significantly polarized with a coefficient of nearly 70% for P5. The P2 orbital contributing to the long-bond exhibits p-character. As described in the literature the long-bond is stabilized by delocalization of a lone pair of suitable symmetry located on the central atom. Indeed, a donor-acceptor (DA) interaction energy of 67.44 kcal/mol is found for $LP(5) \rightarrow \hat{\sigma}^*_{P2-P5}$ interaction.[18] Additionally, a DA interaction of the same order of magnitude is found for the Pd-P4 single bond. The P3 p-type lone pair exhibits two significant DA interactions with the σ^*_{Pd-P4} (74.73 kcal/mol) and $\hat{\sigma}^*_{P2-P5}$ (19.68 kcal/mol), suggesting a very flexible bonding environment for the Pd atom, which is further emphasized by the large number of resonance structures.

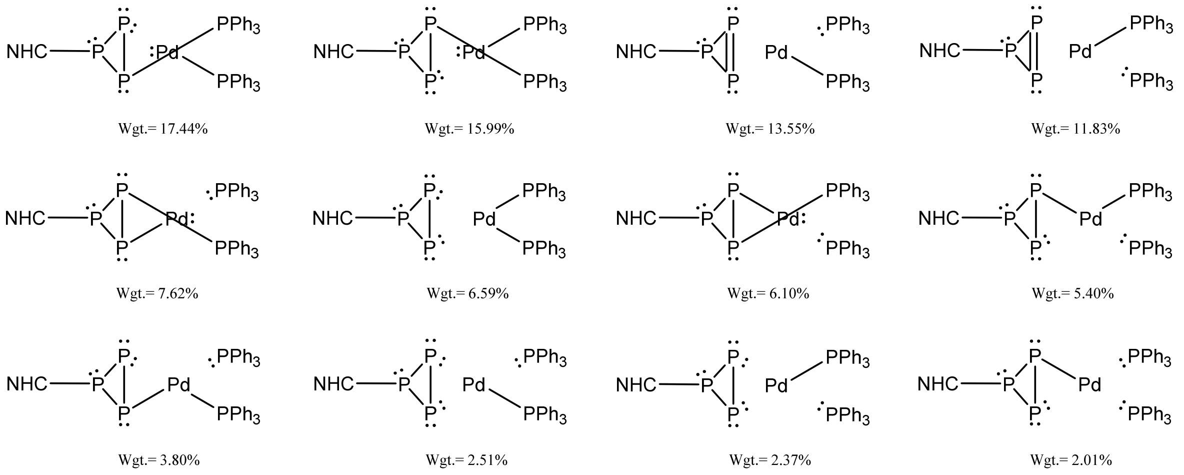

Fig. S41. Dominant resonance structures of 25⁺ (PBE0-D3BJ/def2TZVP) obtained with NRT (LP on Pd only shown in case of long-bonding.

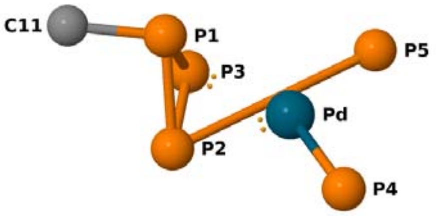

Fig. S42. Dominant resonance structure of 25^+ (PBE0-D3BJ/def2TZVP) obtained with NRT showing general numbering scheme.

Table S15. Selected NBO data of the dominating resonance structure of **25**$^{+}$ (PBE0-D3BJ/def2TZVP).

Atom 1	Atom 2	Type	Occupation	Hybrid 1 (coeff.)	Hybrid 2 (coeff.)	Acceptor	$E^{(2)}$ in kcal/mol
Pd	-	LP(1)	1.97	d (100%)	-	BD*(1) Pd–P4	10.66
Pd	-	LP(2)	1.97	d (100%)	-	BD*(1) Pd–P4	4.02
Pd	-	LP(3)	1.96	d (100%)	-	-	-
Pd	-	LP(4)	1.95	d (100%)	-	-	-
Pd	-	LP(5)	1.62	d (100%)	-	BD*(1) P2-P5	67.44
P1	-	LP	1.94	$sp^{0.44}$ (100%)	-	BD*(2) N10–C11	7.21
P2	-	LP	1.97	$sp^{0.28}$ (100%)	-	BD*(1) Pd–P4	7.96
P3	-	LP(1)	1.97	$sp^{0.28}$ (100%)	-	-	-
P3	-	LP(2)	1.23	$sp^{22.14}$ (100%)	-	BD*(1) Pd–P4	74.73
						BD*(1) P2–P5	19.68
Pd	P4	BD(1)	1.74	s (11.08%)	$sp^{2.19}$ (88.92%)	BD*(1) P2–P5	67.66
P1	P2	BD(1)	1.94	$sp^{9.5}$ (55.23%)	$sp^{12.64}$ (44.77%)	-	-
P1	P3	BD(1)	1.95	$sp^{8.82}$ (55.90%)	$sp^{11.39}$ (44.10%)	-	-
P1	C11	BD(1)	1.97	$sp^{7.83}$ (32.40%)	$sp^{1.54}$ (67.60%)	-	-
P2	P3	BD(1)	1.98	$sp^{8.92}$ (50.23%)	$sp^{9.08}$ (49.77%)	-	-
P2	P5	BD(1)	1.85	$sp^{19.35}$ (30.14%)	$sp^{2.25}$ (69.86%)	BD*(1) Pd–P4	16.6

5. References

(1) Holthausen, M. H.; Surmiak, S. K.; Jerabek, P.; Frenking, G.; Weigand, J. J. [3+2] Fragmentation of an [RP 5 Cl] + Cage Cation Induced by an N-Heterocyclic Carbene. *Angew. Chemie Int. Ed.* **2013**, *52* (42), 11078–11082. https://doi.org/10.1002/anie.201302914.

(2) Arduengo, A. J.; Krafczyk, R.; Schmutzler, R.; Craig, H. A.; Goerlich, J. R.; Marshall, W. J.; Unverzagt, M. Imidazolylidenes, Imidazolinylidenes and Imidazolidines. *Tetrahedron* **1999**, *55* (51), 14523–14534. https://doi.org/10.1016/S0040-4020(99)00927-8.

(3) Andreev, I. A.; Boichenko, M. A.; Ratmanova, N. K.; Ivanova, O. A.; Levina, I. I.; Khrustalev, V. N.; Sedov, I. A.; Trushkov, I. V. 4-(Dimethylamino)Pyridinium Azide in Protic Ionic Liquid Media as a Stable Equivalent of Hydrazoic Acid. *Adv. Synth. Catal.* **2022**, *364* (14), 2403–2415. https://doi.org/10.1002/adsc.202200486.

(4) Yogendra, S.; Schulz, S.; Hennersdorf, F.; Kumar, S.; Fischer, R.; Weigand, J. J. Reductive Ring Opening of a Cyclo-Tri(Phosphonio)Methanide Dication to a Phosphanylcarbodiphosphorane: In Situ UV-Vis Spectroelectrochemistry and Gold Coordination. *Organometallics* **2018**, *37* (5), 748–754. https://doi.org/10.1021/acs.organomet.7b00597.

(5) Ramey, K. C.; Louick, D. J.; Whitehurst, P. W.; Wise, W. B.; Mukherjee, R.; Moriarty, R. M. A Line Width Method for Determining Chemical Exchange Rates from NMR Spectra. *Org. Magn. Reson.* **1971**, *3* (2), 201–216. https://doi.org/10.1002/mrc.1270030206.

(6) Oxford Diffraction / Agilent Technologies UK Ltd. CrysAlisPRO. Oxford Diffraction / Agilent Technologies UK Ltd: Yarnton, England 2016.

(7) Dolomanov, O. V.; Bourhis, L. J.; Gildea, R. J.; Howard, J. A. K.; Puschmann, H. OLEX2 : A Complete Structure Solution, Refinement and Analysis Program. *J. Appl. Crystallogr.* **2009**, *42* (2), 339–341. https://doi.org/10.1107/S0021889808042726.

(8) Sheldrick, G. M. SHELXT – Integrated Space-Group and Crystal-Structure Determination. *Acta Crystallogr. Sect. A Found. Adv.* **2015**, *71* (1), 3–8. https://doi.org/10.1107/S2053273314026370.

(9) Sheldrick, G. M. Crystal Structure Refinement with SHELXL. *Acta Crystallogr. Sect. C Struct. Chem.* **2015**, *71* (1), 3–8. https://doi.org/10.1107/S2053229614024218.

(10) Neese, F. The ORCA Program System. *WIREs Comput. Mol. Sci.* **2012**, *2* (1), 73–78. https://doi.org/10.1002/wcms.81.

(11) Neese, F. Software Update: The ORCA Program System, Version 4.0. *WIREs Comput. Mol. Sci.* **2018**, *8* (1). https://doi.org/10.1002/wcms.1327.

(12) Frisch, M. J.; Trucks, G. W.; Schlegel, H. B.; Scuseria, G. E.; Robb, M. A.; Cheeseman, J. R.; Scalmani, G.; Barone, V.; Petersson, G. A.; Nakatsuji, H.; Li, X.; Caricato, M.; Marenich, A. V.; Bloino, J.; Janesko, B. G.; Gomperts, R.; Mennucci, B.; Hratchian, H. P.; Ortiz, J. V.; Izmaylov, A. F.; Sonnenberg, J. L.; Williams-Young, D.; Ding, F.; Lipparini, F.; Egidi, F.; Goings, J.; Peng, B.; Petrone, A.; Henderson, T.; Ranasinghe, D.; Zakrzewski, V. G.; Gao, J.; Rega, N.; Zheng, G.; Liang, W.; Hada, M.; Ehara, M.; Toyota, K.; Fukuda, R.; Hasegawa, J.; Ishida, M.; Nakajima, T.; Honda, Y.; Kitao, O.; Nakai, H.; Vreven, T.; Throssell, K.; Montgomery, J. A., J.; Peralta, J. E.; Ogliaro, F.; Bearpark, M. J.; Heyd, J. J.; Brothers, E. N.; Kudin, K. N.; Staroverov, V. N.; Keith, T. A.; Kobayashi, R.; Normand, J.; Raghavachari, K.; Rendell, A. P.; Burant, J. C.; Iyengar, S. S.; Tomasi,

J.; Cossi, M.; Millam, J. M.; Klene, M.; Adamo, C.; Cammi, R.; Ochterski, J. W.; Martin, R. L.; Morokuma, K.; Farkas, O. B.; Foresman, J.; Fox, D. J. Gaussian. Gaussian, Inc., Wallingford CT, 2016. 2016, p Revision C.01, Gaussian, Inc., Wallingford CT.
(13) Adamo, C.; Barone, V. Toward Reliable Density Functional Methods without Adjustable Parameters: The PBE0 Model. *J. Chem. Phys.* **1999**, *110* (13), 6158–6170. https://doi.org/10.1063/1.478522.
(14) Grimme, S.; Ehrlich, S.; Goerigk, L. Effect of the Damping Function in Dispersion Corrected Density Functional Theory. *J. Comput. Chem.* **2011**, *32* (7), 1456–1465. https://doi.org/10.1002/jcc.21759.
(15) Glendening, E. D.; Landis, C. R.; Weinhold, F. NBO 6.0 : Natural Bond Orbital Analysis Program. *J. Comput. Chem.* **2013**, *34* (16), 1429–1437. https://doi.org/10.1002/jcc.23266.
(16) Keith, T. A. AIMAll. 2019, p AIMAll, TK Gristmill Software, Overland Park KS, U.
(17) Mitoraj, M. P.; Michalak, A.; Ziegler, T. A Combined Charge and Energy Decomposition Scheme for Bond Analysis. *J. Chem. Theory Comput.* **2009**, *5* (4), 962–975. https://doi.org/10.1021/ct800503d.
(18) a) C. R. Landis and F. Weinhold, 3c/4e s-type long-bonding: a novel transitional motif toward the metallic delocalization limit, *Inorg. Chem.*, **2013**, *52*, 5154; b) F. Weinhold, C. R. Landis and E. D. Glendening, What is NBO analysis and how is it useful, *Int. Rev. Phys. Chem.*, **2016**, *35*, 399.
(19) Landis, C. R.; Weinhold, F. 3c/4e Σ-Type Long-Bonding: A Novel Transitional Motif toward the Metallic Delocalization Limit. *Inorg. Chem.* **2013**, *52* (9), 5154–5166. https://doi.org/10.1021/ic4000395.
